# Synthesis of Oxadiazole-Thiadiazole Hybrids and Their Anticandidal Activity

**DOI:** 10.3390/molecules22112004

**Published:** 2017-11-18

**Authors:** Serkan Levent, Betül Kaya Çavuşoğlu, Begüm Nurpelin Sağlık, Derya Osmaniye, Ulviye Acar Çevik, Özlem Atlı, Yusuf Özkay, Zafer Asım Kaplancıklı

**Affiliations:** 1 Department of Pharmaceutical Chemistry, Faculty of Pharmacy, Anadolu University, 26470 Eskişehir, Turkey; serkanlevent@anadolu.edu.tr (S.L.); betulkaya@anadolu.edu.tr (B.K.Ç.); bnsaglik@anadolu.edu.tr (B.N.S.); dosmaniye@anadolu.edu.tr (D.O.); uacar@anadolu.edu.tr (U.A.Ç.); zakaplan@anadolu.edu.tr (Z.A.K.); 2 Doping and Narcotic Compounds Analysis Laboratory, Faculty of Pharmacy, Anadolu University, 26470 Eskişehir, Turkey; 3 Department of Pharmaceutical Toxicology, Faculty of Pharmacy, Anadolu University, 26470 Eskişehir, Turkey; oatli@anadolu.edu.tr

**Keywords:** 1,3,4-oxadiazole, 1,3,4-thiadiazole, antifungal activity, ergosterol, cytotoxicity, docking

## Abstract

In the field of infection management, it is a major challenge to discover a potent and safe antifungal agent due to the emergence of resistant strains. Hence, the goal of this paper is to design and synthesize novel oxadiazole-thiadiazole hybrid compounds (**6a**–**6s**) and evaluate their antifungal activity. The structures of synthesized compounds were elucidated by various methods including FT-IR, ^1^H-NMR, ^13^C-NMR and HR-MS spectral data. Compounds were tested against four *Candida* species by broth microdilution assay. Compounds **6e**, **6k** and **6r**, bearing a nitro group, showed significant antifungal activity against all fungi with minimum inhibitory concentration (MIC) in the range of 0.78–3.12 µg/mL. These compounds were also screened for their in vitro cytotoxic effects by MTT assay and detected as nontoxic at their active concentrations against *Candida* strains. To examine the effects of these compounds on ergosterol biosynthesis, the LC-MS-MS method, which is based on quantification of ergosterol level in *C. krusei*, was carried out. Finally, the most active molecule (**6e**) was docked in the active site of the lanosterol 14α-demethylase enzyme, and it was determined that there is a strong interaction between the compound and enzyme.

## 1. Introduction

Fungal infections represent a challenging problem worldwide. High prevalence of antimicrobial resistance of pathogenic micro-organisms to conventional drugs reduces the efficacy of treatment, requiring the development of alternative agents [1]. Among many drugs in clinic, azole antifungals are commonly employed in the therapy of fungal diseases. Their mechanism of action is to inhibit the lanosterol 14α-demethylase (CYP51) in yeast and fungi [2]. CYP51 is a key enzyme in fungal membrane ergosterol biosynthesis, and azoles, thereby, cause depletion of ergosterol and resulting in inhibition of fungal growth [3].

It is well-known that azole derivatives represent a series of synthetic compounds of remarkable medicinal importance. Azole antifungal agents are of ample interest due to their broad spectrum-activity, high therapeutic index and low toxicity [4]. Among a large variety of azole derivatives, 1,3,4-oxadiazole compounds were reported to possess various biological activities including antifungal activity [5,6,7,8,9]. The –N=C–O– linkage in oxadiazoles can react with the nucleophilic centers of microbial cells and is attributed with its biological activity [10]. Its electronic and charge-transporting properties ensure connection with targets through non-covalent interactions. Oxadiazoles have been described as bioisosteres for carboxylic acids, esters and carboxamides [11]. These characteristics of the 1,3,4-oxadiazole ring resulted in the diverse pharmaceutical applications of these molecules in the field of medicinal chemistry.

1,3,4-Thiadiazole, containing hydrogen-binding domain, sulfur atom, and two-electron donor nitrogen system, is an important class of the five-membered heterocyclic family. The wide range of therapeutic values of this ring systems encourage researchers to perform an extensive research on the synthesis of new potent antifungal agents that contain the 1,3,4-thiadiazole ring in their structures. Due to the bioisosterism between oxygen and sulphur atoms, 1,3,4-thiadiazoles possess similar physical and chemical properties to 1,3,4-oxadiazoles. The extensive literature survey revealed that 1,3,4-thiadiazole derivatives exhibit antifungal activity [12,13,14,15,16].

The combination of two or more pharmacophores in one molecule is a strategy in the search for new antifungal agents, due to a broader antimicrobial spectrum and the potential to slow the development of drug resistance [17]. Thus, we planned to combine both pharmacophores into a compact system to obtain a new class of antifungal agents. The hybrid 1,3,4-oxadiazole-1,3,4-thiadiazole compounds were linked via a thioethyl chain and substituted with different aliphatic and aromatic rings in order to investigate the effects of structural variation on biological activity.

## 2. Results and Discussion

### 2.1. Chemistry

**Scheme 1:**
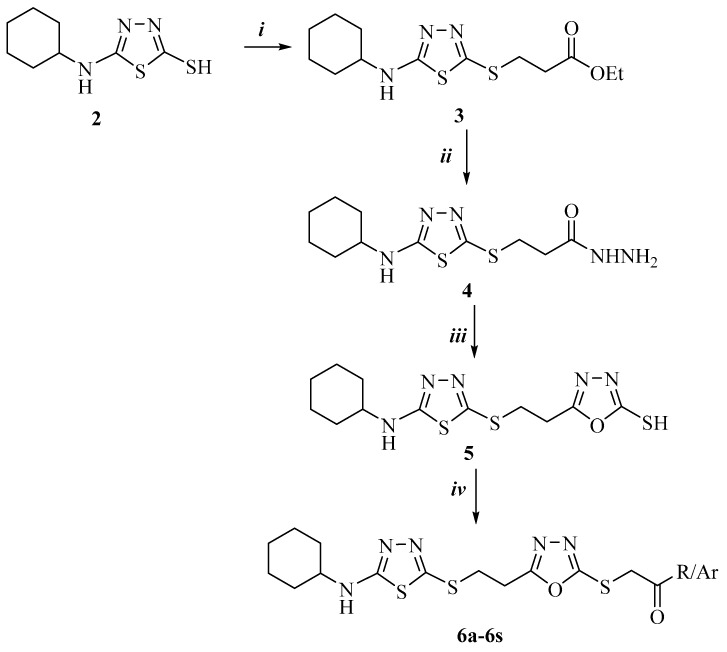
The synthesis of the compounds (**6a**–**6s**). *Reagents and conditions*: (***i***) BrCH_2_CH_2_COOEt, K_2_CO_3_, acetone, reflux, 9 h; (***ii***) NH_2_NH_2_·H_2_O, ethanol, rt, 4 h; (***iii***) (1) CS_2_/NaOH, ethanol, reflux, 10 h; (2) HCl, pH: 4–5; (***iv***) 4-substitutedphenacyl bromides (**6a**–**6f**), 2-chloro-*N*-(4-substitutedphenyl)acetamides (**6g**–**6l**), or 2-chloro-*N*-(6-substitutedbenzothiazol-2-yl)acetamides (**6m**–**6s**), K_2_CO_3_, acetone, rt.

Title compounds (**6a**–**6s**) were synthesized according to the pathway as outlined in Scheme 1. The thiosemicarbazide derivative (**1**) was obtained through the reaction of cyclohexyl isothiocyanate and hydrazine hydrate. The ring closure reaction of compound **1** with carbon disulfide in the presence of sodium hydroxide gave 5-(cyclohexylamino)-1,3,4-thiadiazole-2-thiol (**2**) which then was reacted with ethyl bromopropionate to obtain ethyl 3-[[5-(cyclohexylamino)-1,3,4-thiadiazol-2-yl]thio]propanoate (**3**). 3-[[5-(Cyclohexylamino)-1,3,4-thiadiazol-2-yl]thio]propanehydrazide (**4**) was synthesized via the treatment of ester (**3**) with hydrazine hydrate. The second ring closure reaction of the corresponding hydrazide (**4**) with carbon disulfide resulted in the formation of 5-[2-[[5-(cyclohexylamino)-1,3,4-thiadiazol-2-yl]thio]ethyl]-1,3,4-oxadiazole-2-thiol (**5**). Finally, compound **5** was reacted with appropriate 4-substituted phenacyl bromides, 4-substituted anilines or 6-substituted-2-aminobenzothiazoles to afford the target compounds (**6a**–**6s**).

The formation of synthesized compounds (**6a**–**6s**) was confirmed by IR, mass spectra, ^1^H-NMR and ^13^C-NMR. In the IR spectra of all synthesized compounds, bands at ~3340 cm^−1^, ~3070 cm^−1^, ~1580 cm^−1^ and ~1480 cm^−1^ were attributed to the stretching of the N-H, aromatic C-H, C=N and C=C groups. The carbonyl band belonging to ketone group was observed between 1660–1701 cm^−1^. The compounds **6e**, **6k** and **6r** showed two bands around ~1520 cm^−1^ and ~1330 cm^−1^ due to asymmetric and symmetric stretching of the NO_2_ group. The ^1^H-NMR of all synthesized compounds showed peaks at ~3.25 ppm (SCH_2_CH_2_) and ~3.41 ppm (-SCH_2_CH_2_-) due to the methylene protons of thioethyl moiety. The signals of oxoethyl moiety (COCH_2_) were resonated at ~5.09 ppm, ~4.27 ppm and ~4.41 ppm as singlets for compounds **6a**–**6f**, **6g**–**6l** and **6m**–**6s**, respectively. For all compounds, the NH proton bonded to cyclohexyl ring appeared at ~7.79 ppm as a doublet peak. A singlet peak belonging to the NH proton of acetamide moiety (-NHCOCH_2_) was designated at ~10.38 ppm in compounds **6g**–**6l** and ~12.76 ppm in compounds **6m**–**6s**. In the ^13^C-NMR of compounds, the signal due to the carbonyl group appeared at ~192.50 ppm for compounds **6a**–**6f** and at ~169.20 ppm for compounds **6g**–**6s**. The spectra of all compounds showed peaks in the range of ~105–169 ppm due to the presence of aromatic carbons. The mass spectrum of all the compounds were in full accordance with their molecular weights. The IR, ^1^H-NMR, ^13^C-NMR, and HRMS spectra of all compounds are presented in supplementary file.

### 2.2. Antifungal Activity Assay

The results of anticandidal activity of the synthesized compounds **6a**–**6s** and reference drugs are presented with minimum inhibitory concentration (MIC_50_; µg/mL) values in Table 1. Minimum concentrations of compounds **6a**–**6s** that inhibited the growth of yeast strains ranged from 0.78 to 100 µg/mL. According to antifungal screening, compounds **6e**, **6k** and **6r** were found to be active against all tested fungi. Compound **6e** indicated similar antifungal activity to ketoconazole against all *Candida* strains and was evaluated as the most active derivative in the series. This compound showed higher anticandidal activity than ketoconazole against *C. krusei* and *C. parapsilopsis* with an MIC_50_ value of 0.78 µg/mL. Compounds **6e** and **6k** showed equal levels of activity as ketoconazole against *C. glabrata*. Also these compounds showed half as much activity as ketoconazole against *C. albicans*. Compound **6r** exhibited equal level of activity with ketoconazole against *C. krusei* and *C. parapsilopsis* with an MIC_50_ value of 1.56 µg/mL. The most sensitive yeast to all compounds was *C. parapsilopsis*.

In compounds **6a**–**6f**, the introduction of a chloro atom in the 4 position of phenyl ring improved activity against *C. krusei*, compared to 4-methoxy derivative. In compounds **6g**–**6l**, the effects of 4-methoxy and 4-methyl groups on the compound’s activity were the same against all strains. A common structural feature of the most active compounds **6e**, **6k**, and **6r** is the presence of a nitro substituent. Hence, it can be assumed that the introduction of a nitro group is highly beneficial for antifungal activity [18,19,20]. It is possible to note a correlation between antifungal activity with an electron withdrawing nitro group because the substition of this group affected pKa, hydrophobic interactions and also lipophilicity, which are citical to enhanced biological activity.

### 2.3. Effect of Compounds **6e**, **6k**, and **6r** on Ergosterol Content of C. krusei

Compounds **6e**, **6k** and **6r** were selected for the second step of the study due to their potent activity against tested *Candida* strains. In this step, these compounds were tested for their effect on the ergosterol level of *C. krusei*. Inhibition of ergosterol biosynthesis was measured at their 0.78 µg/mL, 1.56 µg/mL, 3.12 µg/mL concentrations and expressed as (%) inhibition. The results are respresented in Table 2. All tested compounds inhibited ergosterol biosynthesis, concentration-dependently. The results were comparable to the reference drug ketoconazole. At all tested concentrations, the (%) inhibition of compound **6e** was higher than that of the reference. These results provide further support for the hypothesis that compounds **6e**, **6k** and **6r** exhibited their antifungal activity through the inhibition of ergosterol biosynthesis.

### 2.4. Cytotoxicity Test

In drug discovery, cytotoxicity is an important and essential criteria for a drug candidate. MTT assay was carried out for screening the cytotoxic effects of the compounds [21]. The IC_50_ values of the compounds are indicated in Table 2. The IC_50_ values of compounds **6e** and **6k** were recorded above 500 µg/mL and that of compound **6r** was found to be 406.066 µg/mL. It can thus be suggested that the tested compounds are nontoxic at their active concentrations against *Candida* strains.

### 2.5. Molecular Docking Studies

**Figure 1:**
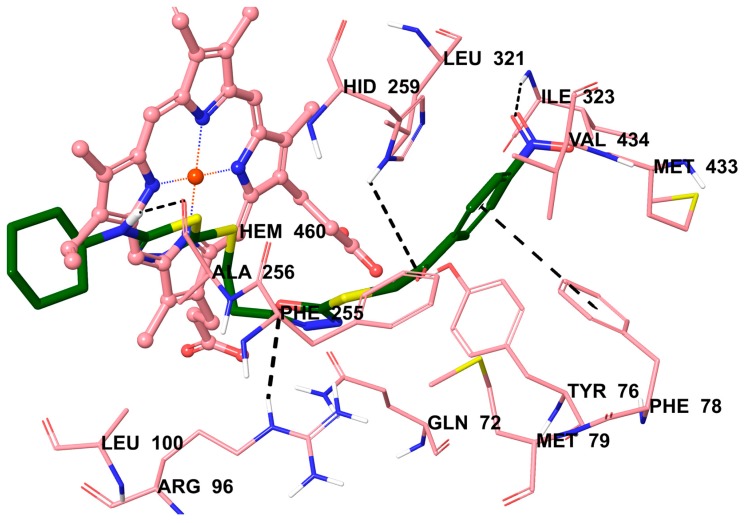
The interacting mode of compound **6e** in the active region of 14 alpha-sterol demethylase. The inhibitor is colored with dark green and HEM is colored with pink.

14-α-Sterol demethylase is a key enzyme in the membrane ergosterol biosynthesis of fungi. In docking studies, the binding modes of compound **6e** to 14-α-sterol demethylase was clarified. Studies were performed with the X-ray crystal structure of 14-α-sterol demethylase from Mycobacterium tuberculosis in complex with fluconazole (Protein Data Bank (PDB) ID: 1EA1) [22]. The docking pose on 14-α-sterol demethylase reveals that the interactions between the compound **6e** and amino acid residues are very important in terms of binding to the active site of enzyme (Figure 1). It is seen that there are two π-π interactions. The first π-π interaction is between oxadiazole ring and Arg96, while the second is between phenyl and phenyl of Phe78. The amino of the structure, between cyclohexyl and the thiadiazole ring establishes a hydrogen with the carbonyl of Ala256. Also, there is another hydrogen bond between the carbonyl group and the amino of Hid259. An additional interaction is related to the nitro substituent. The nitro group, at C-4 of phenyl, is in interaction with the amino of Ile323 by hydrogen bonding. These interactions explain the reason for the stronger anticandidal activity of **6e**. It can be suggested that electron withdrawing groups such as the nitro at C-4 of phenyl is very important in terms of binding to enzyme active site and anticandidal activity. As a result, it is considered that this additional interaction could explain the higher binding capability and stronger activity of compound **6e**, as compared with the other compounds.

## 3. Materials and Methods

### 3.1. Chemistry

All chemicals were purchased from Sigma-Aldrich Chemical Co (Sigma-Aldrich Corp., St. Louis, MO, USA) and Merck Chemicals (Merck KGaA, Darmstadt, Germany). All melting points (m.p.) were determined by Electrothermal 9100 digital melting point apparatus (Electrothermal, Essex, UK) and are uncorrected. All the reactions were monitored by thin-layer chromatography (TLC) using Silica Gel 60 F254 TLC plates (Merck KGaA, Darmstadt, Germany). Spectroscopic data were recorded with the following instruments: IR, Shimadzu 8400S spectrophotometer (Shimadzu, Tokyo, Japan); ^1^H-NMR, Bruker DPX 300 NMR spectrometer (Bruker Bioscience, Billerica, MA, USA), ^13^C-NMR, Bruker DPX 75 NMR spectrometer (Bruker Bioscience, Billerica, MA, USA) in DMSO-*d*_6_, using TMS as the internal standard; M + 1 peaks were determined by Shimadzu LC/MS ITTOF system (Shimadzu, Tokyo, Japan).

#### 3.1.1. Synthesis of *N*-Cyclohexylhydrazinecarbothioamide (**1**)

Cyclohexyl isothiocyanate (0.1 mol, 15 mL) and hydrazine hydrate (0.2 mol) were stirred in ethanol (150 mL) at room temperature. After the completion of the reaction, the precipitated product (**1**) was filtered and recrystallized from ethanol [23].

#### 3.1.2. Synthesis of 5-(Cyclohexylamino)-1,3,4-thiadiazole-2-thiol (**2**) 

Compound **1** (0.09 mol) was dissolved in ethanol (150 mL) and a solution of NaOH (0.1 mol) in ethanol was added. Carbon disulfide (0.1 mol, 6 mL) was added and the mixture was refluxed for 8 h. After this period, the solution was cooled and acidified to pH4–5 with hydrochloric acid solution and crystallized from ethanol [23].

#### 3.1.3. Synthesis of Ethyl 3-[[5-(Cyclohexylamino)-1,3,4-thiadiazol-2-yl]thio]propanoate (**3**)

A mixture of compound **2** (0.08 mol), ethyl bromopropionate (0.08 mol) and potassium carbonate (0.09 mol) was stirred under reflux for 9 h in acetone (150 mL). The solvent was evaporated under reduced pressure and the product was washed with water, dried and crystallized from ethanol [23].

#### 3.1.4. Synthesis of 3-[[5-(Cyclohexylamino)-1,3,4-thiadiazol-2-yl)thio]propanehydrazide (**4**)

Compound **3** (0.06 mol) and hydrazine hydrate (0.06 mol) were stirred in ethanol (150 mL) at room temperature for 4 h and filtered [23]. 

#### 3.1.5. Synthesis of 5-[2-[[5-(Cyclohexylamino)-1,3,4-thiadiazol-2-yl]thio]ethyl]-1,3,4-oxadiazole-2-thiol (**5**)

Compound **4** (0.05 mol) was added to a solution of NaOH (0.06 mol) in ethanol (100 mL). After the addition of carbon disulfide (0.06 mol), the mixture was heated under reflux for 10 h. The solution was cooled and acidified to pH4–5 with hydrochloric acid solution and recrystallized from ethanol [24].

#### 3.1.6. Synthesis of 2-[[5-[2-[[5-(Cyclohexylamino)-1,3,4-thiadiazol-2-yl]thio]ethyl]-1,3,4-oxadiazol-2-yl]thio]-1-substituted ethan-1-one (**6a**–**6s**)

Compound **5** (2 mmol) and appropriate 4-substituted phenacyl bromide (2 mmol), 4-substituted aniline (2 mmol) or 6-substituted 2-amino benzothiazole (2 mmol) were stirred in acetone (40 mL) at room temperature in the presence of potassium carbonate (2.4 mmol). Acetone was evaporated under reduced pressure and the product was washed with water, dried and crystallized from ethanol.

*2-[[5-[2-[[5-(Cyclohexylamino)-1,3,4-thiadiazol-2-yl]thio]ethyl]-1,3,4-oxadiazol-2-yl]thio]-1-phenylethan-1-one* (**6a**): Yield 67%, m.p. 141 °C. IR *ν*_max_ (cm^−1^): 3340 (N-H) 3064 (aromatic C-H), 2933 (aliphatic C-H), 1681 (C=O ketone), 1587–1487 (C=N, C=C), 1197–1085 (C-N, C-O). ^1^H-NMR (300 MHz, DMSO-*d*_6_, ppm) δ 1.14–1.30 (5H, m, cyclohexyl-H), 1.53–1.56 (1H, m, cyclohexyl-H), 1.66–1.70 (2H, m, cyclohexyl-H), 1.91–1.95 (2H, m, cyclohexyl-H), 3.25 (2H, t, *J* = 6.6 Hz, SCH_2_CH_2_), 3.41 (2H, t, *J* = 7.4 Hz, SCH_2_CH_2_), 3.46–3.52 (1H, m, cyclohexyl-H), 5.09 (2H, s, COCH_2_), 7.58 (2H, t, *J* = 7.8 Hz, Ar-H), 7.71 (1H, t, *J* = 7.3 Hz, Ar-H), 7.79 (1H, d, *J* = 7.3 Hz, NH), 8.04 (2H, d, *J* = 8.5 Hz, Ar-H). ^13^C-NMR (75 MHz, DMSO-*d*_6_, ppm) δ 24.67, 25.65, 26.08, 31.04, 32.46, 41.04, 54.00, 128.91, 129.36, 134.45, 166.34, 169.19, 192.99. HR-MS (*m*/*z*): [M + H]^+^ calcd for C_20_H_23_N_5_O_2_S_3_: 462.1087; found: 462.1089.

*2-[[5-[2-[[5-(Cyclohexylamino)-1,3,4-thiadiazol-2-yl]thio]ethyl]-1,3,4-oxadiazol-2-yl]thio]-1-(4-methyl phenyl)ethan-1-one* (**6b**): Yield 70%, m.p. 136 °C. IR *ν*_max_ (cm^−1^): 3327 (N-H) 3055 (aromatic C–H), 2926 (aliphatic C–H), 1678 (C=O ketone), 1589–1481 (C=N, C=C), 1298–1089 (C-N, C-O). ^1^H-NMR (300 MHz, DMSO-*d*_6_, ppm) δ 1.14–1.30 (5H, m, cyclohexyl-H), 1.53–1.56 (1H, m, cyclohexyl-H), 1.65–1.70 (2H, m, cyclohexyl-H), 1.91–1.95 (2H, m, cyclohexyl-H), 2.40 (3H, s, CH_3_), 3.25 (2H, t, *J* = 6.5 Hz, SCH_2_CH_2_), 3.41 (2H, t, *J* = 7.0 Hz, SCH_2_CH_2_), 3.45–3.51 (1H, m, cyclohexyl-H), 5.05 (2H, s, COCH_2_), 7.38 (2H, d, *J* = 8.0 Hz, Ar-H), 7.79 (1H, d, *J* = 7.3 Hz, NH), 7.94 (2H, d, *J* = 8.3 Hz, Ar-H). ^13^C-NMR (75 MHz, DMSO-*d*_6_, ppm) δ 21.71, 24.67, 25.65, 26.08, 31.05, 32.47, 41.00, 54.00, 129.03, 129.89, 132.99, 145.03, 148.52, 163.53, 166.31, 169.18, 192.48 (C=O). HR-MS (*m*/*z*): [M + H]^+^ calcd for C_21_H_25_N_5_O_2_S_3_: 476.1243; found: 476.1241.

*2-[[5-[2-[[5-(Cyclohexylamino)-1,3,4-thiadiazol-2-yl]thio]ethyl]-1,3,4-oxadiazol-2-yl]thio]-1-(4-methoxyphenyl)ethan-1-one* (**6c**): Yield 75%, m.p. 120 °C. IR *ν*_max_ (cm^−1^): 3338 (N-H) 3076 (aromatic C–H), 2927 (aliphatic C–H), 1660 (C=O ketone), 1593–1483 (C=N, C=C), 1259–1095 (C-N, C-O). ^1^H-NMR (300 MHz, DMSO-*d*_6_, ppm) δ 1.16–1.30 (5H, m, cyclohexyl-H), 1.53–1.56 (1H, m, cyclohexyl-H), 1.65–1.70 (2H, m, cyclohexyl-H), 1.91–1.95 (2H, m, cyclohexyl-H), 3.25 (2H, t, *J* = 6.5 Hz, SCH_2_CH_2_), 3.39–3.48 (3H, m, SCH_2_CH_2_, cyclohexyl-H), 3.86 (3H, s, OCH_3_), 5.03 (2H, s, COCH_2_), 7.09 (2H, d, *J* = 8.9 Hz, Ar-H), 7.79 (1H, d, *J* = 7.3 Hz, NH), 8.02 (2H, d, *J* = 8.9 Hz, Ar-H). ^13^C-NMR (75 MHz, DMSO-*d*_6_, ppm) δ 24.67, 25.65, 26.08, 31.05, 32.46, 54.00, 56.12, 114.58, 128.32, 131.35, 148.52, 163.60, 164.21, 166.29, 169.18, 191.28. HR-MS (*m*/*z*): [M + H]^+^ calcd for C_21_H_25_N_5_O_3_S_3_: 492.1187; found: 492.1192.

*2-[[5-[2-[[5-(Cyclohexylamino)-1,3,4-thiadiazol-2-yl]thio]ethyl]-1,3,4-oxadiazol-2-yl]thio]-1-(4-chlorophenyl)ethan-1-one* (**6d**): Yield 74%, m.p. 167 °C. IR *ν*_max_ (cm^−1^): 3336 (N-H) 3057 (aromatic C–H), 2929 (aliphatic C–H), 1680 (C=O ketone), 1587–1481 (C=N, C=C), 1197–1087 (C-N, C-O). ^1^H-NMR (300 MHz, DMSO-*d*_6_, ppm) δ 1.10–1.35 (5H, m, cyclohexyl-H), 1.53–1.56 (1H, m, cyclohexyl-H), 1.66–1.70 (2H, m, cyclohexyl-H), 1.91–1.95 (2H, m, cyclohexyl-H), 3.25 (2H, t, *J* = 6.9 Hz, SCH_2_CH_2_), 3.39–3.52 (3H, m, SCH_2_CH_2_, cyclohexyl-H), 5.09 (2H, s, COCH_2_), 7.65 (2H, d, *J* = 8.6 Hz, Ar-H), 7.79 (1H, d, *J* = 7.3 Hz, NH), 8.05 (2H, d, *J* = 8.7 Hz, Ar-H). ^13^C-NMR (75 MHz, DMSO-*d*_6_, ppm) δ 24.67, 25.65, 26.07, 31.02, 32.46, 40.92, 54.01, 129.49, 130.84, 134.20, 139.37, 148.51, 163.38, 166.39, 169.18, 192.16. HR-MS (*m*/*z*): [M + H]^+^ calcd for C_20_H_22_ClN_5_O_2_S_3_: 496.0697; found: 496.0679.

*2-[[5-[2-[[5-(Cyclohexylamino)-1,3,4-thiadiazol-2-yl]thio]ethyl]-1,3,4-oxadiazol-2-yl]thio]-1-(4-nitrophenyl)ethan-1-one* (**6e**): Yield 77%, m.p. 144 °C. IR *ν*_max_ (cm^−1^): 3336 (N-H) 3068 (aromatic C–H), 2929 (aliphatic C–H), 1701 (C=O ketone), 1583–1481 (C=N, C=C), 1180–1051 (C-N, C-O). ^1^H-NMR (300 MHz, DMSO-*d*_6_, ppm) δ 1.11–1.35 (5H, m, cyclohexyl-H), 1.53–1.56 (1H, m, cyclohexyl-H), 1.66–1.70 (2H, m, cyclohexyl-H), 1.91–1.95 (2H, m, cyclohexyl-H), 3.26 (2H, t, *J* = 6.5 Hz, SCH_2_CH_2_), 3.35–3.49 (3H, m, SCH_2_CH_2_, cyclohexyl-H), 5.15 (2H, s, COCH_2_), 7.79 (1H, d, *J* = 7.3 Hz, NH), 8.27 (2H, d, *J* = 9.0 Hz, Ar-H), 8.39 (2H, d, *J* = 8.9 Hz, Ar-H). ^13^C-NMR (75 MHz, DMSO-*d*_6_, ppm) δ 24.67, 25.65, 26.05, 31.00, 32.46, 41.16, 54.01, 124.42, 130.37, 140.16, 148.51, 150.74, 163.23, 166.48, 169.18, 192.50. HRMS (*m*/*z*): [M + H]^+^ calcd for C_20_H_22_N_6_O_4_S_3_: 507.0937; found: 507.0924.

*2-[[5-[2-[[5-(Cyclohexylamino)-1,3,4-thiadiazol-2-yl]thio]ethyl]-1,3,4-oxadiazol-2-yl]thio]-1-(4-fluorophenyl)ethan-1-one* (**6f**): Yield 64%, m.p. 131 °C. IR *ν*_max_ (cm^−1^): 3342 (N-H) 3061 (aromatic C–H), 2929 (aliphatic C–H), 1680 (C=O ketone), 1587–1485 (C=N, C=C), 1232–1049 (C-N, C-O). ^1^H-NMR (300 MHz, DMSO-*d*_6_, ppm) δ 1.14–1.35 (5H, m, cyclohexyl-H), 1.53–1.56 (1H, m, cyclohexyl-H), 1.66–1.70 (2H, m, cyclohexyl-H), 1.91–1.95 (2H, m, cyclohexyl-H), 3.25 (2H, t, *J* = 6.3 Hz, SCH_2_CH_2_), 3.39-3.51 (3H, m, SCH_2_CH_2_, cyclohexyl-H), 5.08 (2H, s, COCH_2_), 7.41 (2H, t, *J* = 8.9 Hz, Ar-H), 7.79 (1H, d, *J* = 7.3 Hz, NH), 8.11–8.16 (2H, m, Ar-H). ^13^C-NMR (75 MHz, DMSO-*d*_6_, ppm) δ 24.67, 25.65, 26.07, 31.03, 32.46, 40.91, 54.01, 116.45 (d, *J* = 21.8 Hz), 132.04 (d, *J* = 9.8 Hz), 132. 28 (d, *J* = 3.5 Hz), 148.51, 163.43, 165.50 (d, *J* = 250.0 Hz), 167.57, 169.18, 191.68. HRMS (*m*/*z*): [M + H]^+^ calcd for C_20_H_22_FN_5_O_2_S_3_: 480.0992; found: 480.0987. 

*2-[[5-[2-[[5-(Cyclohexylamino)-1,3,4-thiadiazol-2-yl]thio]ethyl]-1,3,4-oxadiazol-2-yl]thio]-N-phenyl acetamide* (**6g**): Yield 72%, m.p. 177 °C. IR *ν*_max_ (cm^−1^): 3342 (N-H) 3057 (aromatic C–H), 2931 (aliphatic C–H), 1672 (C=O ketone), 1598–1483 (C=N, C=C), 1170–1093 (C-N, C-O). ^1^H-NMR (300 MHz, DMSO-*d*_6_, ppm) δ 1.10–1.36 (5H, m, cyclohexyl-H), 1.52–1.56 (1H, m, cyclohexyl-H), 1.66–1.70 (2H, m, cyclohexyl-H), 1.92–1.95 (2H, m, cyclohexyl-H), 3.26 (2H, t, *J* = 6.5 Hz, SCH_2_CH_2_), 3.39–3.53 (3H, m, SCH_2_CH_2_, cyclohexyl-H), 4.27 (2H, s, COCH_2_), 7.07 (1H, t, *J* = 7.4 Hz, Ar-H), 7.32 (2H, t, *J* = 7.6 Hz, Ar-H), 7.56 (2H, d, *J* = 7.6 Hz, Ar-H), 7.79 (1H, d, *J* = 7.3 Hz, NH), 10.38 (1H, s, NH). ^13^C-NMR (75 MHz, DMSO-*d*_6_, ppm) δ 24.67, 25.66, 26.10, 31.01, 32.47, 37.27, 54.02, 119.60, 124.12, 129.31, 139.10, 148.50, 163.58, 165.22, 166.38, 169.20. HR-MS (*m*/*z*): [M + H]^+^ calcd for C_20_H_24_N_6_O_2_S_3_: 477.1196; found: 477.1191.

*2-[[5-[2-[[5-(Cyclohexylamino)-1,3,4-thiadiazol-2-yl]thio]ethyl]-1,3,4-oxadiazol-2-yl]thio]-N-(4-methyl phenyl)acetamide* (**6h**): Yield 79%, m.p. 173 °C. IR *ν*_max_ (cm^−1^): 3336 (N-H) 3068 (aromatic C–H), 2916 (aliphatic C–H), 1668 (C=O ketone), 1521–1483 (C=N, C=C), 1240–1085 (C-N, C-O). ^1^H-NMR (300 MHz, DMSO-*d*_6_, ppm) δ 1.14–1.36 (5H, m, cyclohexyl-H), 1.52–1.56 (1H, m, cyclohexyl-H), 1.66–1.70 (2H, m, cyclohexyl-H), 1.92–1.96 (2H, m, cyclohexyl-H), 2.25 (3H, s, CH_3_), 3.26 (2H, t, *J* = 6.5 Hz, SCH_2_CH_2_), 3.39–3.50 (3H, m, SCH_2_CH_2_, cyclohexyl-H), 4.25 (2H, s, COCH_2_), 7.12 (2H, d, *J* = 8.3 Hz, Ar-H), 7.45 (2H, d, *J* = 8.4 Hz, Ar-H), 7.79 (1H, d, *J* = 7.3 Hz, NH), 10.29 (1H, s, NH). ^13^C-NMR (75 MHz, DMSO-*d*_6_, ppm) δ 20.91, 24.68, 25.66, 26.10, 31.01, 32.47, 37.25, 54.02, 119.61, 129.67, 133.08, 136.60, 148.51, 163.60, 164.95, 166.36, 169.20. HR-MS (*m*/*z*): [M + H]^+^ calcd for C_21_H_26_N_6_O_2_S_3_: 491.1352; found: 491.1343.

*2-[[5-[2-[[5-(Cyclohexylamino)-1,3,4-thiadiazol-2-yl]thio]ethyl]-1,3,4-oxadiazol-2-yl]thio]-N-(4-methoxy phenyl)acetamide* (**6i**): Yield 71%, m.p. 147 °C. IR *ν*_max_ (cm^−1^): 3336 (N-H) 3064 (aromatic C–H), 2920 (aliphatic C–H), 1662 (C=O ketone), 1514–1421 (C=N, C=C), 1286–1035 (C-N, C-O). ^1^H-NMR (300 MHz, DMSO-*d*_6_, ppm) δ 1.14–1.36 (5H, m, cyclohexyl-H), 1.52–1.56 (1H, m, cyclohexyl-H), 1.66–1.70 (2H, m, cyclohexyl-H), 1.92–1.95 (2H, m, cyclohexyl-H), 3.26 (2H, t, *J* = 6.5 Hz, SCH_2_CH_2_), 3.39–3.52 (3H, m, SCH_2_CH_2_, cyclohexyl-H), 3.72 (3H, s, OCH_3_), 4.23 (2H, s, COCH_2_), 6.89 (2H, d, *J* = 9.1 Hz, Ar-H), 7.47 (2H, d, *J* = 9.1 Hz, Ar-H), 7.79 (1H, d, *J* = 7.3 Hz, NH), 10.24 (1H, s, NH). ^13^C-NMR (75 MHz, DMSO-*d*_6_, ppm) δ 24.67, 25.66, 26.11, 31.02, 32.47, 37.17, 54.02, 55.61 (OCH_3_), 114.41, 121.17, 132.22, 148.51, 155.93, 163.61, 164.68, 166.36, 169.20. HR-MS (*m*/*z*): [M + H]^+^ calcd for C_21_H_26_N_6_O_3_S_3_: 507.1301; found: 507.1291.

*2-[[5-[2-[[5-(Cyclohexylamino)-1,3,4-thiadiazol-2-yl]thio]ethyl]-1,3,4-oxadiazol-2-yl]thio]-N-(4-chloro phenyl)acetamide* (**6j**): Yield 69%, m.p. 131 °C. IR *ν*_max_ (cm^−1^): 3338 (N-H) 3066 (aromatic C–H), 2933 (aliphatic C–H), 1668 (C=O ketone), 1583–1483 (C=N, C=C), 1238–1083 (C-N, C-O). ^1^H-NMR (300 MHz, DMSO-*d*_6_, ppm) δ 1.14–1.35 (5H, m, cyclohexyl-H), 1.52–1.57 (1H, m, cyclohexyl-H), 1.66–1.70 (2H, m, cyclohexyl-H), 1.92–1.95 (2H, m, cyclohexyl-H), 3.26 (2H, t, *J* = 6.5 Hz, SCH_2_CH_2_), 3.39–3.52 (3H, m, SCH_2_CH_2_, cyclohexyl-H), 4.27 (2H, s, COCH_2_), 7.38 (2H, d, *J* = 8.9 Hz, Ar-H), 7.60 (2H, d, *J* = 8.9 Hz, Ar-H), 7.79 (1H, d, *J* = 7.3 Hz, NH), 10.53 (1H, s, NH). ^13^C-NMR (75 MHz, DMSO-*d*_6_, ppm) δ 24.68, 25.66, 26.10, 31.00, 32.47, 37.19, 54.03, 60.71, 121.17, 127.69, 129.24, 138.04, 148.50, 163.51, 165.44, 166.41, 169.20. HR-MS (*m*/*z*): [M + H]^+^ calcd for C_20_H_23_ClN_6_O_2_S_3_: 511.0806; found: 511.0788.

*2-[[5-[2-[[5-(Cyclohexylamino)-1,3,4-thiadiazol-2-yl]thio]ethyl]-1,3,4-oxadiazol-2-yl]thio]-N-(4-nitrophenyl)acetamide* (**6k**): Yield 67%, m.p. 149 °C. IR *ν*_max_ (cm^−1^): 3340 (N-H) 3049 (aromatic C–H), 2939 (aliphatic C–H), 1697 (C=O ketone), 1616–1490 (C=N, C=C), 1521 and 1328 (NO_2_), 1159–1072 (C-N, C-O). ^1^H-NMR (300 MHz, DMSO-*d*_6_, ppm) δ 1.14–1.36 (5H, m, cyclohexyl-H), 1.53–1.56 (1H, m, cyclohexyl-H), 1.66–1.70 (2H, m, cyclohexyl-H), 1.92–1.95 (2H, m, cyclohexyl-H), 3.26 (2H, t, *J* = 6.5 Hz, SCH_2_CH_2_), 3.39–3.51 (3H, m, SCH_2_CH_2_, cyclohexyl-H), 4.33 (2H, s, COCH_2_), 7.78–7.84 (3H, m, NH, Ar-H), 8.24 (2H, d, *J* = 9.2 Hz, Ar-H), 11.00 (1H, s, NH). ^13^C-NMR (75 MHz, DMSO-*d*_6_, ppm) δ 24.67, 25.66, 26.08, 30.98, 32.46, 37.28, 54.02, 119.40, 125.57, 142.96, 145.16, 148.50, 163.41, 166.38, 166.47, 169.18. HRMS (*m*/*z*): [M + H]^+^ calcd for C_20_H_23_N_7_O_4_S_3_: 522.1046; found: 522.1035.

*2-[[5-[2-[[5-(Cyclohexylamino)-1,3,4-thiadiazol-2-yl]thio]ethyl]-1,3,4-oxadiazol-2-yl]thio]-N-(4-fluoro phenyl)acetamide* (**6l**): Yield 73%, m.p. 160 °C. IR *ν*_max_ (cm^−1^): 3342 (N-H) 3032 (aromatic C–H), 2854 (aliphatic C–H), 1670 (C=O ketone), 1583–1481 (C=N, C=C), 1168–1083 (C-N, C-O). ^1^H-NMR (300 MHz, DMSO-*d*_6_, ppm) δ 1.14–1.35 (5H, m, cyclohexyl-H), 1.52–1.56 (1H, m, cyclohexyl-H), 1.66–1.70 (2H, m, cyclohexyl-H), 1.91–1.95 (2H, m, cyclohexyl-H), 3.26 (2H, t, *J* = 6.5 Hz, SCH_2_CH_2_), 3.39–3.53 (3H, m, SCH_2_CH_2_, cyclohexyl-H), 4.25 (2H, s, COCH_2_), 7.16 (2H, t, *J* = 8.9 Hz, Ar-H), 7.56–7.60 (2H, m, Ar-H), 7.79 (1H, d, *J* = 7.3 Hz, NH), 10.45 (1H, s, NH). ^13^C-NMR (75 MHz, DMSO-*d*_6_, ppm) δ 24.67, 25.66, 26.10, 31.00, 32.47, 37.13, 54.02, 115.91 (d, *J* = 22.5 Hz), 121.42 (d, *J* = 8.3 Hz), 135.49 (d, *J* = 3.0 Hz), 148.50, 157.05, 161.89 (d, *J* = 248.0 Hz), 165.18, 166.39, 169.19. HR-MS (*m*/*z*): [M + H]^+^ calcd for C_20_H_23_FN_6_O_2_S_3_: 495.1101; found: 495.1087. 

*2-[[5-[2-[[5-(Cyclohexylamino)-1,3,4-thiadiazol-2-yl]thio]ethyl]-1,3,4-oxadiazol-2-yl]thio]-N-(benzothiazol-2-yl)acetamide* (**6m**): Yield 74%, m.p. 214 °C. IR *ν*_max_ (cm^−1^): 3338 (N-H) 3062 (aromatic C–H), 2931 (aliphatic C–H), 1678 (C=O ketone), 1554–1492 (C=N, C=C), 1257–1085 (C-N, C-O). ^1^H-NMR (300 MHz, DMSO-*d*_6_, ppm) δ 1.14–1.31 (5H, m, cyclohexyl-H), 1.52–1.56 (1H, m, cyclohexyl-H), 1.66–1.70 (2H, m, cyclohexyl-H), 1.91–1.95 (2H, m, cyclohexyl-H), 3.26 (2H, t, *J* = 6.4 Hz, SCH_2_CH_2_), 3.39–3.48 (3H, m, SCH_2_CH_2_, cyclohexyl-H), 4.42 (2H, s, COCH_2_), 7.32 (1H, t, *J* = 7.2 Hz, Ar-H), 7.45 (1H, t, *J* = 7.1 Hz, Ar-H), 7.76–7.80 (2H, m, Ar-H, NH), 7.98 (1H, d, *J* = 7.9 Hz, Ar-H), 12.76 (1H, s, NH). ^13^C-NMR (75 MHz, DMSO-*d*_6_, ppm) δ 24.67, 25.66, 26.11, 30.99, 32.46, 36.09, 54.03, 121.16, 122.26, 124.22, 126.69, 131.90, 148.49, 158.11, 163.23, 166.58, 166.82, 169.20. HR-MS (*m*/*z*): [M + H]^+^ calcd for C_21_H_23_N_7_O_2_S_4_: 534.0869; found: 534.0866.

*2-[[5-[2-[[5-(Cyclohexylamino)-1,3,4-thiadiazol-2-yl]thio]ethyl]-1,3,4-oxadiazol-2-yl]thio]-N-(6-methyl benzothiazol-2-yl)acetamide* (**6n**): Yield 77%, m.p. 209 °C. IR *ν*_max_ (cm^−1^): 3336 (N-H) 3026 (aromatic C–H), 2920 (aliphatic C–H), 1674 (C=O ketone), 1585–1467 (C=N, C=C), 1257–1087 (C-N, C-O). ^1^H-NMR (300 MHz, DMSO-*d*_6_, ppm) δ 1.14–1.36 (5H, m, cyclohexyl-H), 1.52–1.56 (1H, m, cyclohexyl-H), 1.66–1.70 (2H, m, cyclohexyl-H), 1.91–1.95 (2H, m, cyclohexyl-H), 2.41 (3H, s, CH_3_), 3.26 (2H, t, *J* = 6.5 Hz, SCH_2_CH_2_), 3.39–3.48 (3H, m, SCH_2_CH_2_, cyclohexyl-H), 4.40 (2H, s, COCH_2_), 7.26 (1H, dd, *J_1_* = 1.4 Hz, *J_2_* = 9.0 Hz, Ar-H), 7.65 (1H, d, *J* = 8.3 Hz, Ar-H), 7.77–7.80 (2H, m, Ar-H, NH), 12.68 (1H, s, NH). ^13^C-NMR (75 MHz, DMSO-*d*_6_, ppm) δ 21.45, 24.67, 25.66, 26.10, 30.99, 32.46, 36.05, 54.02, 120.82, 121.83, 128.02, 132.05, 133.71, 148.49, 157.19, 163.24, 166.57, 169.20. HR-MS (*m*/*z*): [M + H]^+^ calcd for C_22_H_25_N_7_O_2_S_4_: 548.1025; found: 548.1007.

*2-[[5-[2-[[5-(Cyclohexylamino)-1,3,4-thiadiazol-2-yl]thio]ethyl]-1,3,4-oxadiazol-2-yl]thio]-N-(6-methoxy benzothiazol-2-yl)acetamide* (**6o**): Yield 75%, m.p. 198 °C. IR *ν*_max_ (cm^−1^): 3334 (N-H) 3072 (aromatic C–H), 2933 (aliphatic C–H), 1681 (C=O ketone), 1614–1467 (C=N, C=C), 1257–1033 (C-N, C-O). ^1^H-NMR (300 MHz, DMSO-*d*_6_, ppm) δ 1.14–1.36 (5H, m, cyclohexyl-H), 1.52–1.56 (1H, m, cyclohexyl-H), 1.66–1.70 (2H, m, cyclohexyl-H), 1.92–1.95 (2H, m, cyclohexyl-H), 3.26 (2H, t, *J* = 6.5 Hz, SCH_2_CH_2_), 3.39–3.48 (3H, m, SCH_2_CH_2_, cyclohexyl-H), 3.80 (3H, s, OCH_3_), 4.39 (2H, s, COCH_2_), 7.04 (1H, dd, *J_1_* = 2.6 Hz, *J_2_* = 9.0 Hz, Ar-H), 7.57 (1H, d, *J* = 2.5 Hz, Ar-H), 7.66 (1H, d, *J* = 8.1 Hz, Ar-H), 7.79 (1H, d, *J* = 7.3 Hz, NH), 12.62 (1H, s, NH). ^13^C-NMR (75 MHz, DMSO-*d*_6_, ppm) δ 24.67, 25.66, 26.11, 30.99, 32.47, 36.02, 54.03, 56.10, 105.20, 115.53, 121.79, 133.25, 143.03, 148.49, 155.98, 156.72, 163.25, 166.48, 166.56, 169.20. HR-MS (*m*/*z*): [M + H]^+^ calcd for C_22_H_25_N_7_O_3_S_4_: 564.0974; found: 564.0965.

*2-[[5-[2-[[5-(Cyclohexylamino)-1,3,4-thiadiazol-2-yl]thio]ethyl]-1,3,4-oxadiazol-2-yl]thio]-N-(6-chloro benzothiazol-2-yl)acetamide* (**6p**): Yield 74%, m.p. 213 °C. IR *ν*_max_ (cm^−1^): 3334 (N-H) 3088 (aromatic C–H), 2931 (aliphatic C–H), 1681 (C=O ketone), 1612–1467 (C=N, C=C), 1261–1093 (C-N, C-O). ^1^H-NMR (300 MHz, DMSO-*d*_6_, ppm) δ 1.10–1.36 (5H, m, cyclohexyl-H), 1.52–1.56 (1H, m, cyclohexyl-H), 1.65–1.70 (2H, m, cyclohexyl-H), 1.91–1.95 (2H, m, cyclohexyl-H), 3.26 (2H, t, *J* = 6.5 Hz, SCH_2_CH_2_), 3.39–3.49 (3H, m, SCH_2_CH_2_, cyclohexyl-H), 4.41 (2H, s, COCH_2_), 7.47 (1H, dd, *J_1_* = 2.3 Hz, *J_2_* = 9.0 Hz, Ar-H), 7.75–7.80 (2H, m, Ar-H, NH), 8.13 (1H, d, *J* = 2.1 Hz, Ar-H), 12.85 (1H, s, NH). ^13^C-NMR (75 MHz, DMSO-*d*_6_, ppm) δ 24.67, 25.66, 26.10, 30.98, 32.46, 36.03, 54.03, 121.99, 122.39, 127.05, 128.27, 133.61, 147.82, 148.49, 158.96, 163.19, 166.60, 167.05, 169.19. HR-MS (*m*/*z*): [M + H]^+^ calcd for C_21_H_22_ClN_7_O_2_S_4_: 568.0479; found: 568.0469.

*2-[[5-[2-[[5-(Cyclohexylamino)-1,3,4-thiadiazol-2-yl]thio]ethyl]-1,3,4-oxadiazol-2-yl]thio]-N-(6-nitro benzothiazol-2-yl)acetamide* (**6r**): Yield 68%, m.p. 205 °C. IR *ν*_max_ (cm^−1^): 3338 (N-H) 3051 (aromatic C–H), 2933 (aliphatic C–H), 1687 (C=O ketone), 1614–1446 (C=N, C=C), 1514 and 1334 (NO_2_), 1288–1093 (C-N, C-O). ^1^H-NMR (300 MHz, DMSO-*d*_6_, ppm) δ 1.13–1.34 (5H, m, cyclohexyl-H), 1.52–1.56 (1H, m, cyclohexyl-H), 1.65–1.69 (2H, m, cyclohexyl-H), 1.91–1.94 (2H, m, cyclohexyl-H), 3.26 (2H, t, *J* = 6.5 Hz, SCH_2_CH_2_), 3.39–3.48 (3H, m, SCH_2_CH_2_, cyclohexyl-H), 4.45 (2H, s, COCH_2_), 7.78 (1H, d, *J* = 7.3 Hz, NH), 7.92 (1H, d, *J* = 9.0 Hz, Ar-H), 8.29 (1H, dd, *J_1_* = 2.4 Hz, *J_2_* = 8.9 Hz, Ar-H), 9.06 (1H, d, *J* = 2.3 Hz, Ar-H), 13.17 (1H, s, NH). ^13^C-NMR (75 MHz, DMSO-*d*_6_, ppm) δ 24.67, 25.66, 26.09, 30.97, 32.45, 36.09, 54.02, 119.61, 121.28, 122.30, 132.67, 143.59, 148.48, 153.82, 163.14, 163.65, 166.64, 167.65, 169.17. HR-MS (*m*/*z*): [M + H]^+^ calcd for C_21_H_22_N_8_O_4_S_4_: 579.0720; found: 579.0697.

*2-[[5-[2-[[5-(Cyclohexylamino)-1,3,4-thiadiazol-2-yl]thio]ethyl]-1,3,4-oxadiazol-2-yl]thio]-N-(6-fluoro benzothiazol-2-yl)acetamide* (**6s**): Yield 67%, m.p. 218 °C. IR *ν*_max_ (cm^−1^): 3338 (N-H) 3072 (aromatic C–H), 2927 (aliphatic C–H), 1674 (C=O ketone), 1562–1458 (C = N, C = C), 1147–1049 (C-N, C-O). ^1^H-NMR (300 MHz, DMSO-*d*_6_, ppm) δ 1.14–1.36 (5H, m, cyclohexyl-H), 1.52–1.56 (1H, m, cyclohexyl-H), 1.66–1.70 (2H, m, cyclohexyl-H), 1.91–1.95 (2H, m, cyclohexyl-H), 3.26 (2H, t, *J* = 6.5 Hz, SCH_2_CH_2_), 3.39–3.52 (3H, m, SCH_2_CH_2_, cyclohexyl-H), 4.41 (2H, s, COCH_2_), 7.26–7.33 (1H, m, Ar-H), 7.76–7.80 (2H, m, Ar-H, NH), 7.91 (1H, dd, *J_1_* = 2.7 Hz, *J_2_* = 9.0 Hz, Ar-H), 12.78 (1H, s, NH). ^13^C-NMR (75 MHz, DMSO-*d*_6_, ppm) δ 24.67, 25.66, 26.10, 30.98, 32.46, 36.01, 54.03, 108.72 (d, *J* = 27.0 Hz), 114.83 (d, *J* = 24.0 Hz), 122.32 (d, *J* = 9.8 Hz), 133.17 (d, *J* = 11.3 Hz), 145.66, 148.49, 158.10 (d, *J* = 2.2 Hz), 159.20 (d, *J* = 239.3 Hz), 163.21, 166.59, 166.92, 169.19. HR-MS (*m*/*z*): [M + H]^+^ calcd for C_21_H_22_FN_7_O_2_S_4_: 552.0775; found: 552.0751.

### 3.2. Antifungal Activity Assay

The anticandidal effect of compounds **6a**–**6s** were screened in accord with the EUCAST definitive (EDef 7.1) method [25]. Ketoconazole was employed as a reference drug. The in vitro growth inhibitory activity of the compounds were tested against *C. albicans* (ATCC 24433), *C. glabrata* (ATCC 90030), *C. krusei* (ATCC 6258) *and C. parapsilosis* (ATCC 22019)*.* The results were obtained as MIC values and MIC readings were accomplished twice for each chemical agent. After incubating overnight at 37 °C, the fungal strains were sustained in Roswell Park Memorial Institute (RPMI) medium.

Adjustment of the inoculum density to a 0.5 McFarland turbidity standard was carried out by using a spectrophotometer. The resulting inoculum suspension contained 0.5 × 10^5^–2.5 × 10^5^ cfu/mL for fungi. The two-fold serial dilutions technique were applied and the test was carried out in Mueller–Hinton broth and RPMI at pH7 and the test was performed for medium at pH7. The last well on the microplates containing only inoculated broth and the last well with no growth of microorganism represent controls and the MIC_50_ in μg/mL, respectively. DMSO was used as solvent. Further dilutions of the compounds and standard drugs in the RPMI medium were prepared in the required quantities at contcentrations of 800, 400, 200, 100, 50, 25, 12.5, 6.25, 3.125, 1.5625 and 0.78 µg/mL. After a 24 h incubation of the completed plates, resazurin (20 µg/mL) was added into each well in order to observe the fungal growth. At the end of a 2 h incubation period of the final plates including microorganism strains, MIC_50_ values were determined with a microplate reader at 590 nm excitation and 560 nm emission. Each experiment in antifungal assay was performed twice. 

### 3.3. Effect of Compounds **6e**, **6k**, and **6s** on Ergosterol Content of C. krusei

The ergosterol level was determined using the method described by Breivik and Owades [26] with adjustments according to [2]. *C. krusei* was inoculated with Sabouraud dextrose broth (Difco) supplemented with compounds **6e**, **6k**, **6r** and the positive control, ketoconazole. The cultures were incubated, and the stationary-phase cells were harvested. After the addition of 25% alcoholic potassium hydroxide solution, cell suspensions were separated and incubated. Sterols were extracted by sterile distilled water and chloroform, and then the extract was injected into the LC-MS-MS system (Shimadzu LCMS 8040, Kyoto, Japan). The ergosterol quantity in the negative control samples was considered as 100%. All concentrations were analysed in quadruplicate, and the results were presented as mean ± standard deviation (SD).

### 3.4. Cytotoxicity Test

The MTT assay was carried out to determine the cytotoxicity of the synthesized compounds as previously reported in [21]. NIH/3T3 mouse embryonic fibroblast cells (ATCC^®^ CRL-1658™, London, UK) were incubated according to the supplier’s references and seeded at 1 × 10^4^ cells into each well of 96-well plates. After this process, cells were treated with the compounds at concentrations ranging from 800 µg/mL to 0.78 µg/mL. By means of the following formula, percent inhibition was evaluated for each concentration. The IC_50_ values were determined by using dose-response curves plotted against compound concentrations applied [27].

% Inhibition = 100 − (Mean Sample × 100/Mean Solvent)

### 3.5. Molecular Docking Studies

The crystal structures of 14 alpha-sterol demethylase enzyme (PDB ID: 1EA1) [22] that was crystallized with fluconazole, was retrieved from the Protein Data Bank server (www.pdb.org). The structure of ligand was built using the Schrödinger Maestro [28] interface and then was submitted to the Protein Preparation Wizard protocol of the Schrödinger Suite 2016, Update 2 [29]. The ligand was prepared by the LigPrep 3.8 [30] to correctly assign the protonation states at pH 7.4 ± 1.0 and the atom types. Bond orders were designated and hydrogen atoms were added to the structures. The grid generation was formed using the Glide 7.1 [31] program and docking runs were performed with single precision docking mode (SP). 

## 4. Conclusions

In conclusion, the eighteen newly synthesized 2-[[5-[2-[[5-(cyclohexylamino)-1,3,4-thiadiazol-2-yl]thio]ethyl]-1,3,4-oxadiazol-2-yl]thio]-1-substituted ethan-1-one derivatives (**6a**–**6s**) were tested for their inhibitory activity against the growth of four *Candida* species, and three of them (**6e**, **6k** and **6r**) exhibited comparable antifungal activity to that of reference drug ketoconazole. After preliminary studies, ergosterol level quantification assay and docking studies provided insight into the mechanism of their antifungal activity. It is therefore likely that such connections exist between their mechanism of action and the inhibition of ergosterol biosynthesis. Consequently, all experiments indicated that this kind of structures may help to improve and/or design new antifungals due to their high activity and nontoxic nature.

## Figures and Tables

**Table:** 

R/Ar	Compound	R’	Compound	R’	Compound	R’
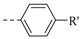	**6a**	H	**6b**	CH_3_	**6c**	OCH_3_
	**6d**	Cl	**6e**	NO_2_	**6f**	F
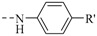	**6g**	H	**6h**	CH_3_	**6i**	OCH_3_
	**6j**	Cl	**6k**	NO_2_	**6l**	F
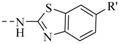	**6m**	H	**6n**	CH_3_	**6o**	OCH_3_
	**6p**	Cl	**6r**	NO_2_	**6s**	F

**Table 1:** Minimum inhibitory concentration (MIC_50_; µg/mL) values of compounds on four strains of Candida (**6a**–**6s**).

Compound	*C. albicans*	*C. glabrata*	*C. krusei*	*C. parapsilopsis*
**6a**	25	50	50	50
**6b**	50	50	100	50
**6c**	100	25	100	50
**6d**	100	25	50	50
**6e**	1.56	1.56	0.78	0.78
**6f**	50	50	25	50
**6g**	50	50	50	50
**6h**	25	100	100	50
**6i**	25	100	100	50
**6j**	25	50	50	50
**6k**	1.56	1.56	1.56	1.56
**6l**	100	50	50	25
**6m**	50	50	50	25
**6n**	100	25	50	25
**6o**	100	25	25	50
**6p**	50	25	25	50
**6r**	3.12	3.12	1.56	1.56
**6s**	50	6.25	50	50
**Ketoconazole**	0.78	1.56	1.56	1.56

**Table 2:** Cytotoxic activity and ergosterol biosynthesis potency of the compounds **6e, 6k** and **6r** against NIH/3T3 cell line and *C. krusei*, respectively.

Comp.	IC_50_ (µg/mL)	Inhibition of Ergosterol Biosynthesis (%)		
		0.78 µg/mL	1.56 µg/mL	3.12 µg/mL
**6e**	>500	76.45 ± 3.67	82.94 ± 4.72	88.56 ± 4.52
**6k**	>500	58.72 ± 2.41	74.65 ± 3.26	81.29 ± 4.19
**6r**	406.066 ± 9.36	61.43 ± 1.88	70.59 ± 4.23	80.21 ± 3.70
**Ketoconazole**	-	60.99 ± 2.94	73.12 ± 4.16	84.56 ± 3.01

## Supplementary Materials

The spectra of all compounds are available online.
