# Supplementary material for: Synthesis of Oxadiazole-Thiadiazole Hybrids and Their Anticandidal Activity
Source: Molecules. 2017 Nov 18;22(11):2004. doi: 10.3390/molecules22112004 (PMC6150172; doi:10.3390/molecules22112004)
Supplement: Supplementary file 1 [file molecules-22-02004-s001.pdf]

Supporting Information

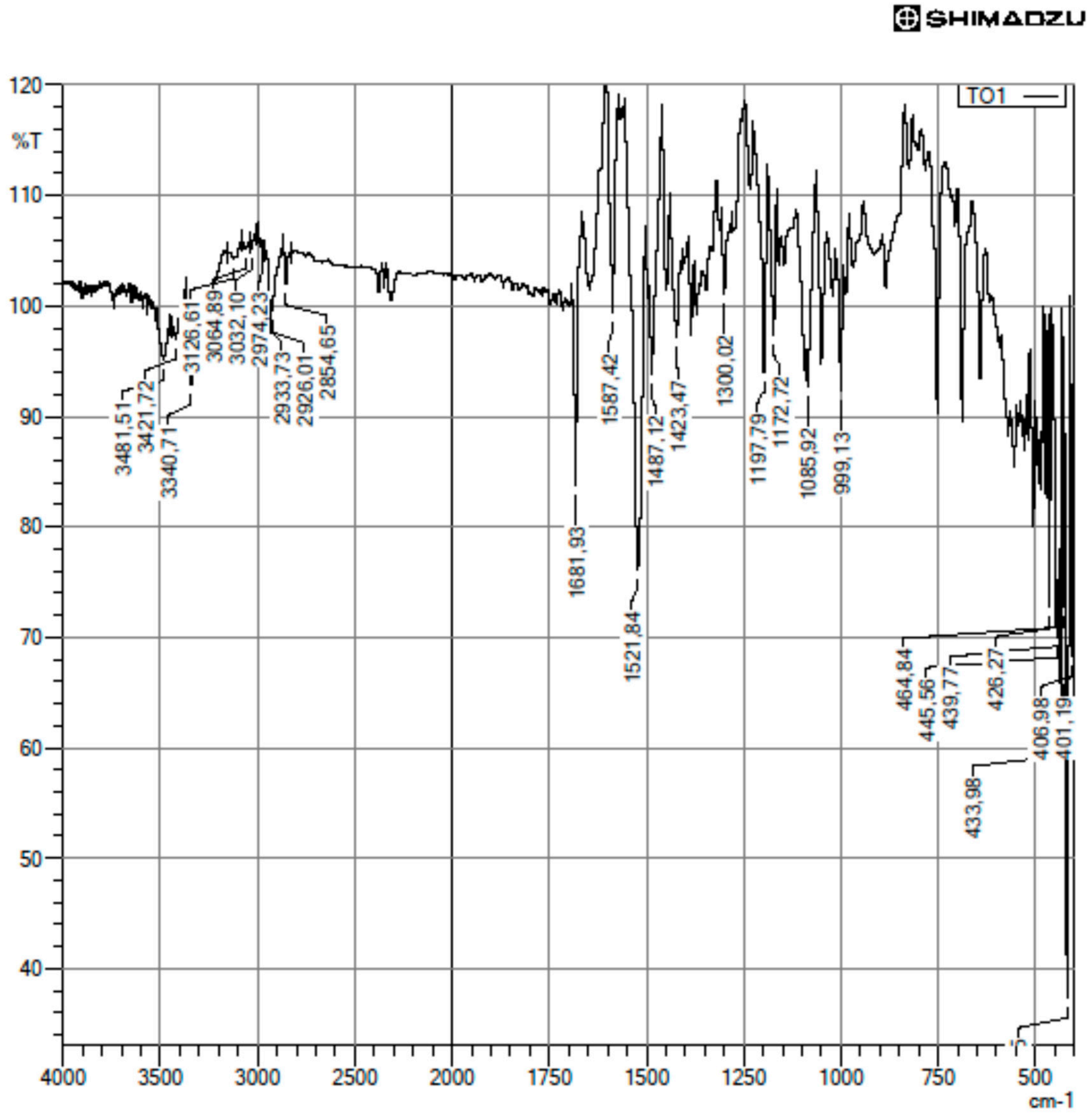

C:¥Users¥dopnalab¥Desktop¥BET7L¥TO¥TO1.ispd

	Item	Value
2	Sample name	TO-1
3	Sample ID	
4	Option	
5	Intensity Mode	%Transmittance
6	Apodization	Happ-Genzel
9	No. of Scans	10

Figure 1. Compound 6a IR spectrum

Formula Predictor Report - TO1_1.lcd

Data File: C:\LabSolutions\Data\Analiz\BKaya\TO1_1.lcd

Elmt	Val.	Min	Max	Elmt	Val.	Min	Max	Elmt	Val.	Min	Max	Use Adduct
H	1	10	40	O	2	2	5	Cl	1	0	0	H
C	4	10	26	F	1	0	0	Br	1	0	0	
N	3	5	5	S	2	3	3					

Error Margin (ppm): 5
HC Ratio: unlimited
Max Isotopes: 3
MSn Iso RI (%): 10.00

DBE Range: 0.0 - 19.0
Apply N Rule: yes
Isotope RI (%): 1.00
MSn Logic Mode: AND

Electron Ions: both
Use MSn Info: no
Isotope Res: 10000
Max Results: 500

Event#: 1 MS(E+) Ret. Time : 7.973 -> 8.080 Scan# : 1197 -> 1213

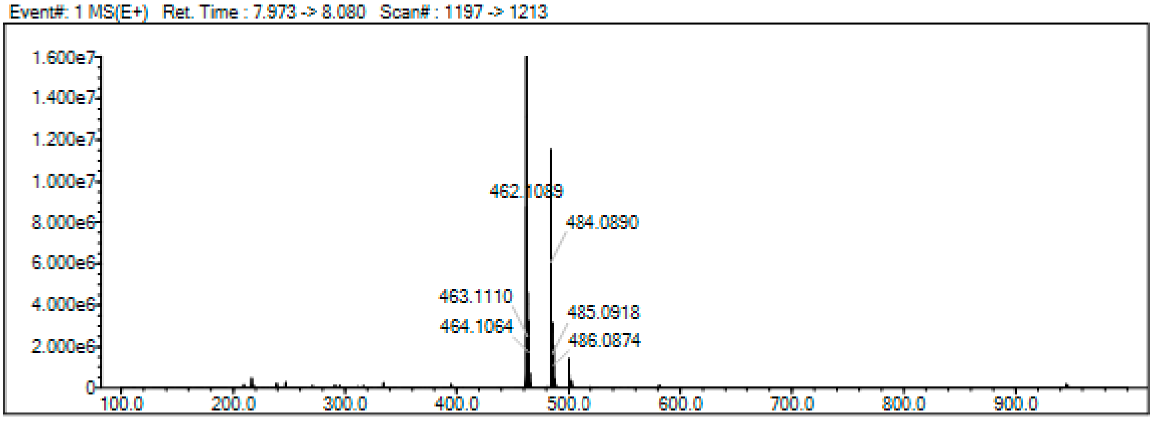

Measured region for 462.1089 m/z

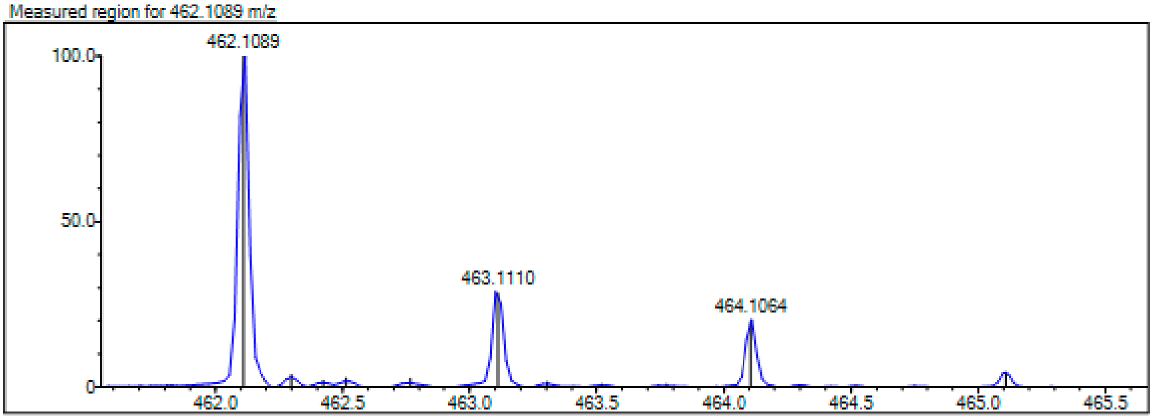

C20 H23 N5 O2 S3 [M+H]+ : Predicted region for 462.1087 m/z

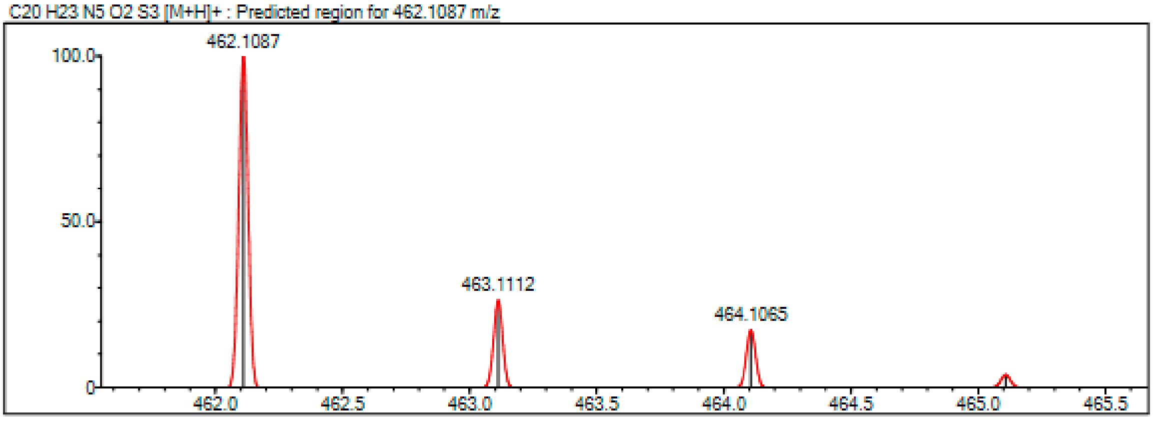

Rank	Score	Formula (M)	Ion	Meas. m/z	Pred. m/z	Df. (mDa)	Df. (ppm)	Iso	DBE
1	91.98	C20 H23 N5 O2 S3	[M+H]+	462.1089	462.1087	0.2	0.43	91.98	12.0

Figure 2. Compound 6a Mass spectrum

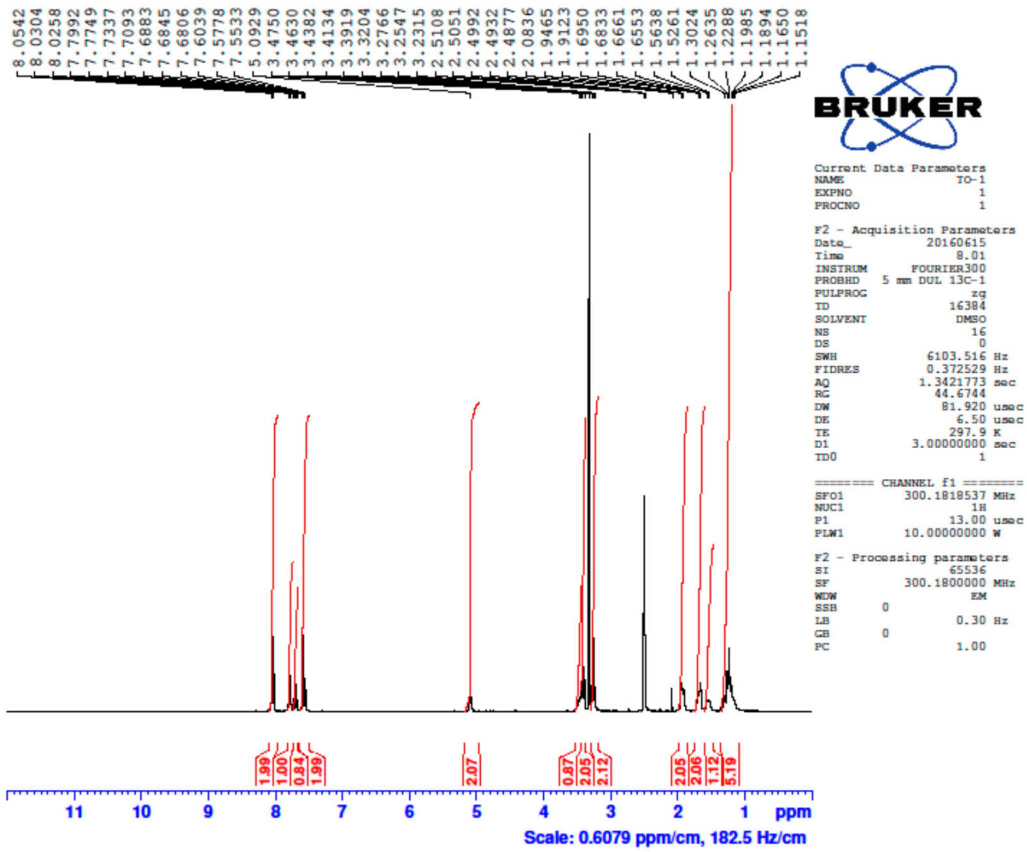

Figure 3. Compound 6a ^{1}H NMR spectrum

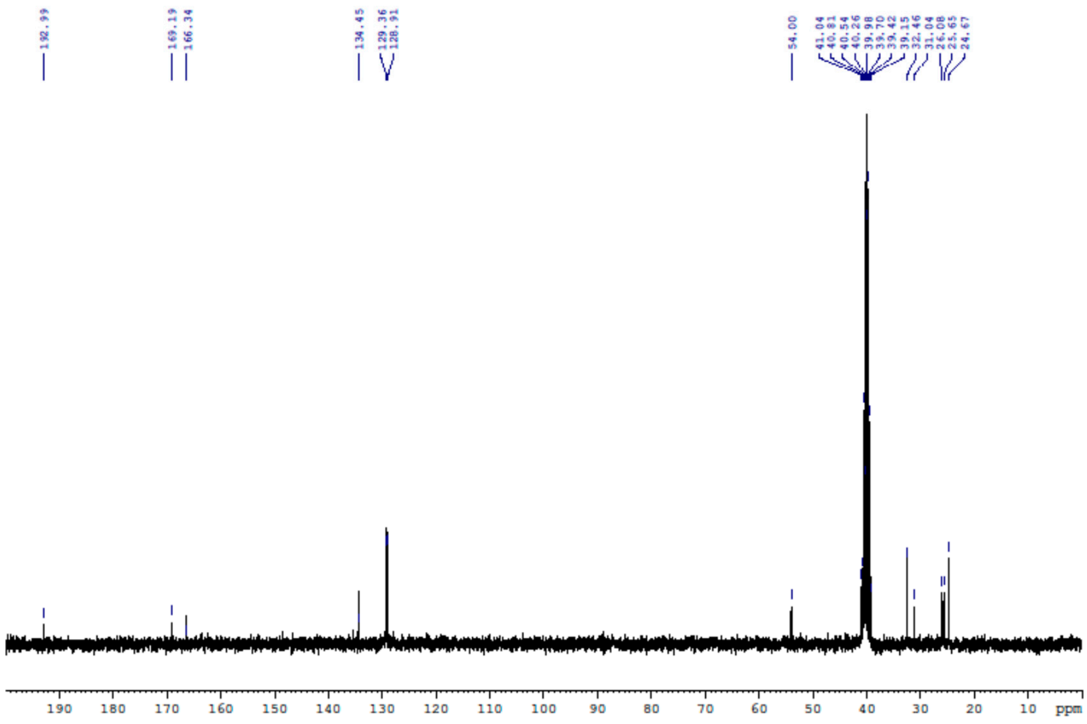

Figure 4. Compound 6a ^{13}C NMR spectrum

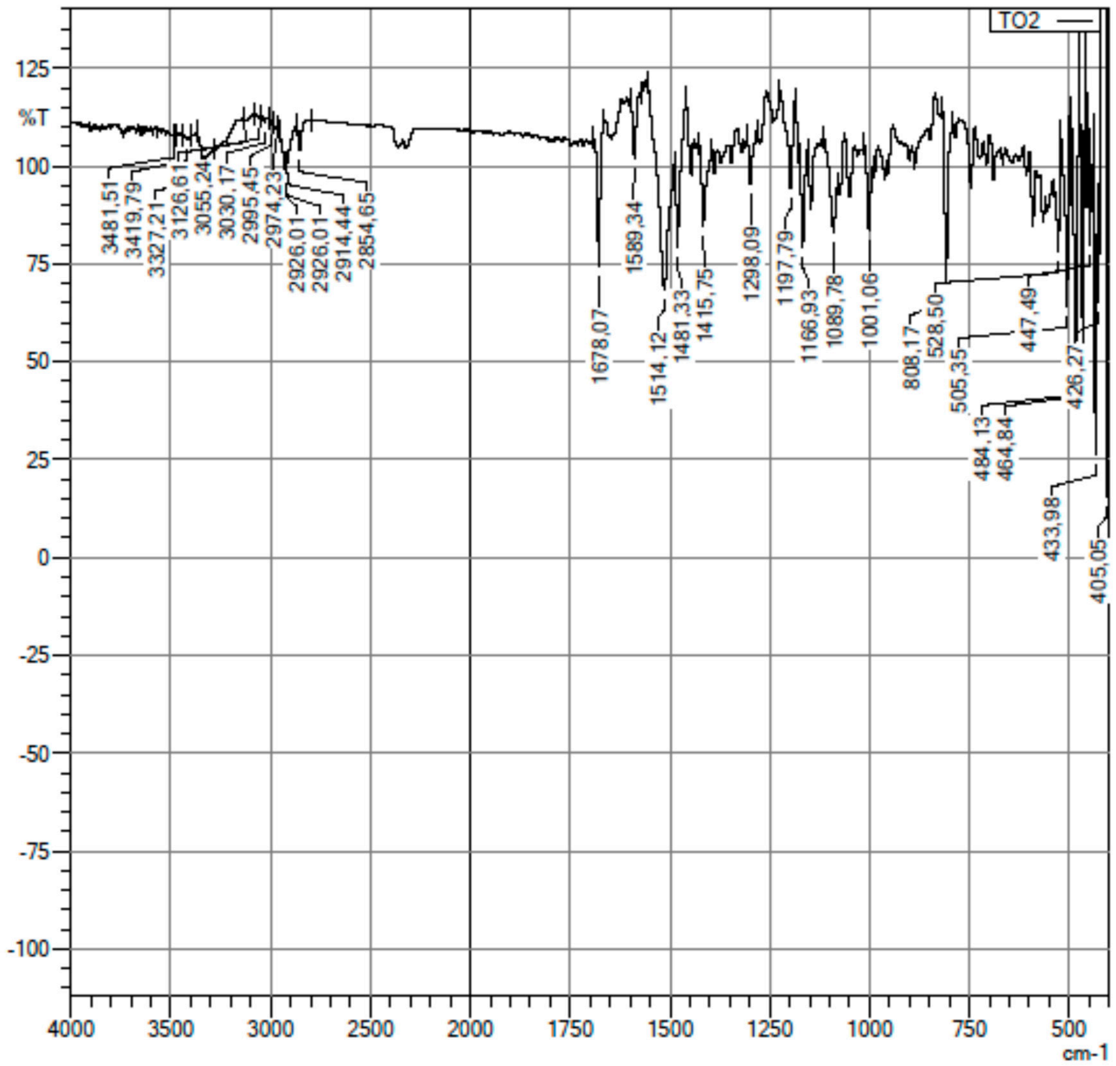

C:¥Users¥dopnalab¥Desktop¥BET7L¥TO¥TO2.ispd

	Item	Value
2	Sample name	TO-2
3	Sample ID	
4	Option	
5	Intensity Mode	%Transmittance
6	Apodization	Happ-Genzel
9	No. of Scans	10

Figure 5. Compound 6b IR spectrum

Formula Predictor Report - TO2_2.lcd Page 1 of 1

Data File: C:\LabSolutions\Data\Analiz\BKaya\TO2_2.lcd

Elmt	Val.	Min	Max	Elmt	Val.	Min	Max	Elmt	Val.	Min	Max	Use Adduct
H	1	10	40	O	2	2	5	Cl	1	0	0	H
C	4	10	26	F	1	0	0	Br	1	0	0	
N	3	5	5	S	2	3	3					

Error Margin (ppm): 5
HC Ratio: unlimited
Max Isotopes: 3
MSn Iso RI (%): 10.00

DBE Range: 0.0 - 19.0
Apply N Rule: yes
Isotope RI (%): 1.00
MSn Logic Mode: AND

Electron Ions: both
Use MSn Info: no
Isotope Res: 10000
Max Results: 500

Event#: 1 MS(E+) Ret. Time : 8.000 -> 8.080 Scan# : 1201 -> 1213

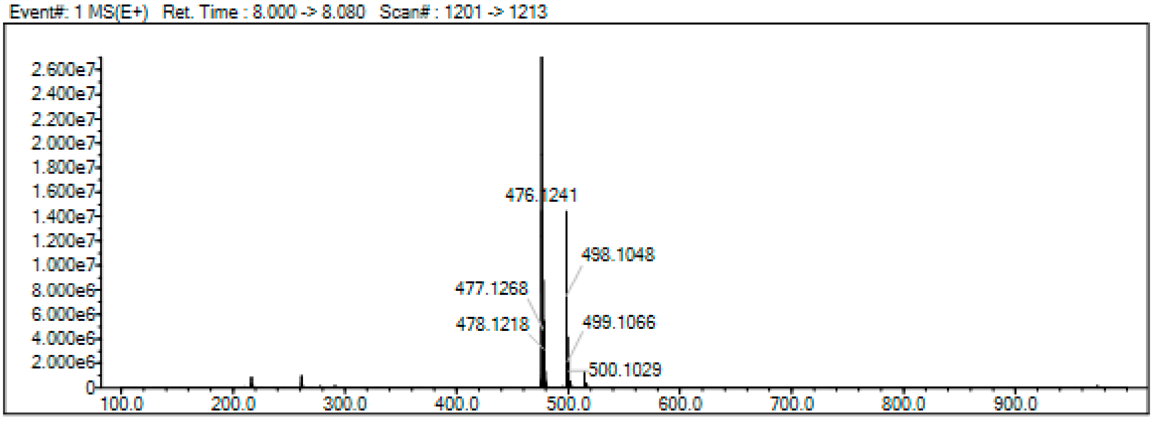

Measured region for 476.1241 m/z

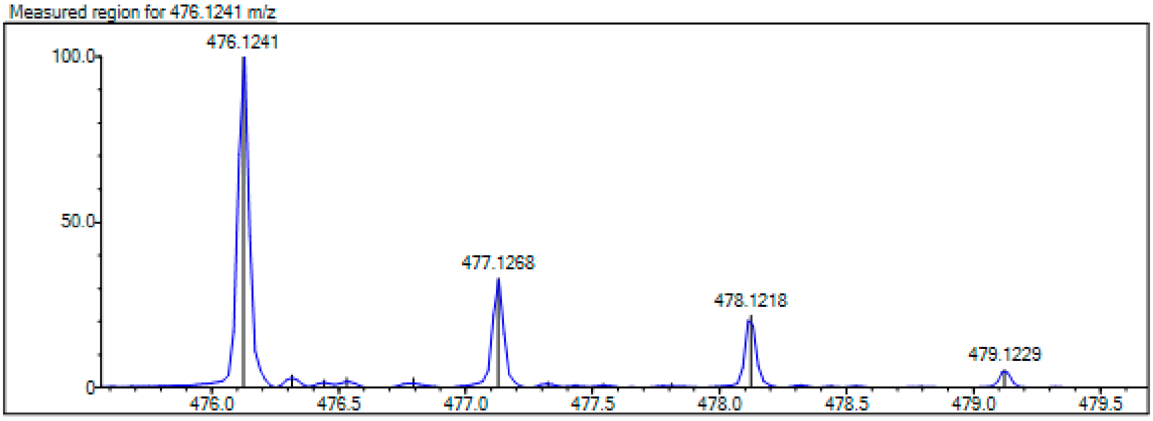

C21 H25 N5 O2 S3 [M+H]+ : Predicted region for 476.1243 m/z

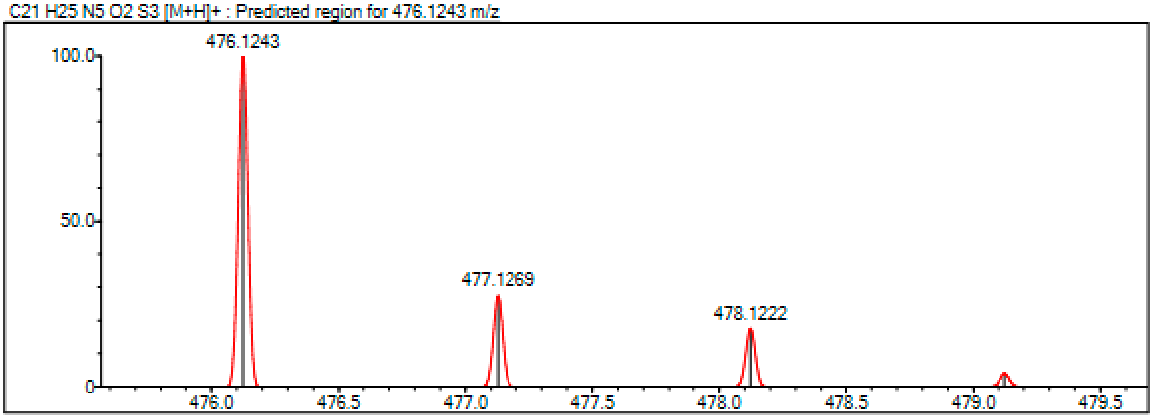

Rank	Score	Formula (M)	Ion	Meas. m/z	Pred. m/z	Df. (mDa)	Df. (ppm)	Iso	DBE
1	89.12	C21 H25 N5 O2 S3	[M+H]+	476.1241	476.1243	-0.2	-0.42	89.12	12.0

Figure 6. Compound 6b Mass spectrum

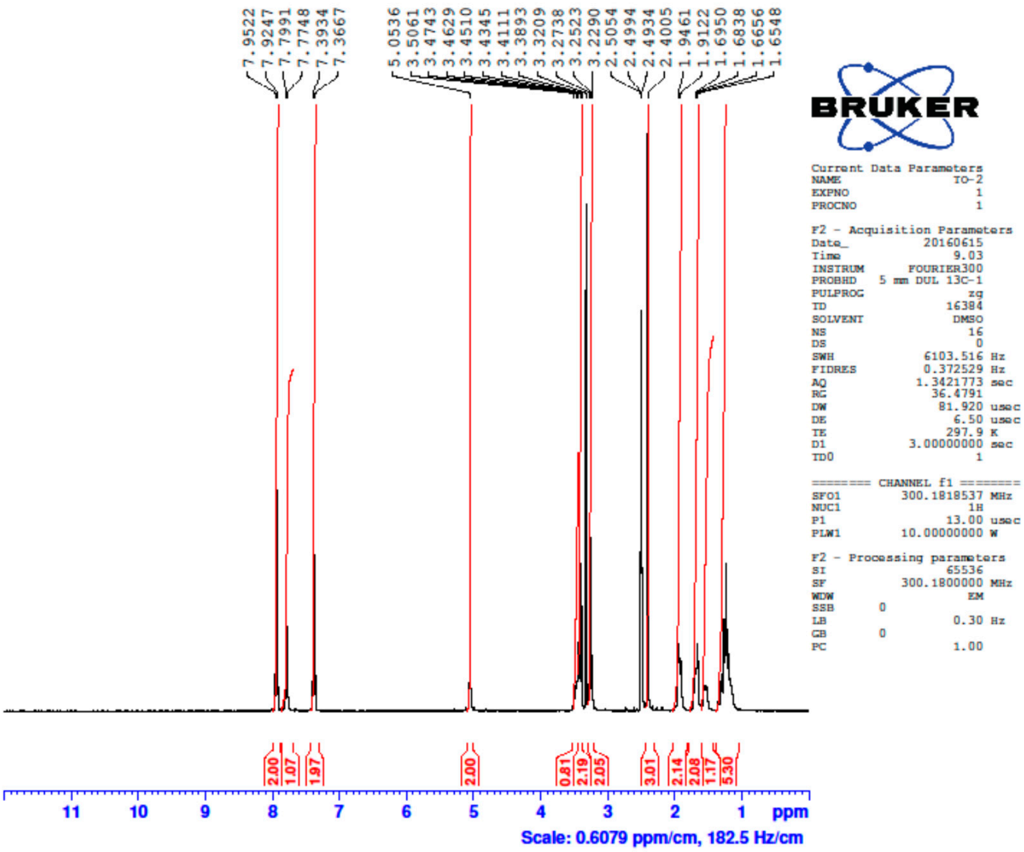

Figure 7. Compound 6b ^{1}H NMR spectrum

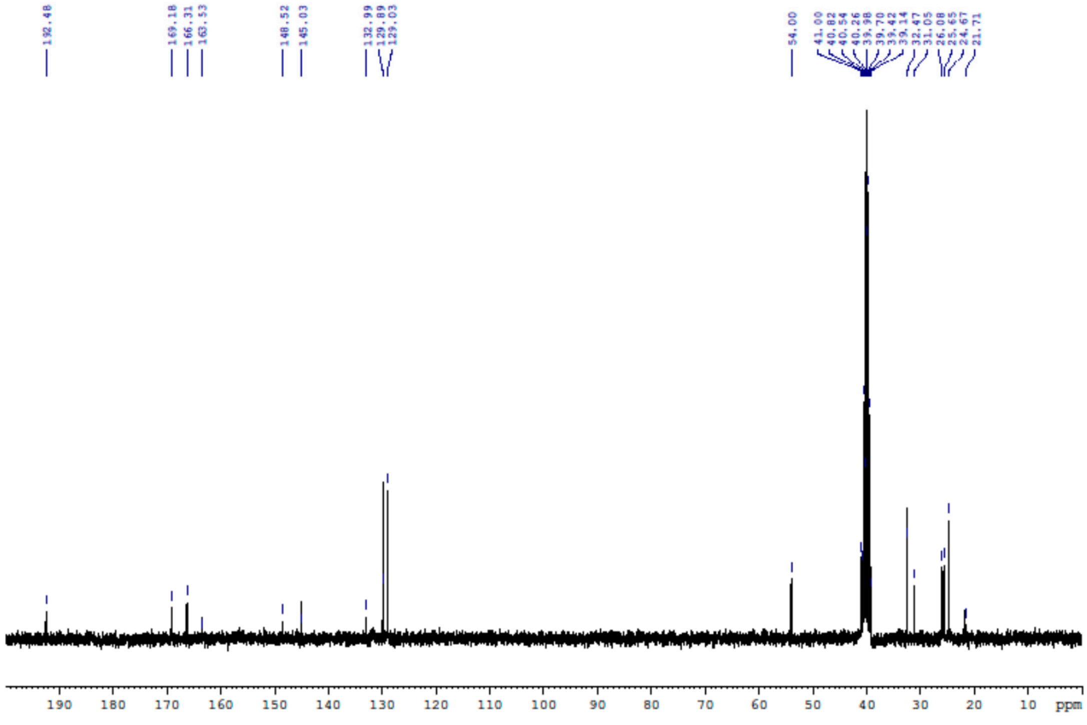

Figure 8. Compound 6b ^{13}C NMR spectrum

SHIMADZU

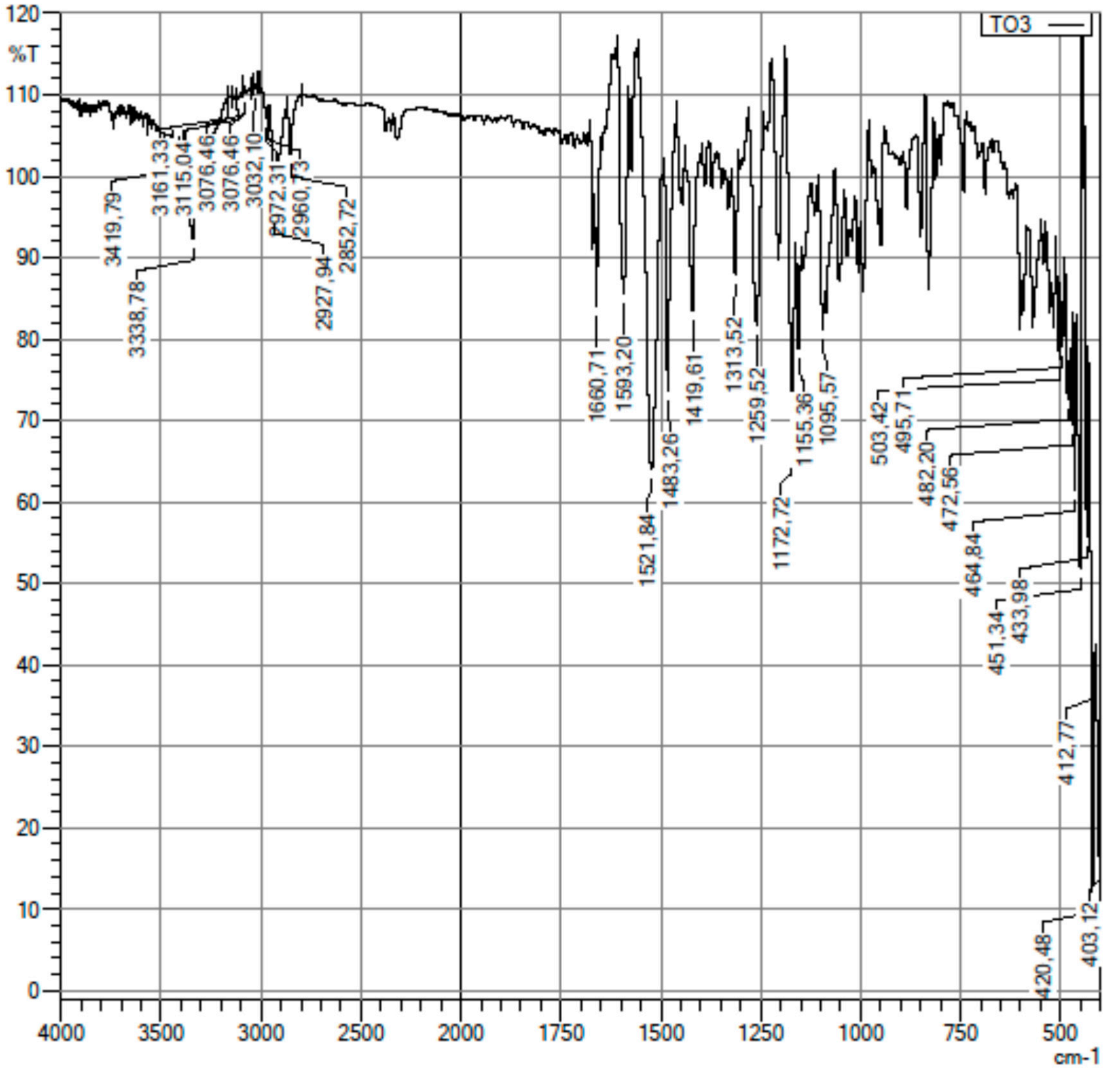

C:¥Users¥dopnalab¥Desktop¥BET7L¥TO¥TO3.ispd

	Item	Value
2	Sample name	TO-3
3	Sample ID	
4	Option	
5	Intensity Mode	%Transmittance
6	Apodization	Happ-Genzel
9	No. of Scans	10

1

Figure 9. Compound 6c IR spectrum

Data File: C:\LabSolutions\Data\Analiz\BKaya\TO3-1_3.lcd

Elmt	Val.	Min	Max	Elmt	Val.	Min	Max	Elmt	Val.	Min	Max	Use Adduct
H	1	10	40	O	2	2	5	Cl	1	0	0	H
C	4	10	26	F	1	0	0	Br	1	0	0	
N	3	5	5	S	2	3	3					

Error Margin (ppm):	5	DBE Range:	0.0 - 19.0	Electron Ions:	both
HC Ratio:	unlimited	Apply N Rule:	yes	Use MSn Info:	no
Max Isotopes:	3	Isotope RI (%):	1.00	Isotope Res:	10000
MSn Iso RI (%):	10.00	MSn Logic Mode:	AND	Max Results:	500

Event#: 1 MS(E+) Ret. Time : 7.800 -> 7.853 Scan# : 1171 -> 1179

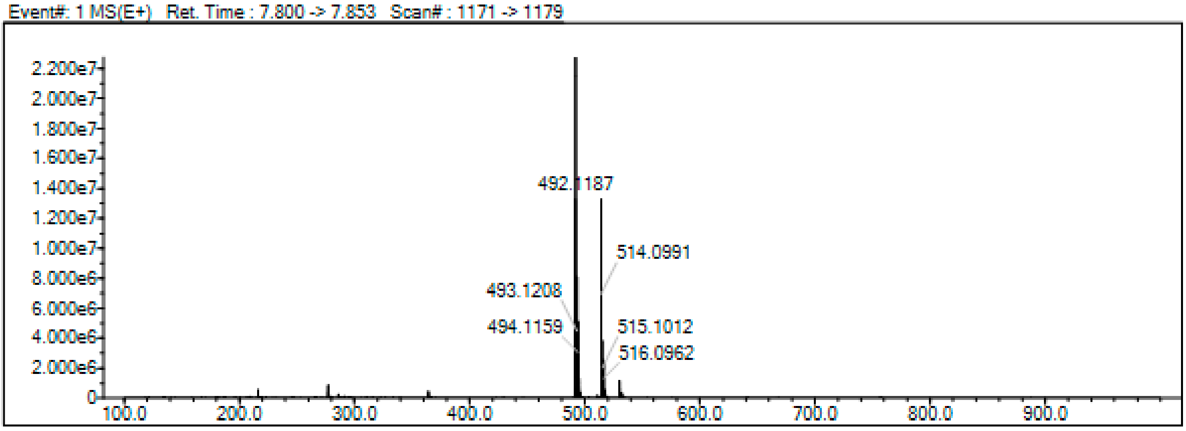

Measured region for 492.1187 m/z

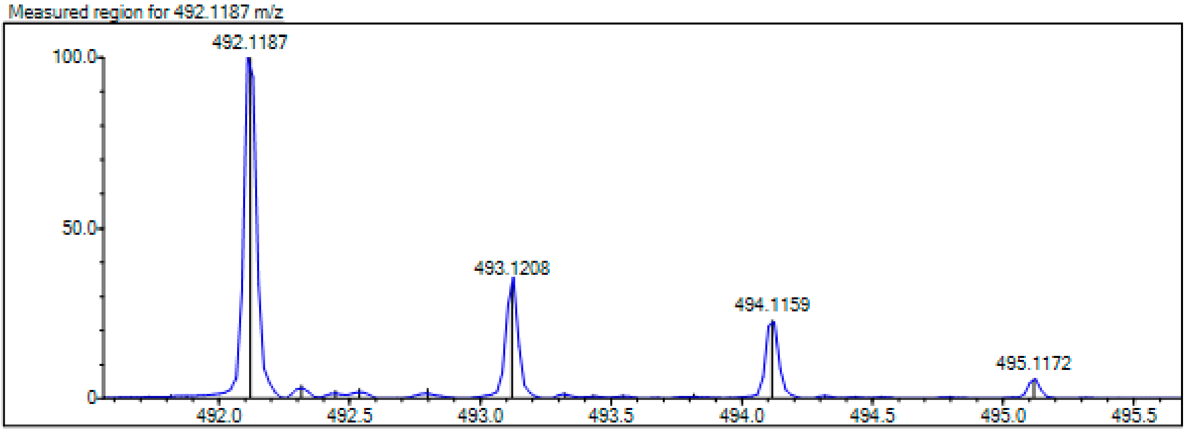

C21 H25 N5 O3 S3 [M+H]+ : Predicted region for 492.1192 m/z

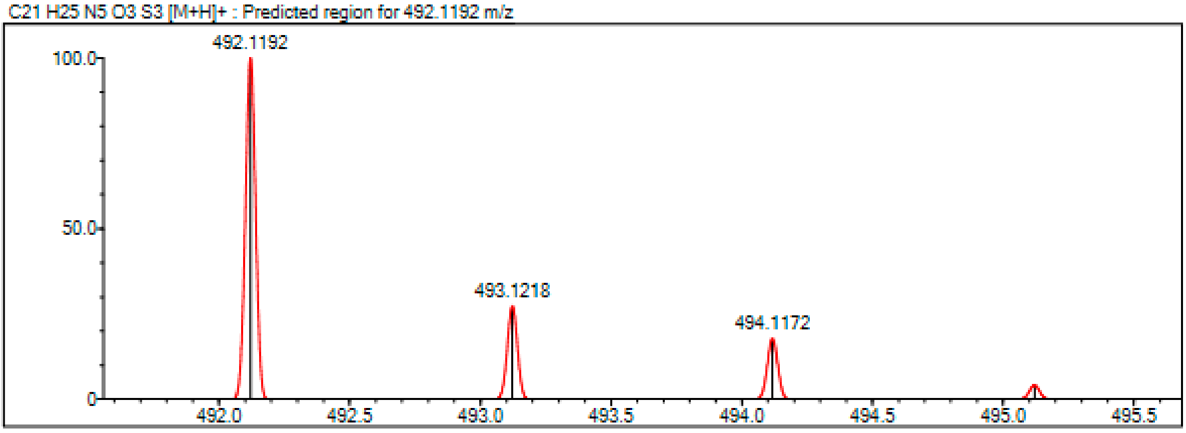

Rank	Score	Formula (M)	Ion	Meas. m/z	Pred. m/z	Df. (mDa)	Df. (ppm)	Iso	DBE
1	83.33	C21 H25 N5 O3 S3	[M+H]+	492.1187	492.1192	-0.5	-1.02	83.37	12.0

Figure 10. Compound 6c Mass spectrum

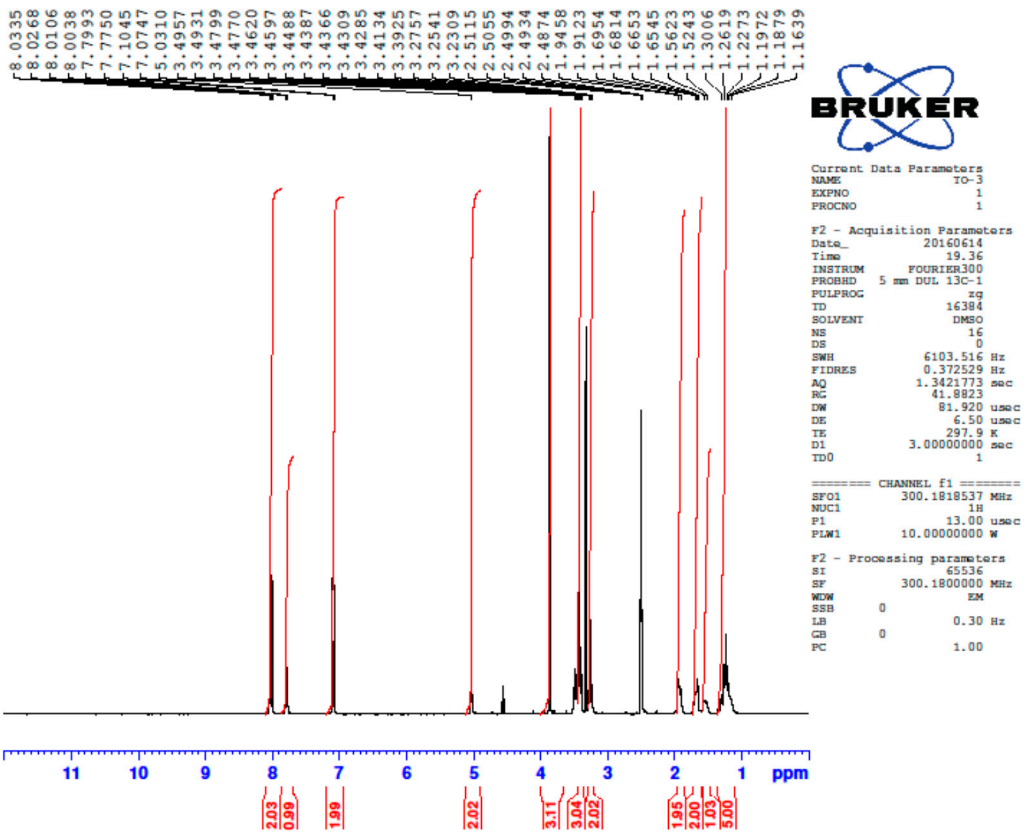

Figure 11. Compound 6c ^{1}H NMR spectrum

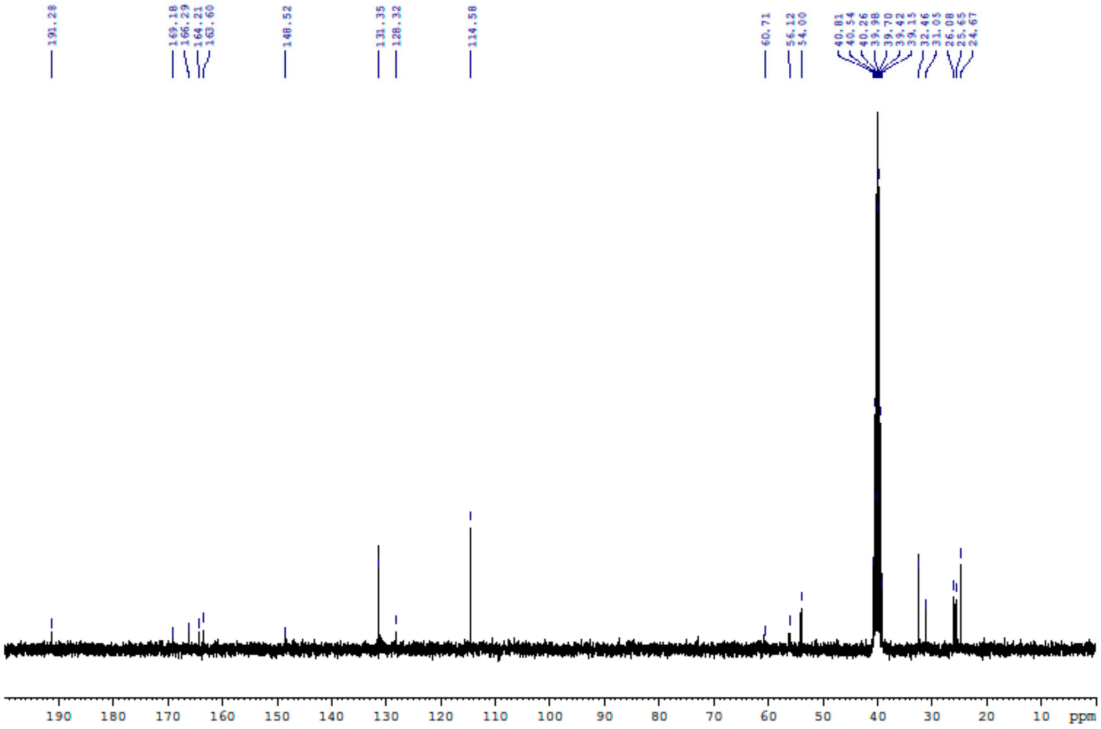

Figure 12. Compound 6c ^{13}C NMR spectrum

SHIMADZU

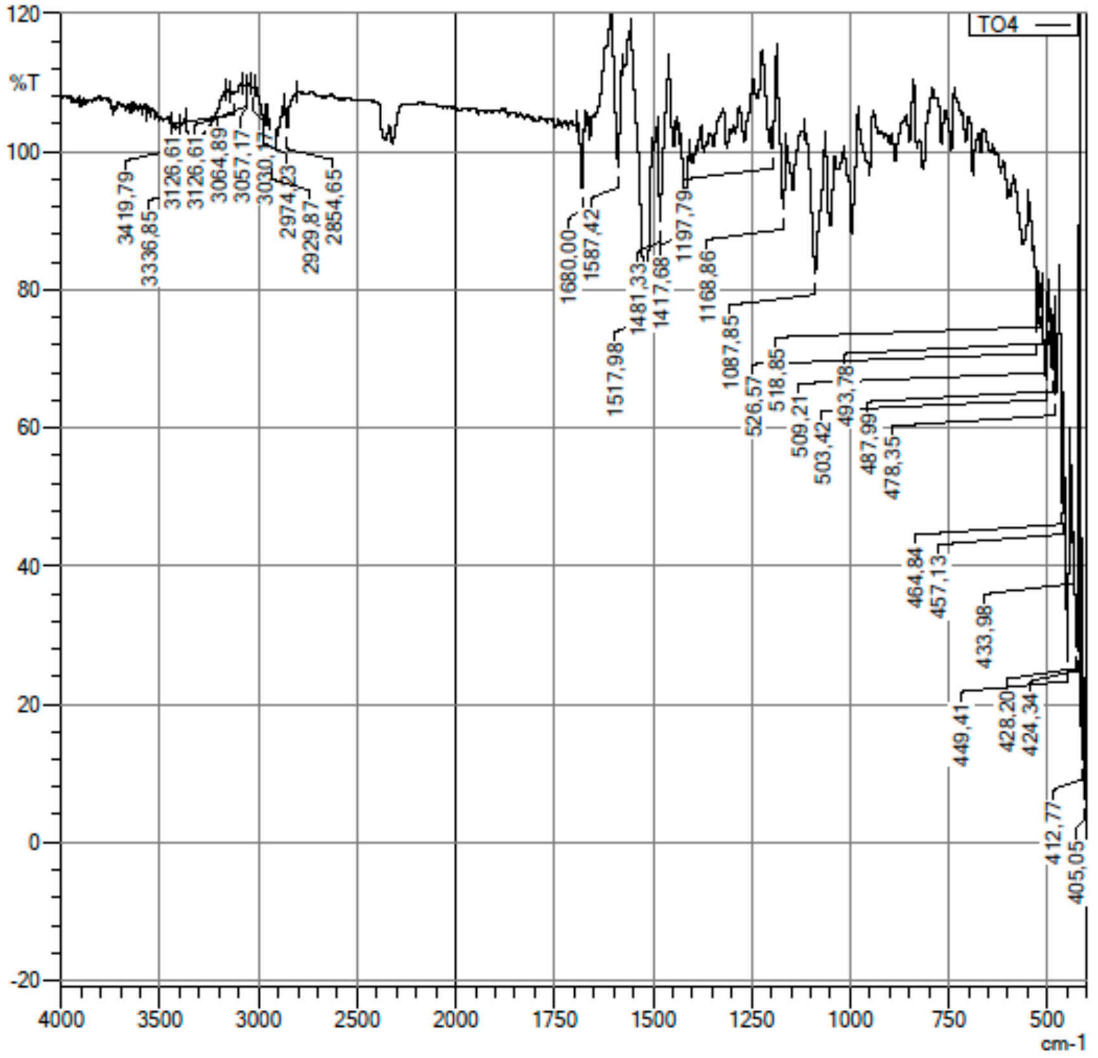

C:¥Users¥dopnalab¥Desktop¥BETアL¥TO¥TO4.ispd

	Item	Value
2	Sample name	TO-4
3	Sample ID	
4	Option	
5	Intensity Mode	%Transmittance
6	Apodization	Happ-Genzel
9	No. of Scans	10

1

Figure 13. Compound 6d IR spectrum

Data File: C:\LabSolutions\Data\Analiz\BKaya\TO4-1_5.lcd

Elmt	Val.	Min	Max	Elmt	Val.	Min	Max	Elmt	Val.	Min	Max	Use Adduct
H	1	10	40	O	2	2	5	Cl	1	1	1	H
C	4	10	26	F	1	0	0	Br	1	0	0	
N	3	5	5	S	2	3	3					

Error Margin (ppm):	5	DBE Range:	0.0 - 19.0	Electron Ions:	both
HC Ratio:	unlimited	Apply N Rule:	yes	Use MSn Info:	no
Max Isotopes:	3	Isotope RI (%):	1.00	Isotope Res:	10000
MSn Iso RI (%):	10.00	MSn Logic Mode:	AND	Max Results:	500

Event#: 1 MS(E+) Ret. Time : 8.147 -> 8.227 Scan# : 1223 -> 1235

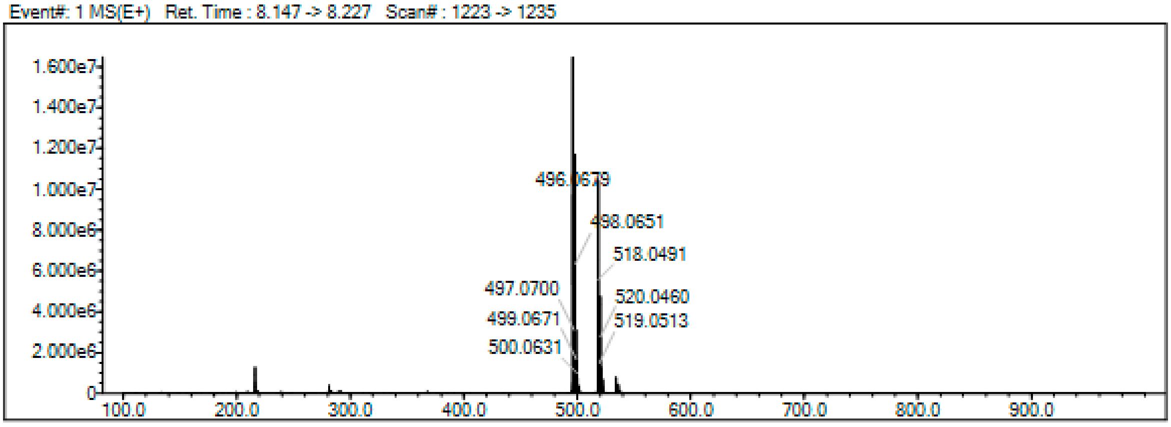

Measured region for 496.0679 m/z

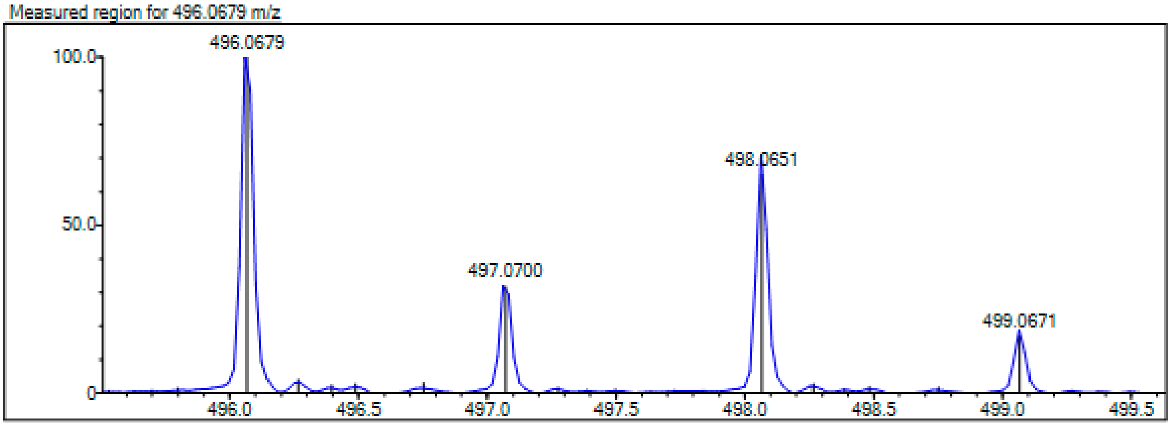

C20 H22 N5 O2 S3 Cl [M+H]+ : Predicted region for 496.0697 m/z

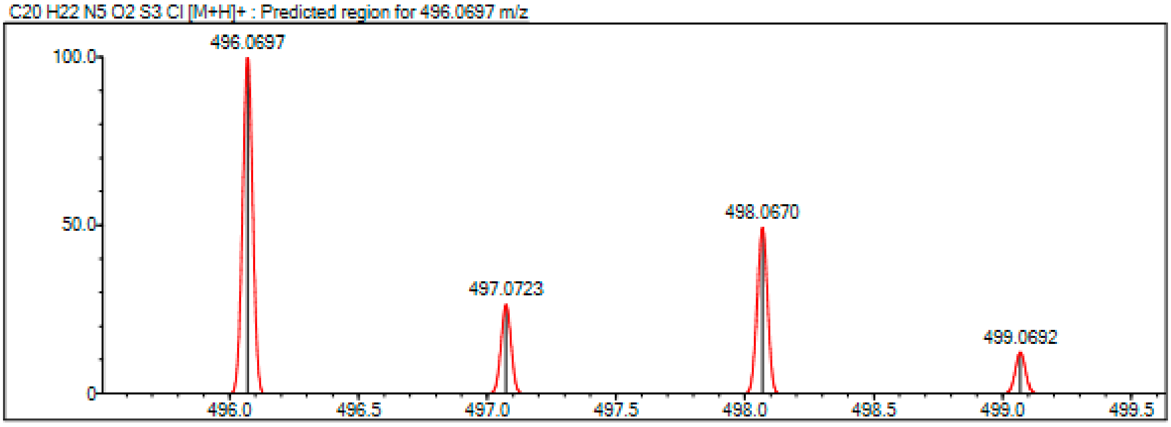

Rank	Score	Formula (M)	Ion	Meas. m/z	Pred. m/z	Df. (mDa)	Df. (ppm)	Iso	DBE
1	61.33	C20 H22 N5 O2 S3 Cl	[M+H]+	496.0679	496.0697	-1.8	-3.63	65.65	12.0

Figure 14. Compound 6d Mass spectrum

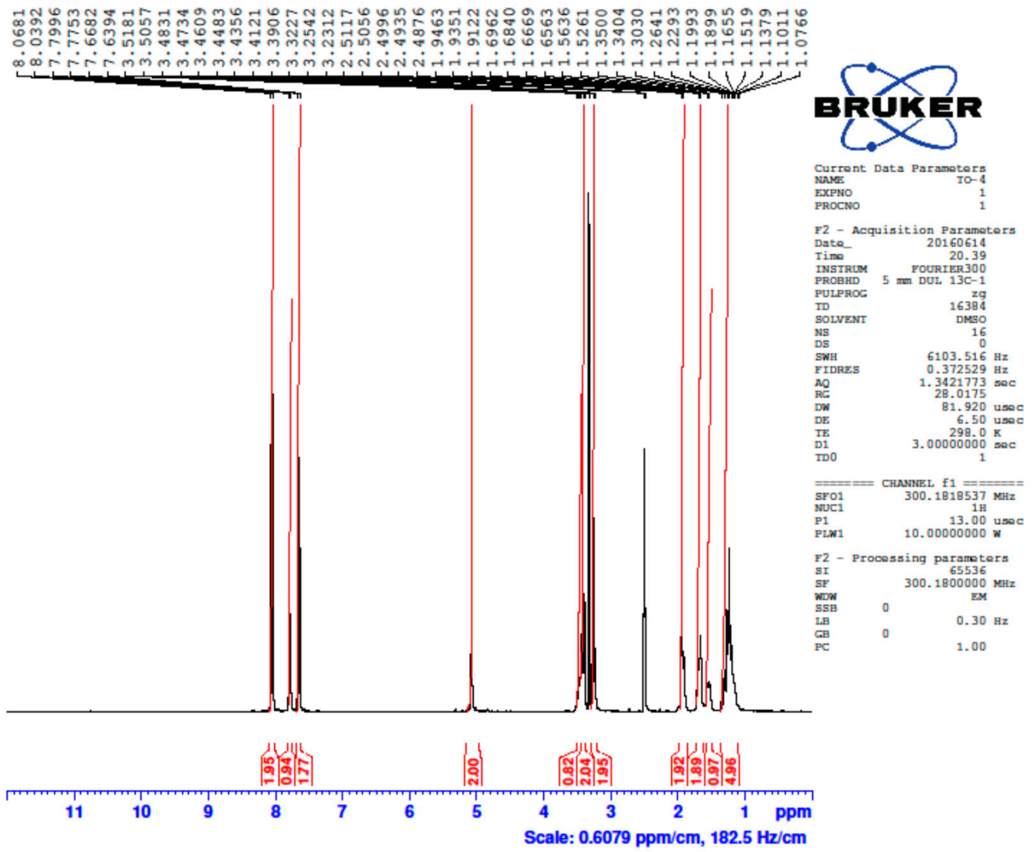

Figure 15. Compound 6d ^{1}H NMR spectrum

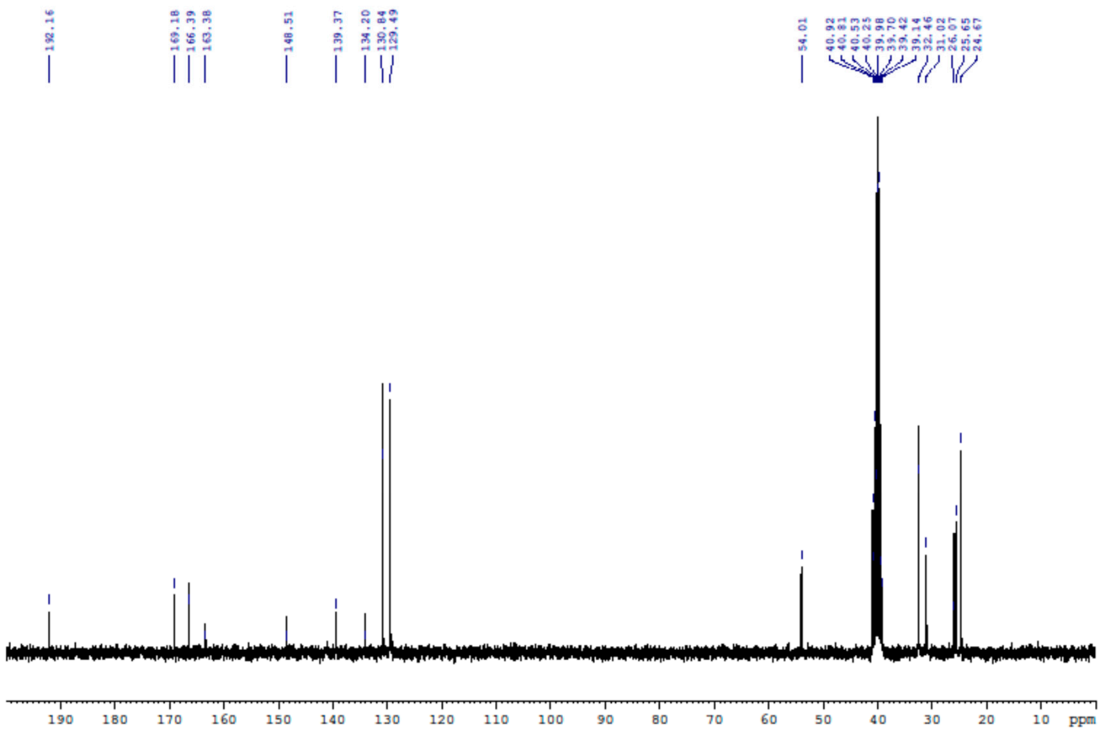

Figure 16. Compound 6d ^{13}C NMR spectrum

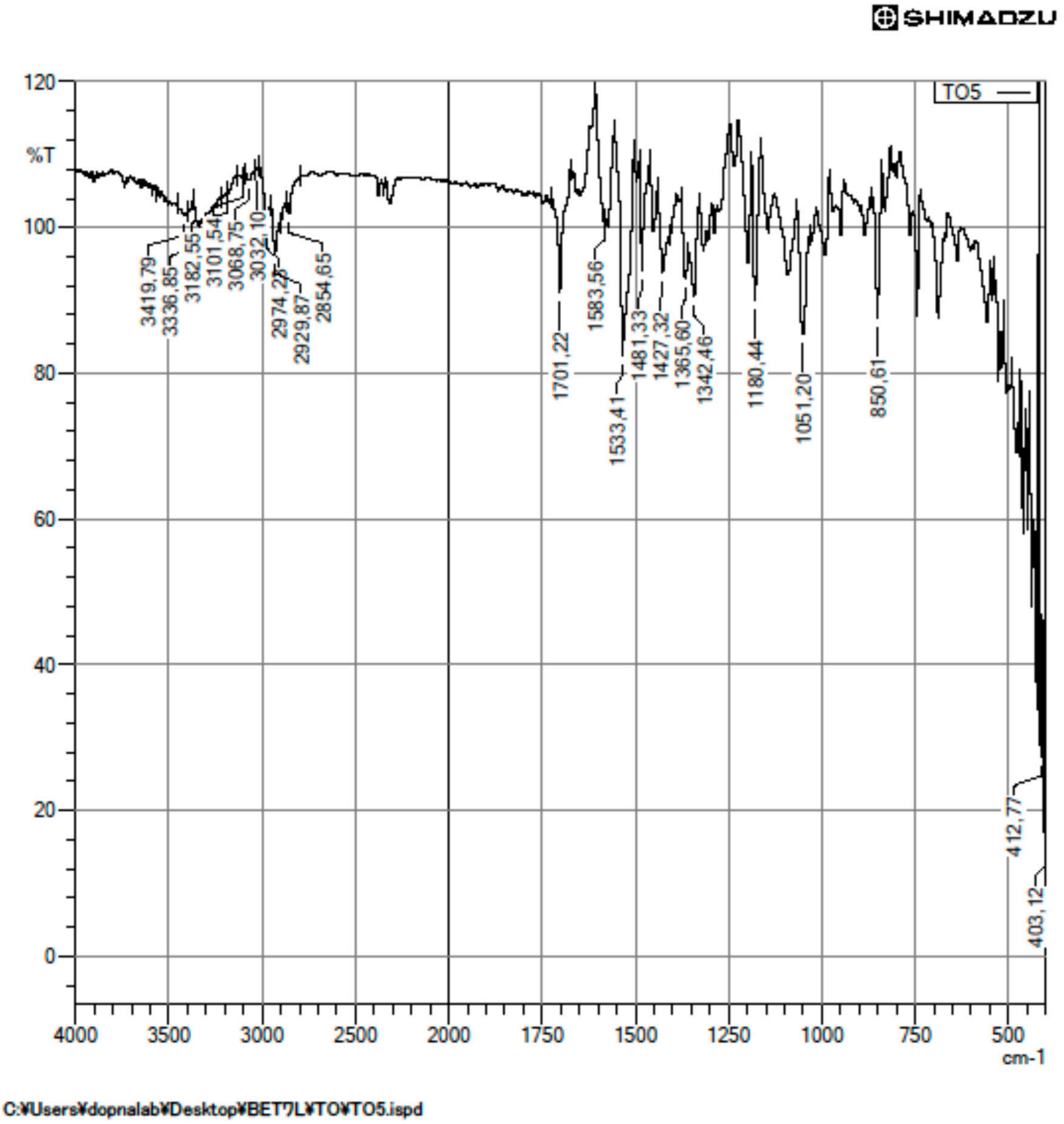

	Item	Value
2	Sample name	TO-5
3	Sample ID	
4	Option	
5	Intensity Mode	%Transmittance
6	Apodization	Happ-Genzel
9	No. of Scans	10

1

Figure 17. Compound 6e IR spectrum

Formula Predictor Report - TO5_7.lcd Page 1 of 1

Data File: C:\LabSolutions\Data\Analiz\BKaya\TO5_7.lcd

Elmt	Val.	Min	Max	Elmt	Val.	Min	Max	Elmt	Val.	Min	Max	Use Adduct
H	1	10	40	O	2	2	5	Cl	1	0	1	H
C	4	10	26	F	1	0	0	Br	1	0	0	
N	3	5	6	S	2	3	3					

Error Margin (ppm):	5	DBE Range:	0.0 - 19.0	Electron Ions:	both
HC Ratio:	unlimited	Apply N Rule:	yes	Use MSn Info:	no
Max Isotopes:	3	Isotope RI (%):	1.00	Isotope Res:	10000
MSn Iso RI (%):	10.00	MSn Logic Mode:	AND	Max Results:	500

Event#: 1 MS(E+) Ret. Time : 7.760 -> 7.827 Scan# : 1165 -> 1175

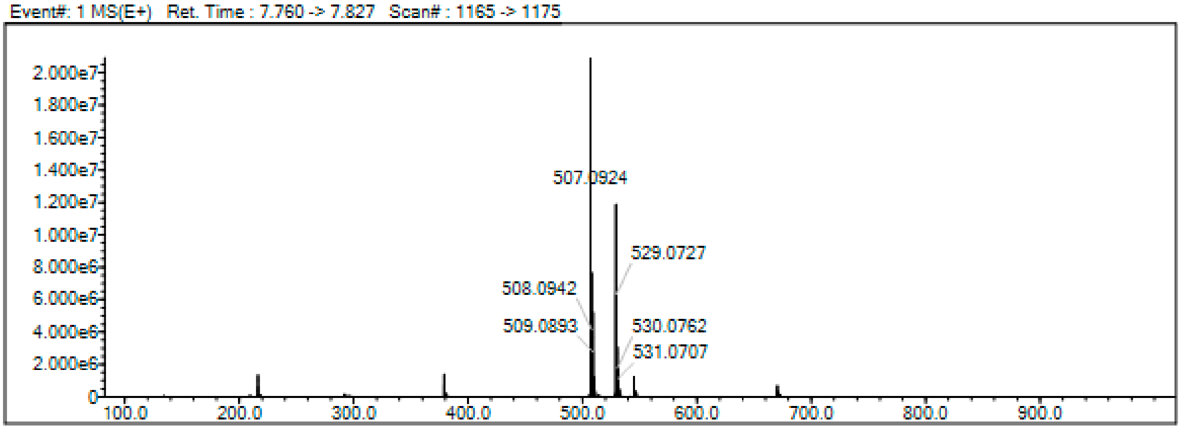

Measured region for 507.0924 m/z

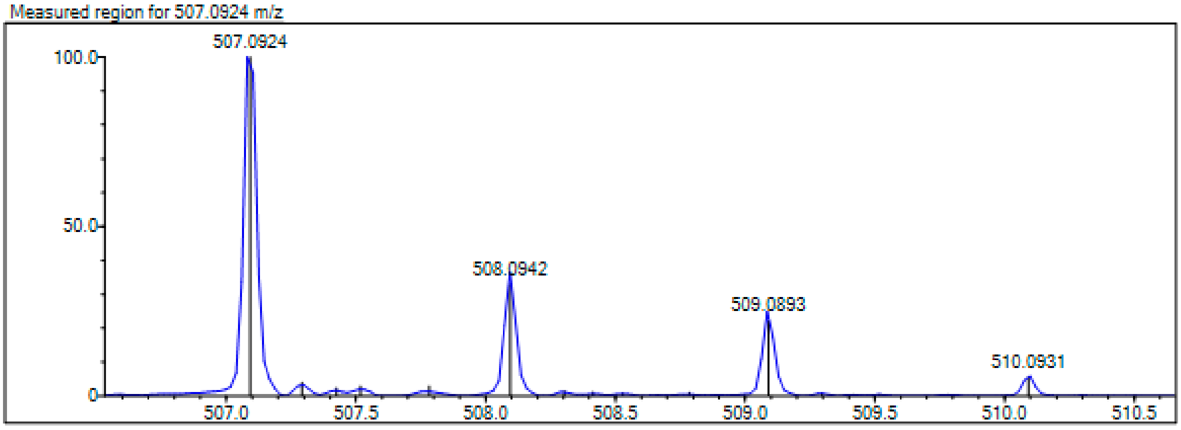

C20 H22 N6 O4 S3 [M+H]+ : Predicted region for 507.0937 m/z

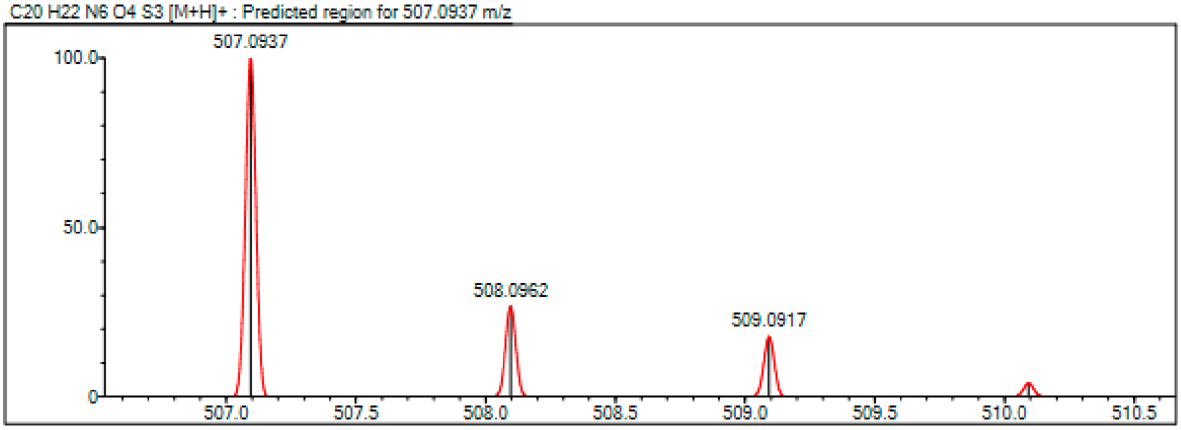

Rank	Score	Formula (M)	Ion	Meas. m/z	Pred. m/z	Df. (mDa)	Df. (ppm)	Iso	DBE
1	77.90	C20 H22 N6 O4 S3	[M+H]+	507.0924	507.0937	-1.3	-2.56	81.07	13.0

Figure 18. Compound 6e Mass spectrum

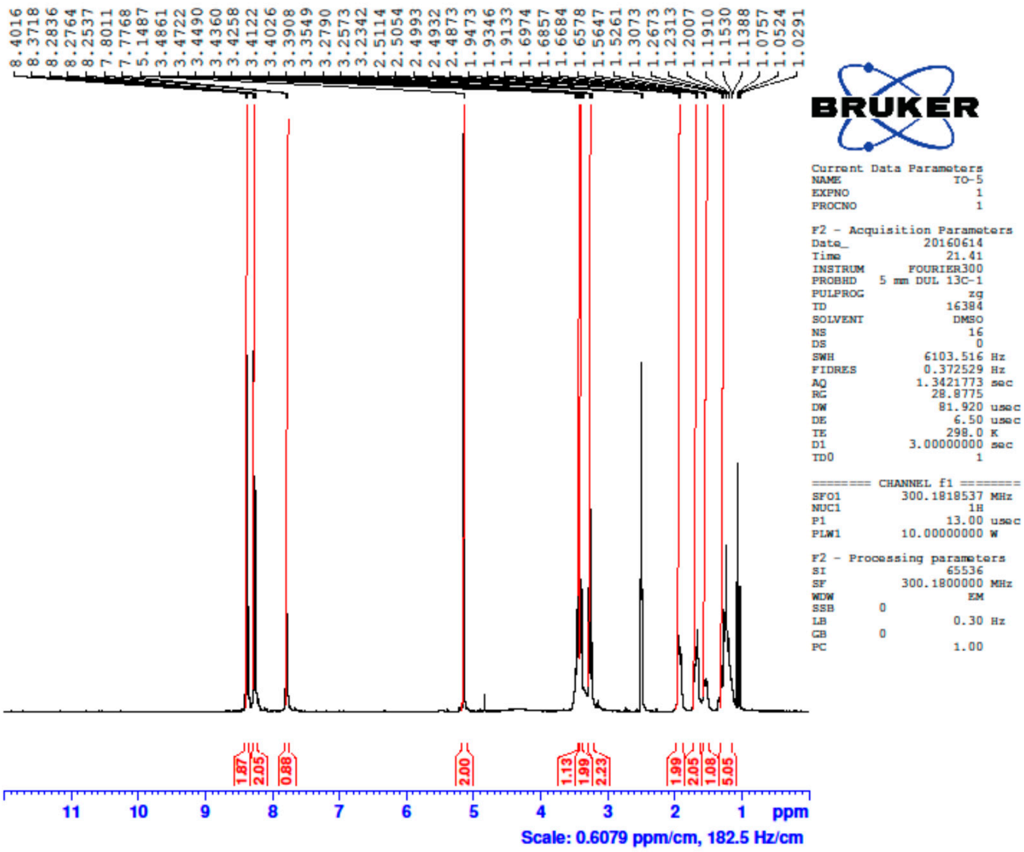

Figure 19. Compound 6e ^{1}H NMR spectrum

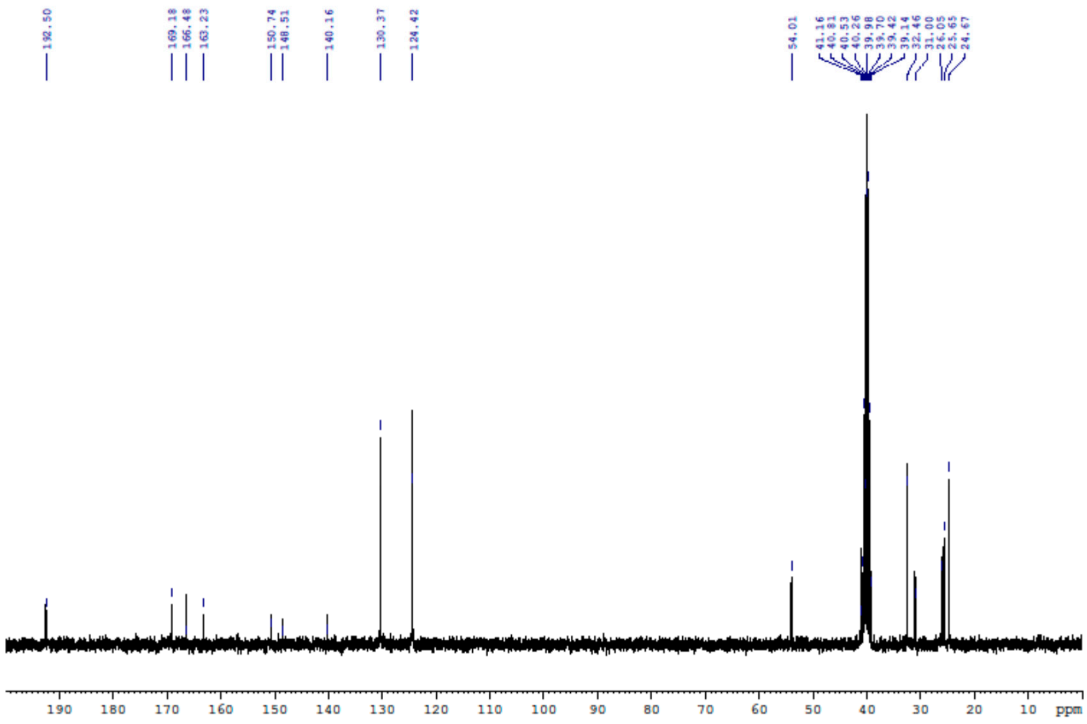

Figure 20. Compound 6e ^{13}C NMR spectrum

SHIMADZU

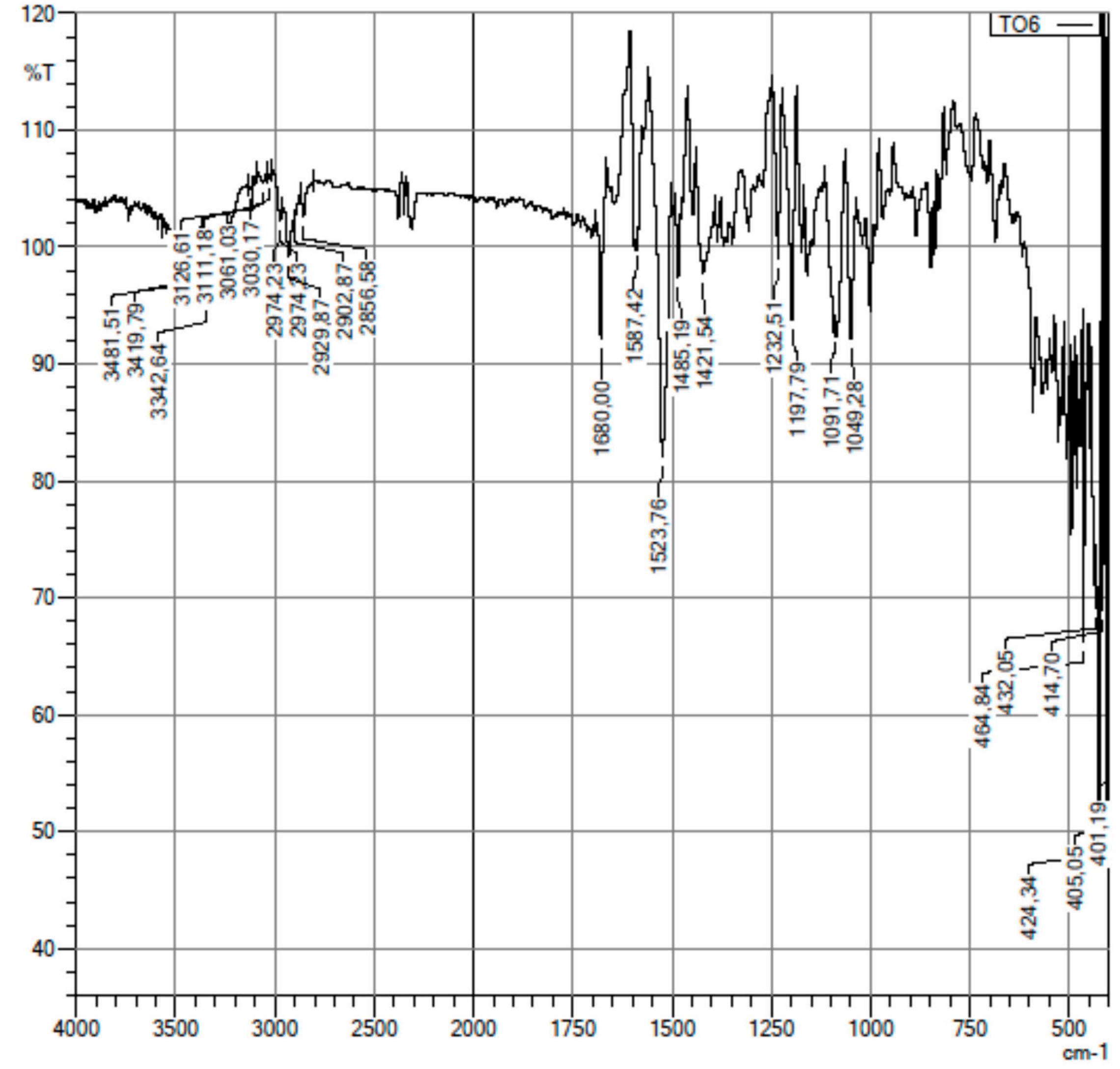

C:¥Users¥dopnalab¥Desktop¥BETワL¥TO¥TO6.ispd

	Item	Value
2	Sample name	TO-6
3	Sample ID	
4	Option	
5	Intensity Mode	%Transmittance
6	Apodization	Happ-Genzel
9	No. of Scans	10

1

Figure 21. Compound 6f IR spectrum

Formula Predictor Report - TO6_8.lcd Page 1 of 1

Data File: C:\LabSolutions\Data\Analiz\BKaya\TO6_8.lcd

Elmt	Val.	Min	Max	Elmt	Val.	Min	Max	Elmt	Val.	Min	Max	Use Adduct
H	1	10	40	O	2	2	5	Cl	1	0	1	H
C	4	10	26	F	1	1	1	Br	1	0	0	
N	3	5	6	S	2	3	3					

Error Margin (ppm):	3	DBE Range:	0.0 - 19.0	Electron Ions:	both
HC Ratio:	unlimited	Apply N Rule:	yes	Use MSn Info:	no
Max Isotopes:	3	Isotope RI (%):	1.00	Isotope Res:	10000
MSn Iso RI (%):	10.00	MSn Logic Mode:	AND	Max Results:	500

Event#: 1 MS(E+) Ret. Time : 7.827 -> 7.867 Scan# : 1175 -> 1181

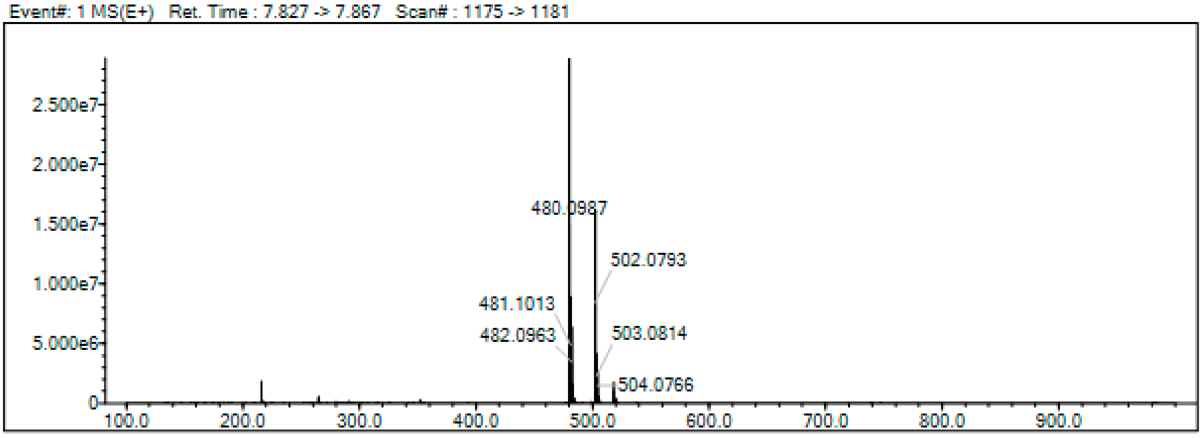

Measured region for 480.0987 m/z

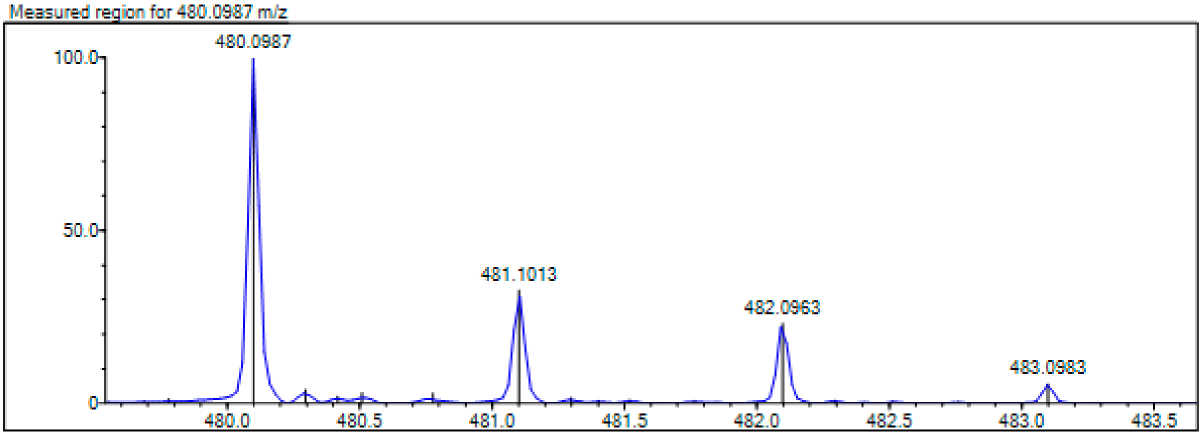

C20 H22 N5 O2 F S3 [M+H]+ : Predicted region for 480.0992 m/z

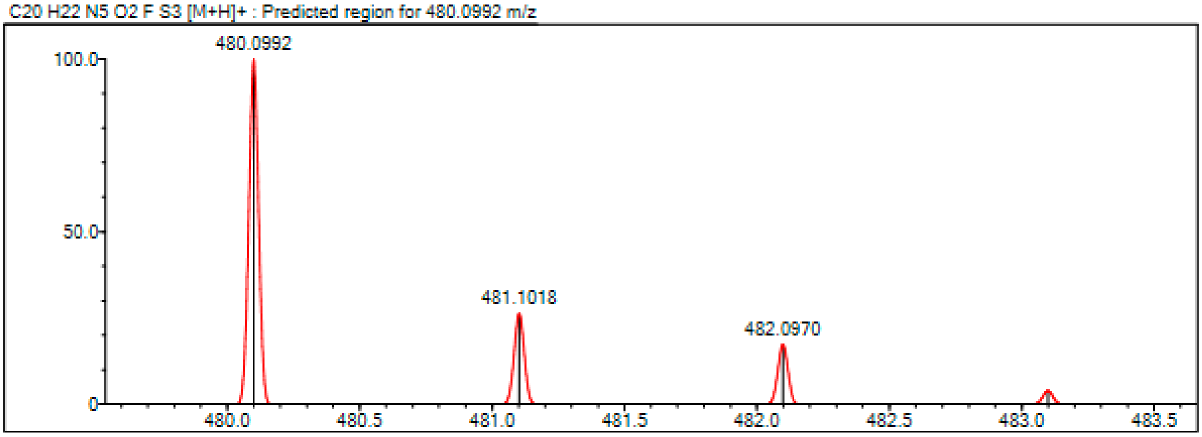

Rank	Score	Formula (M)	Ion	Meas. m/z	Pred. m/z	Df. (mDa)	Df. (ppm)	Iso	DBE
1	91.96	C20 H22 N5 O2 F S3	[M+H]+	480.0987	480.0992	-0.5	-1.04	92.05	12.0

Figure 22. Compound 6f Mass spectrum

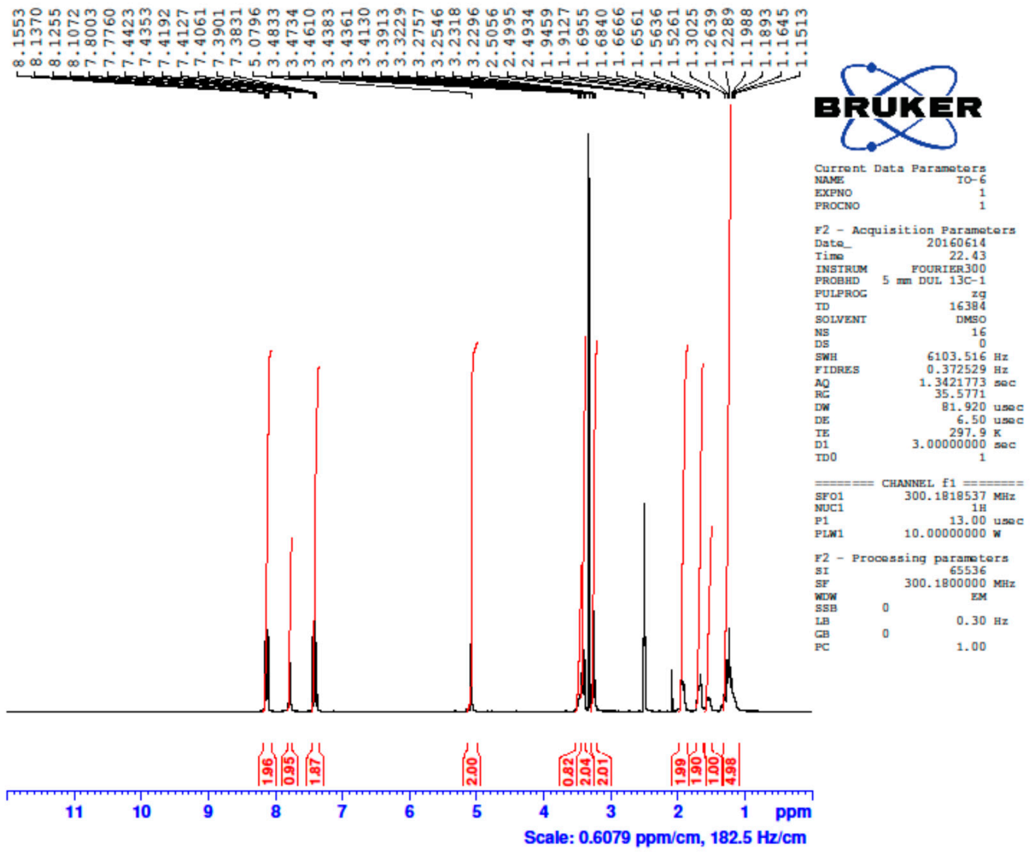

Figure 23. Compound 6f ^{1}H NMR spectrum

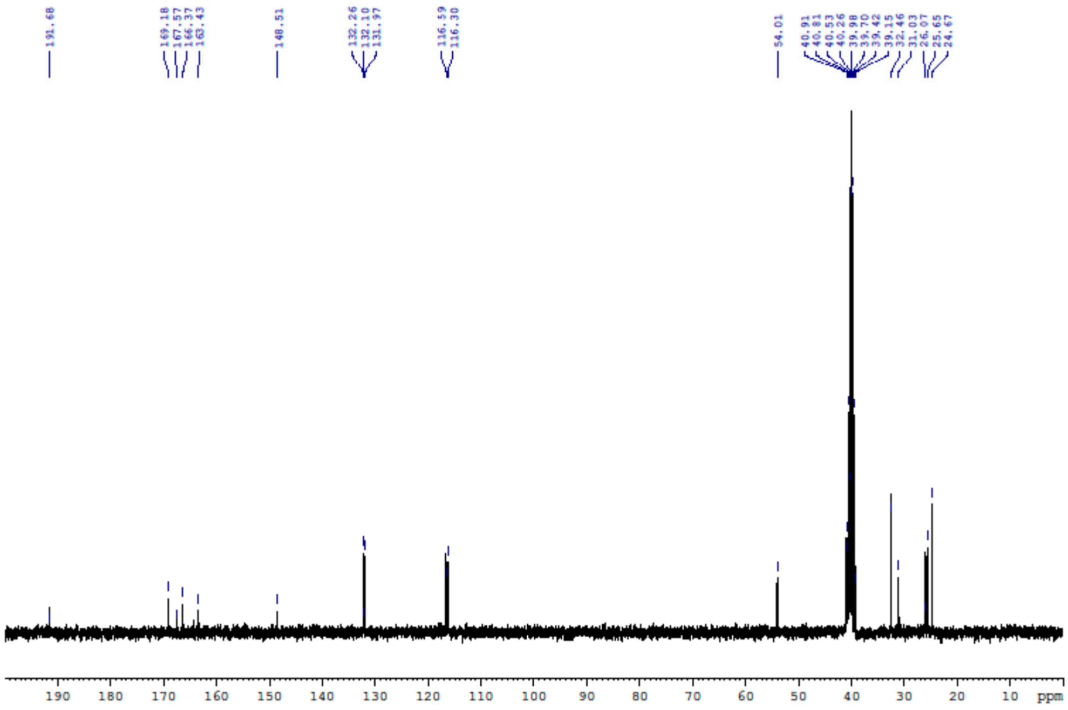

Figure 24. Compound 6f ^{13}C NMR spectrum

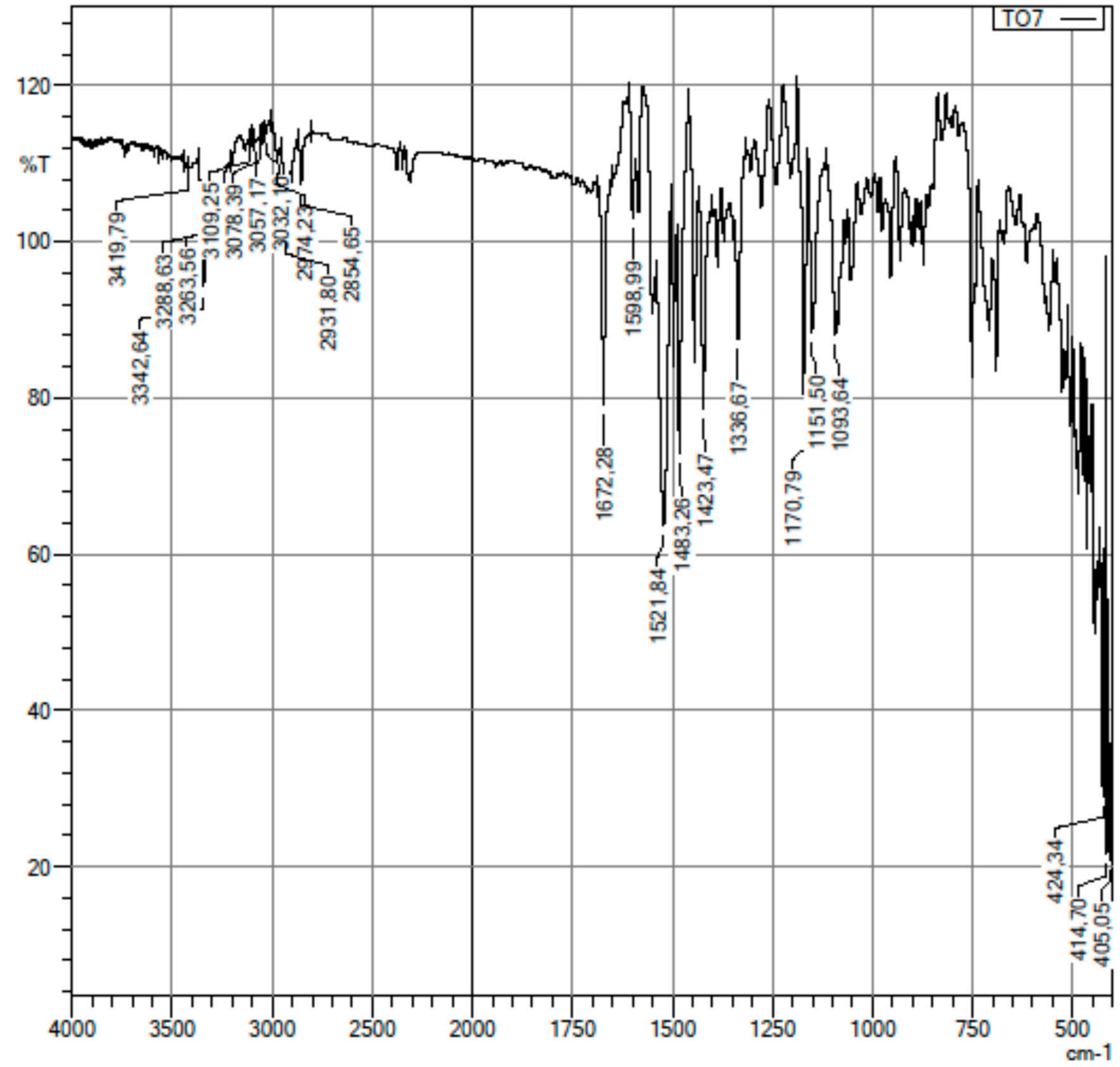

C:¥Users¥dopnalab¥Desktop¥BETワL¥TO¥TO7.ispd

	Item	Value
2	Sample name	TO-7
3	Sample ID	
4	Option	
5	Intensity Mode	%Transmittance
6	Apodization	Happ-Genzel
9	No. of Scans	10

1

Figure 25. Compound 6g IR spectrum

Data File: C:\LabSolutions\Data\Analiz\BKaya\TO7_9.lcd

Elmt	Val.	Min	Max	Elmt	Val.	Min	Max	Elmt	Val.	Min	Max	Use Adduct
H	1	10	40	O	2	2	5	Cl	1	0	1	H
C	4	10	26	F	1	0	1	Br	1	0	0	
N	3	5	6	S	2	3	3					

Error Margin (ppm): 3
HC Ratio: unlimited
Max Isotopes: 3
MSn Iso RI (%): 10.00

DBE Range: 0.0 - 19.0
Apply N Rule: yes
Isotope RI (%): 1.00
MSn Logic Mode: AND

Electron Ions: both
Use MSn Info: no
Isotope Res: 10000
Max Results: 500

Event#: 1 MS(E+) Ret. Time : 7.440 -> 7.480 Scan# : 1117 -> 1123

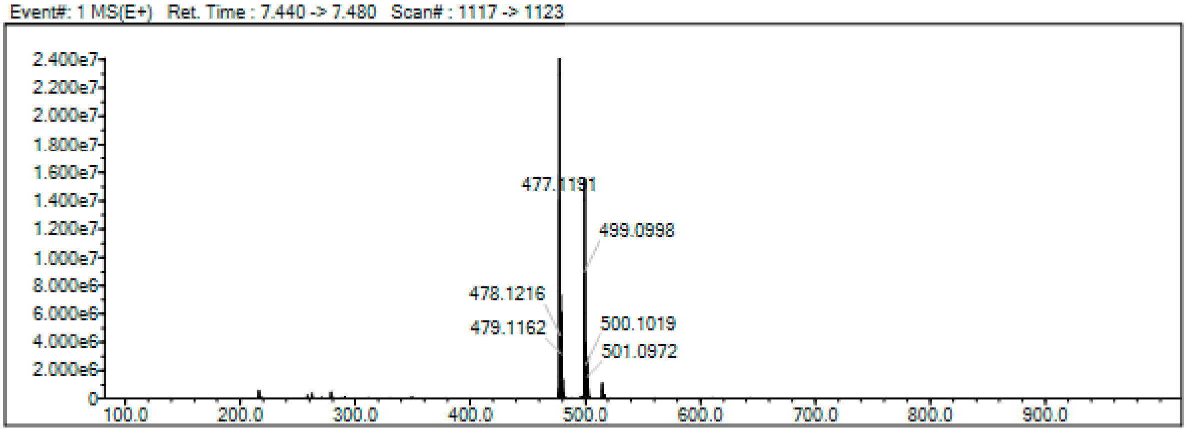

Measured region for 477.1191 m/z

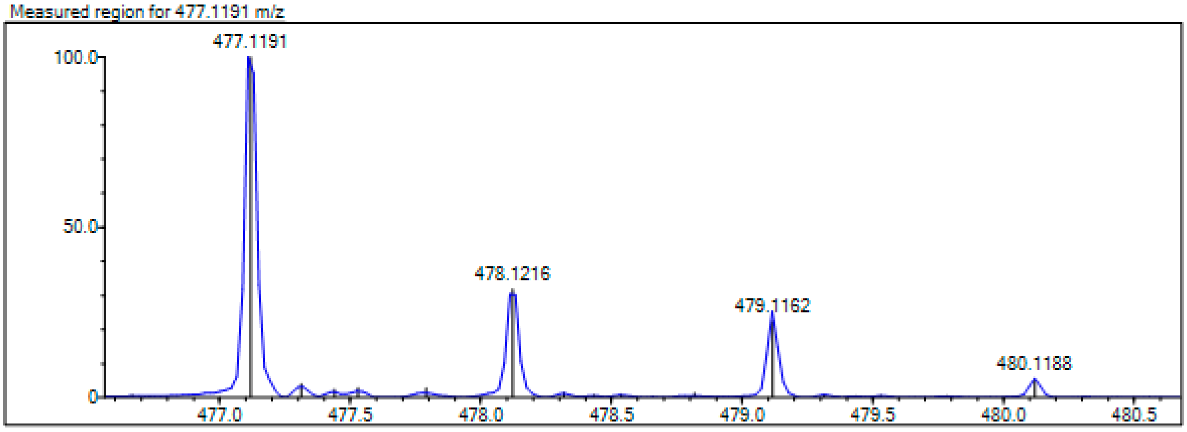

C20 H24 N6 O2 S3 [M+H]+ : Predicted region for 477.1196 m/z

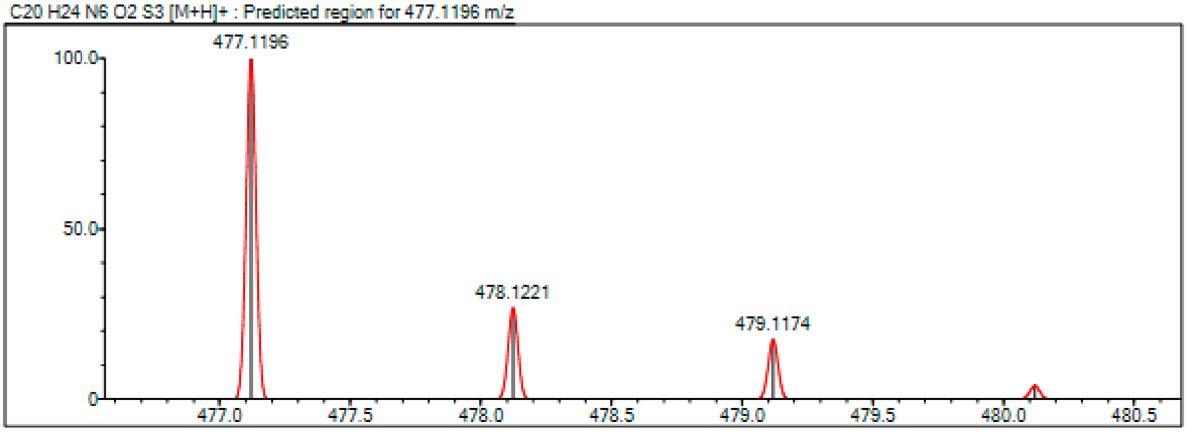

Rank	Score	Formula (M)	Ion	Meas. m/z	Pred. m/z	Df. (mDa)	Df. (ppm)	Iso	DBE
1	84.11	C20 H24 N6 O2 S3	[M+H]+	477.1191	477.1196	-0.5	-1.05	84.22	12.0

Figure 26. Compound 6g Mass spectrum

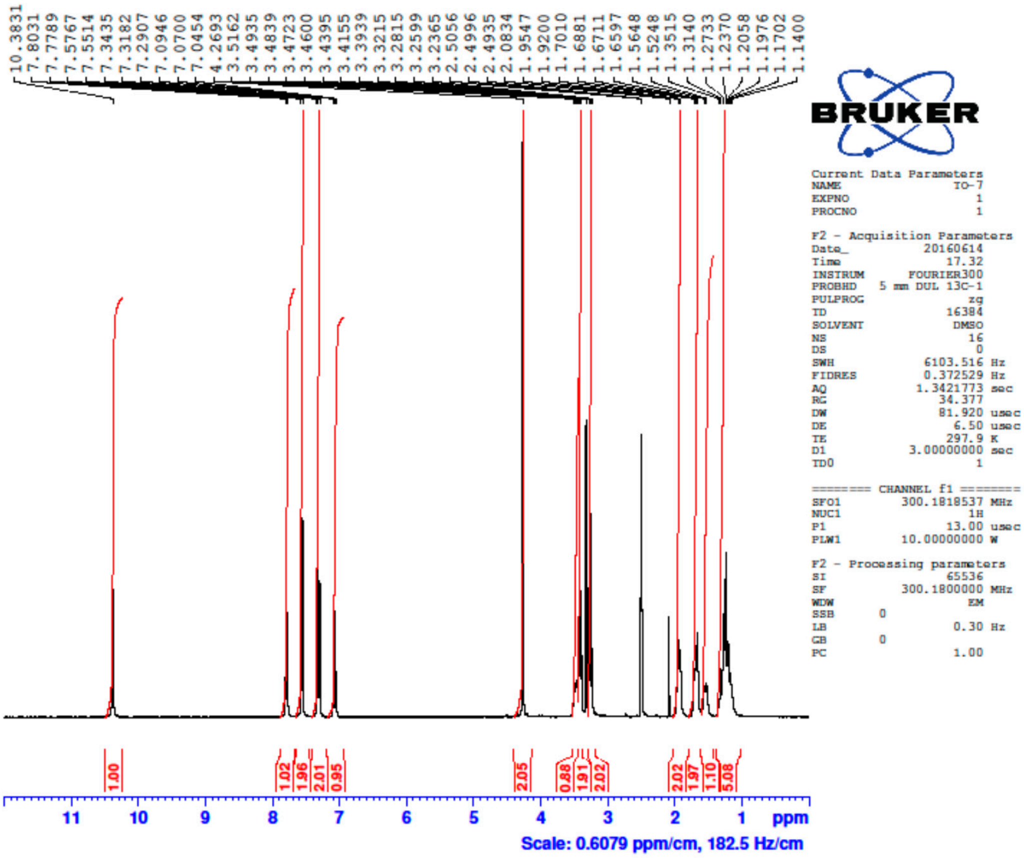

Figure 27. Compound 6g ^{1}H NMR spectrum

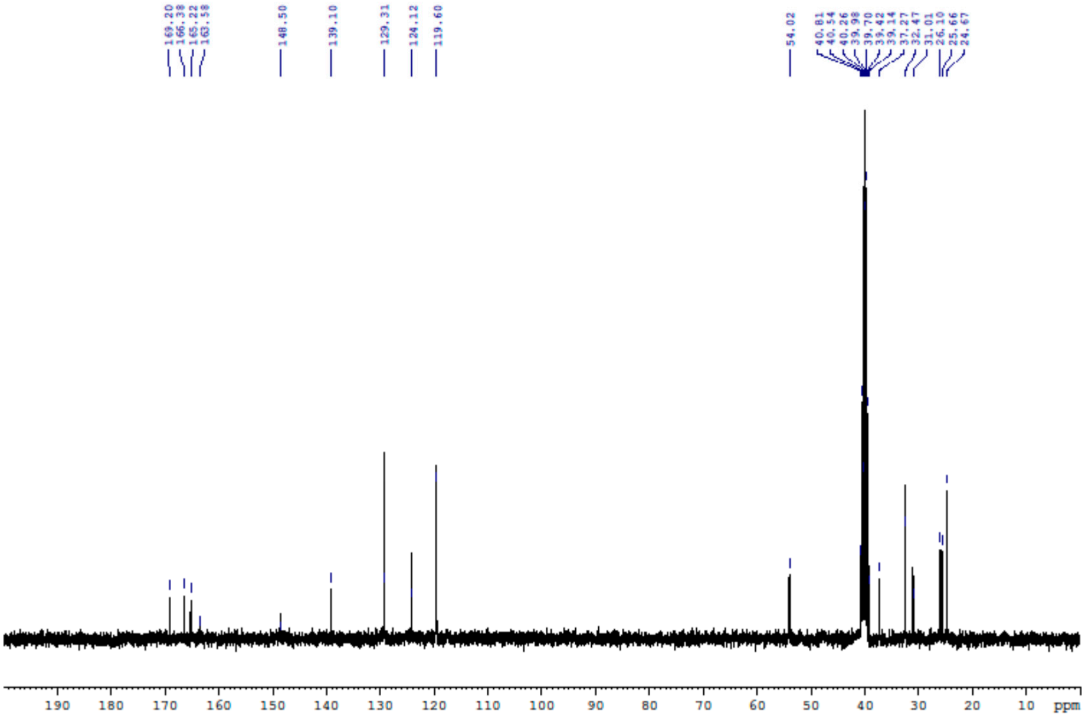

Figure 28. Compound 6g ^{13}C NMR spectrum

SHIMADZU

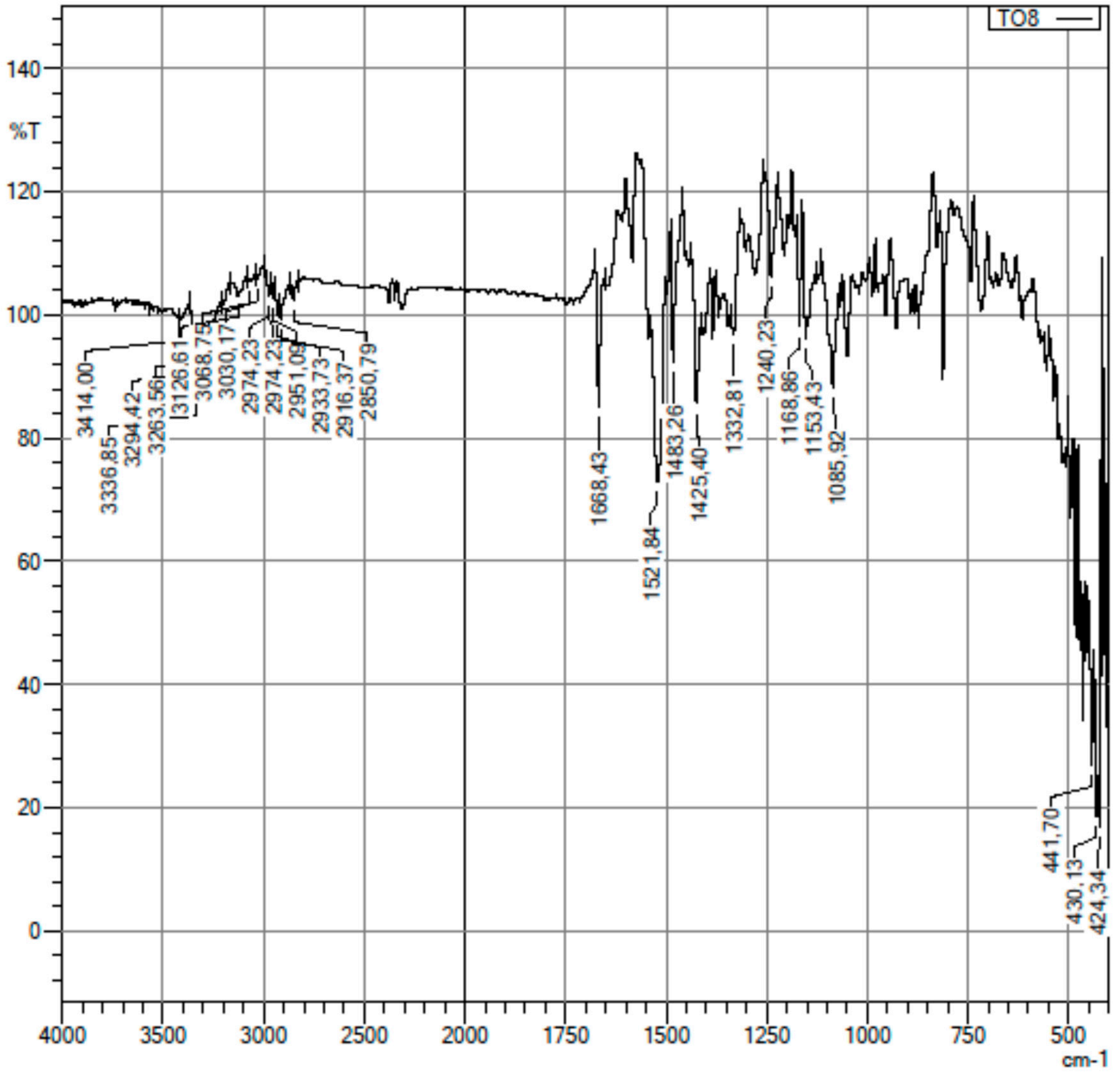

C:¥Users¥dopnalab¥Desktop¥BETワL¥TO¥TO8.ispd

	Item	Value
2	Sample name	TO-8
3	Sample ID	
4	Option	
5	Intensity Mode	%Transmittance
6	Apodization	Happ-Genzel
9	No. of Scans	10

1

Figure 29. Compound 6h IR spectrum

Formula Predictor Report - TO8_10.lcd — Page 1 of 1

Data File: C:\LabSolutions\Data\Analiz\BKaya\TO8_10.lcd

Elmt	Val.	Min	Max	Elmt	Val.	Min	Max	Elmt	Val.	Min	Max	Use Adduct
H	1	10	40	O	2	2	5	Cl	1	0	0	H
C	4	10	26	F	1	0	1	Br	1	0	0	
N	3	5	6	S	2	3	3					

Error Margin (ppm):	5	DBE Range:	9.0 - 19.0	Electron Ions:	both
HC Ratio:	unlimited	Apply N Rule:	yes	Use MSn Info:	no
Max Isotopes:	3	Isotope RI (%):	1.00	Isotope Res:	10000
MSn Iso RI (%):	10.00	MSn Logic Mode:	AND	Max Results:	500

Event#: 1 MS(E+) Ret. Time : 7.720 -> 7.787 Scan# : 1159 -> 1169

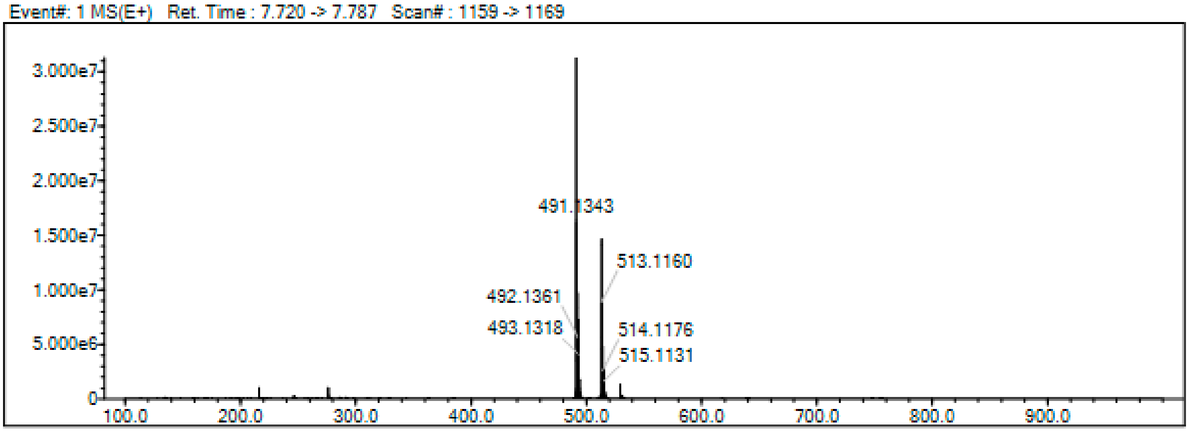

Measured region for 491.1343 m/z

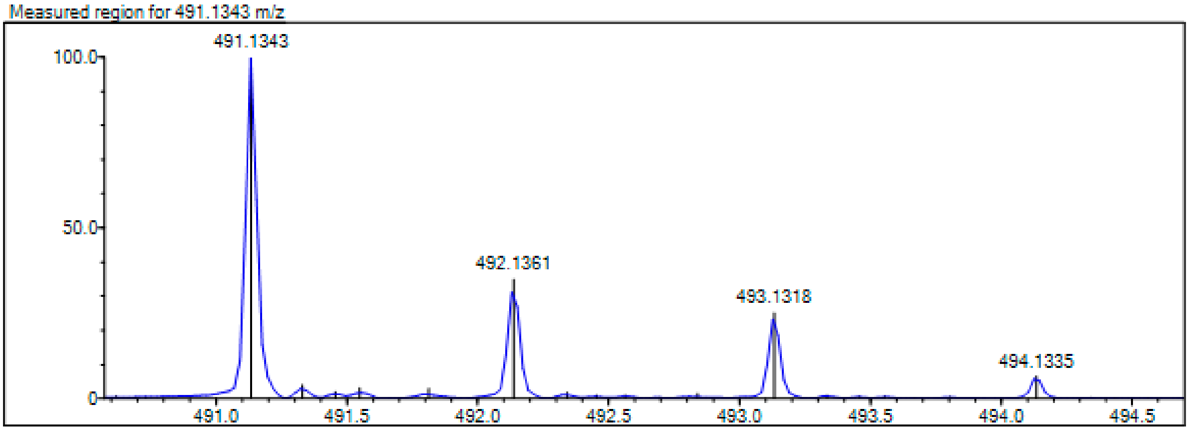

C21 H26 N6 O2 S3 [M+H]+ : Predicted region for 491.1352 m/z

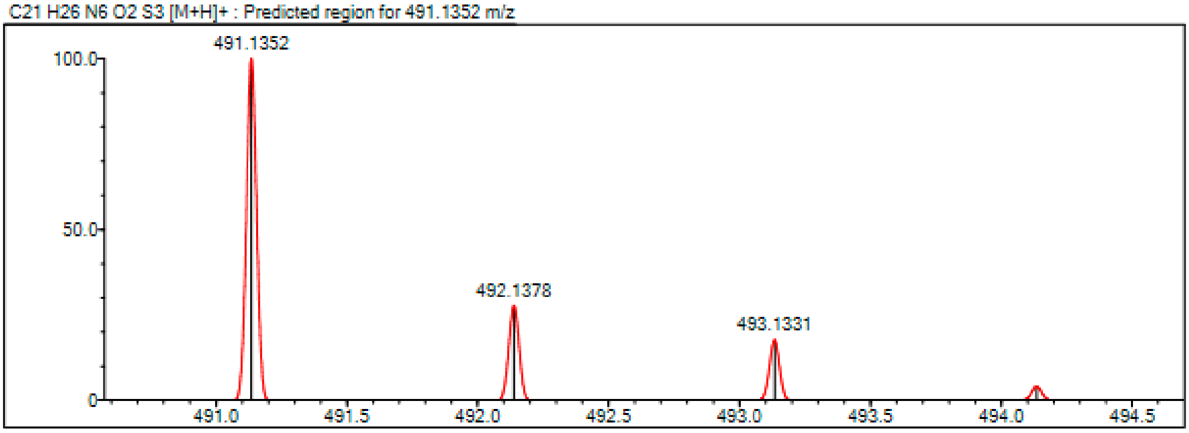

Rank	Score	Formula (M)	Ion	Meas. m/z	Pred. m/z	Df. (mDa)	Df. (ppm)	Iso	DBE
1	88.03	C21 H26 N6 O2 S3	[M+H]+	491.1343	491.1352	-0.9	-1.83	89.90	12.0

Figure 30. Compound 6h Mass spectrum

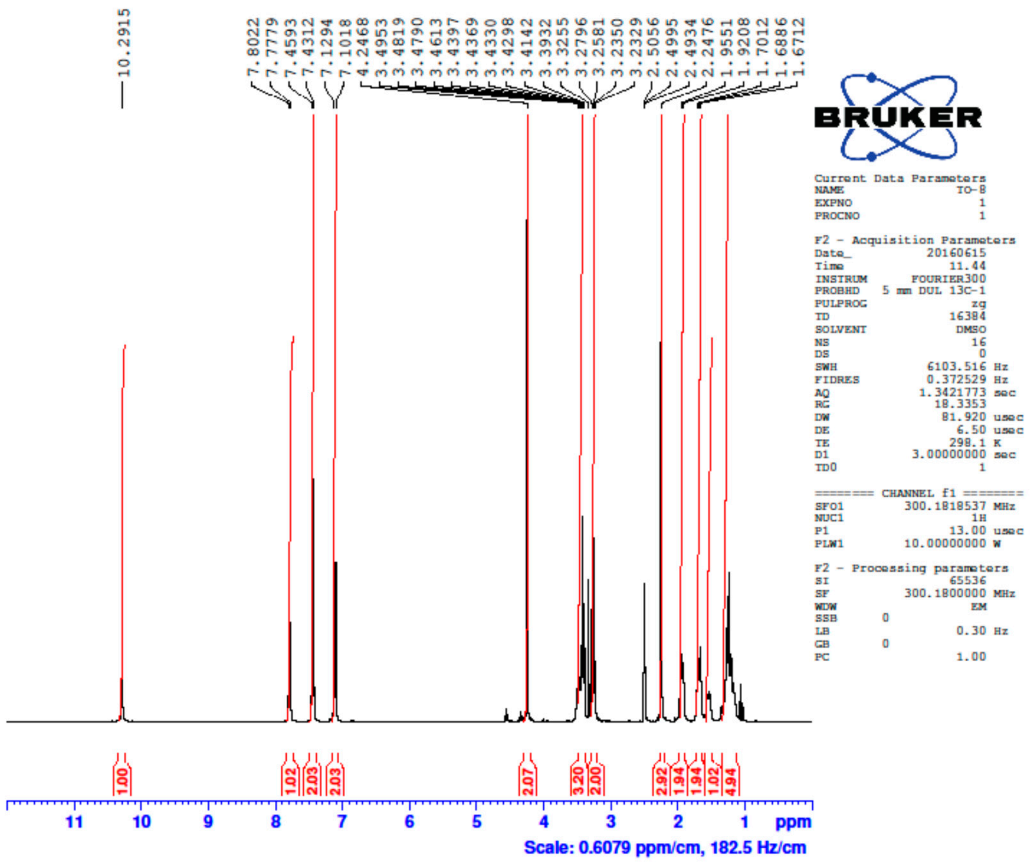

Figure 31. Compound 6h ^{1}H NMR spectrum

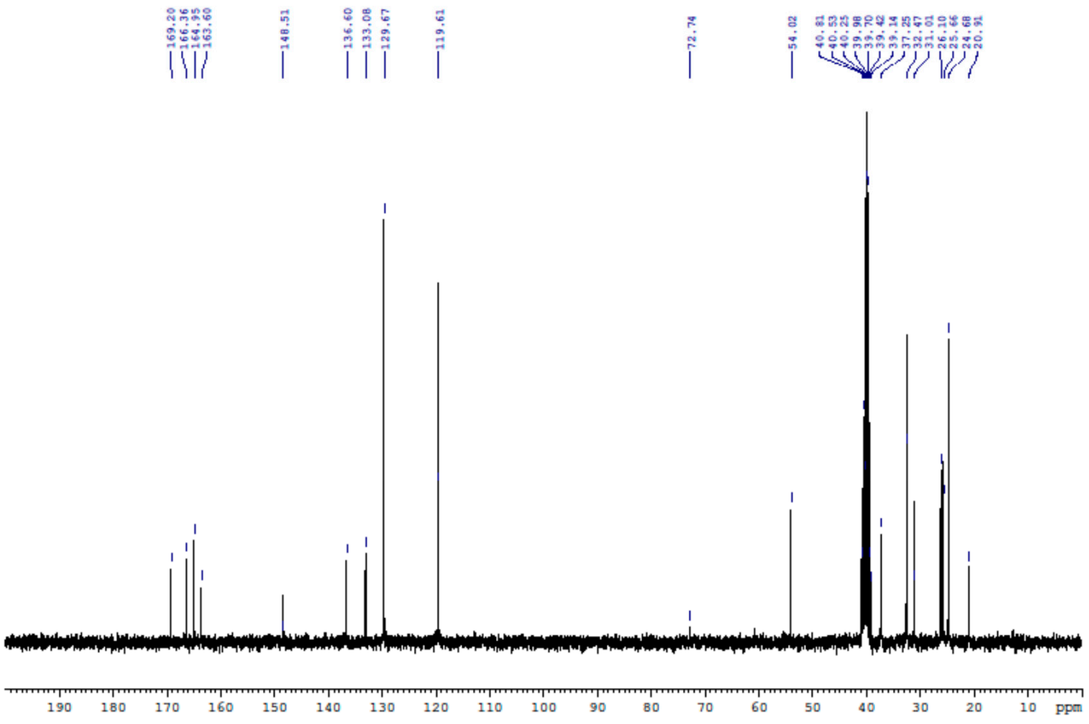

Figure 32. Compound 6h ^{13}C NMR spectrum

SHIMADZU

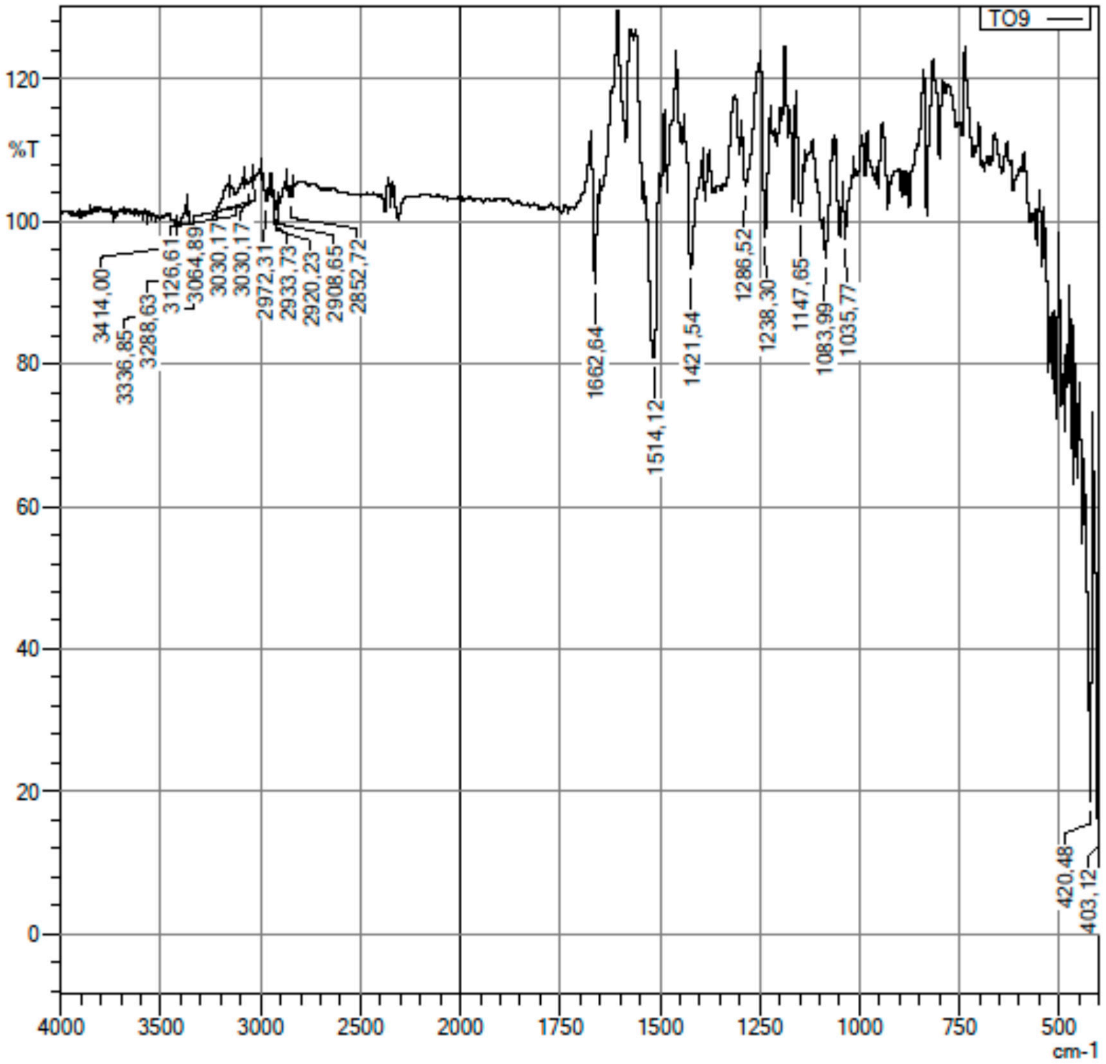

C:¥Users¥dopnalab¥Desktop¥BET7L¥TO¥TO9.ispd

	Item	Value
2	Sample name	TO-9
3	Sample ID	
4	Option	
5	Intensity Mode	%Transmittance
6	Apodization	Happ-Genzel
9	No. of Scans	10

Figure 33. Compound 6i IR spectrum

Formula Predictor Report - TO9_11.lcd

Page 1 of 1

Data File: C:\LabSolutions\Data\Analiz\BKaya\TO9_11.lcd

Elmt	Val.	Min	Max	Elmt	Val.	Min	Max	Elmt	Val.	Min	Max	Use Adduct
H	1	10	40	O	2	2	5	Cl	1	0	0	H
C	4	10	26	F	1	0	1	Br	1	0	0	
N	3	5	6	S	2	3	3					

Error Margin (ppm):	5	DBE Range:	9.0 - 19.0	Electron Ions:	both
HC Ratio:	unlimited	Apply N Rule:	yes	Use MSn Info:	no
Max Isotopes:	3	Isotope RI (%):	1.00	Isotope Res:	10000
MSn Iso RI (%):	10.00	MSn Logic Mode:	AND	Max Results:	500

Event#: 1 MS(E+) Ret. Time : 7.307 -> 7.360 Scan# : 1097 -> 1105

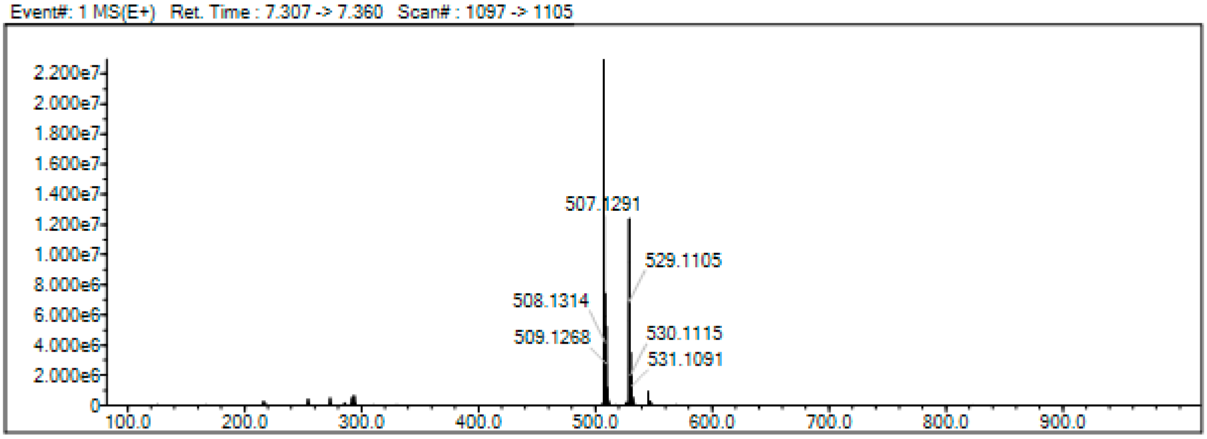

Measured region for 507.1291 m/z

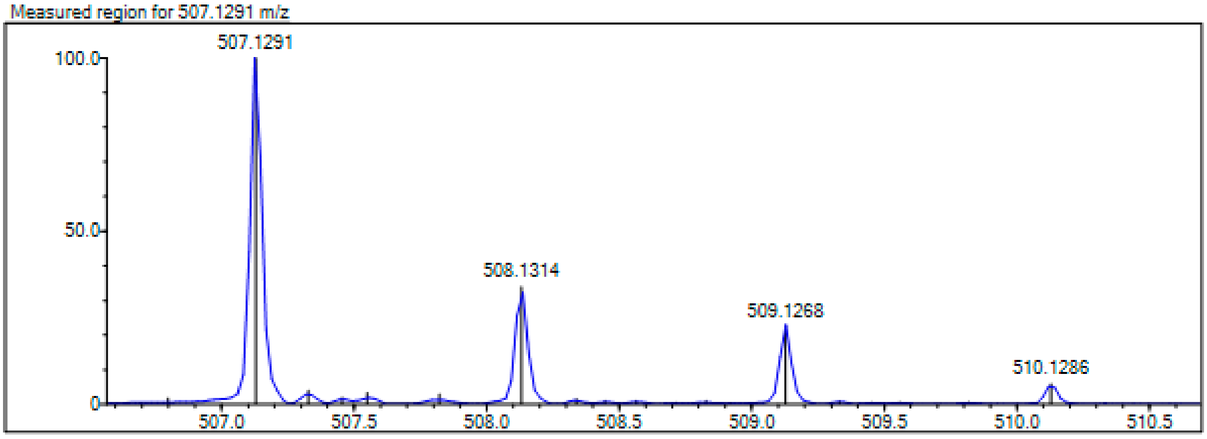

C21 H26 N6 O3 S3 [M+H]+ : Predicted region for 507.1301 m/z

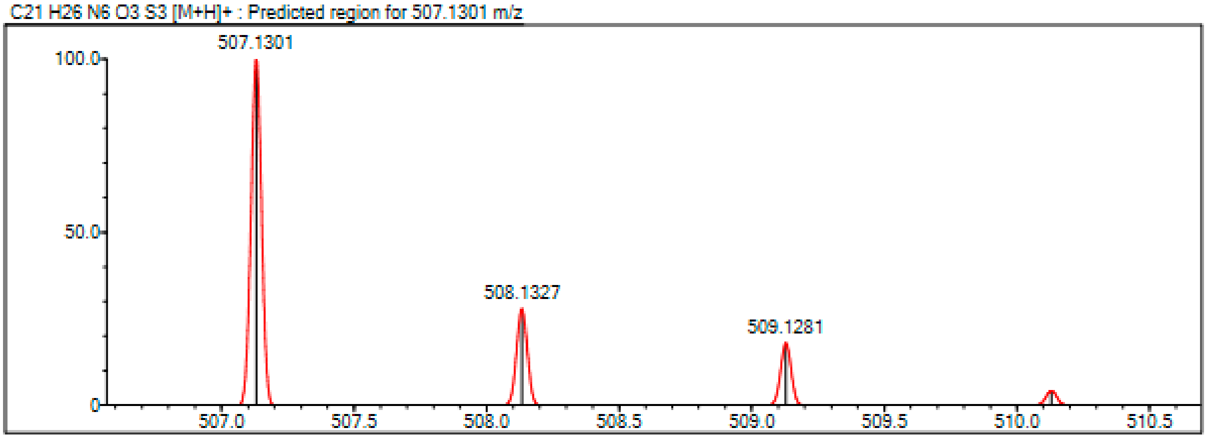

Rank	Score	Formula (M)	Ion	Meas. m/z	Pred. m/z	Df. (mDa)	Df. (ppm)	Iso	DBE
1	79.14	C21 H26 N6 O3 S3	[M+H]+	507.1291	507.1301	-1.0	-1.97	81.10	12.0

Figure 34. Compound 6i Mass spectrum

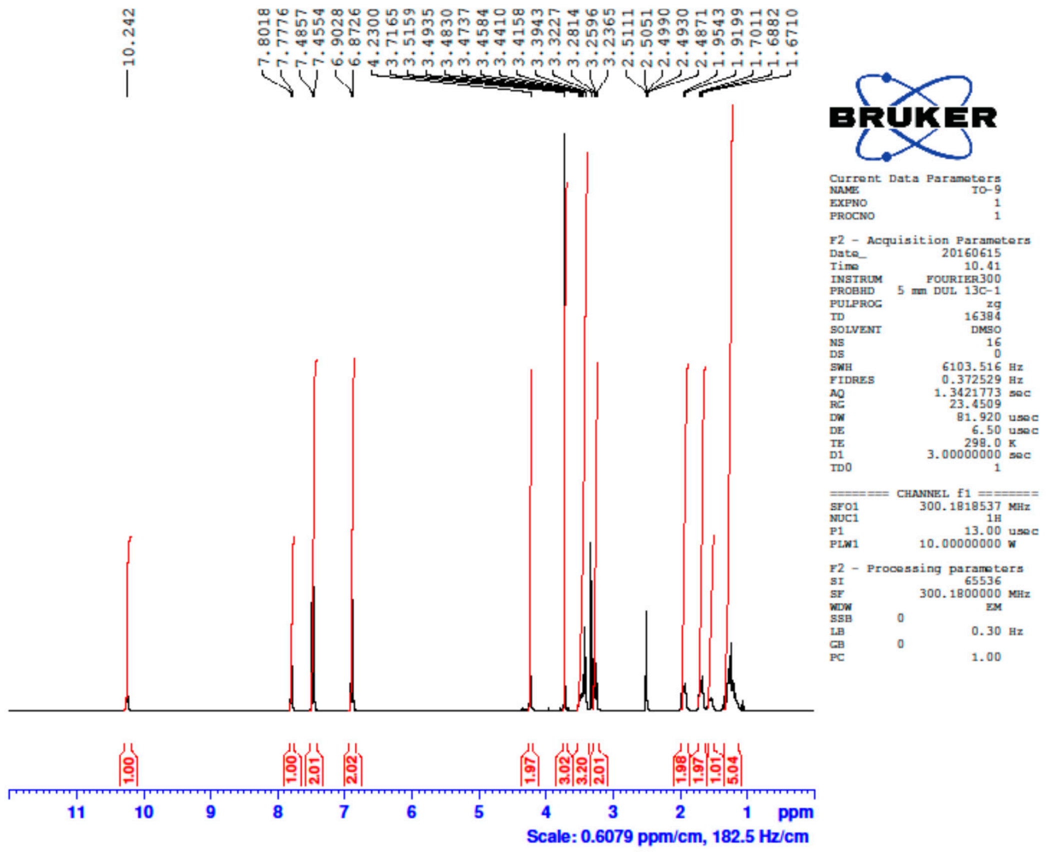

Figure 35. Compound 6i ^{1}H NMR spectrum

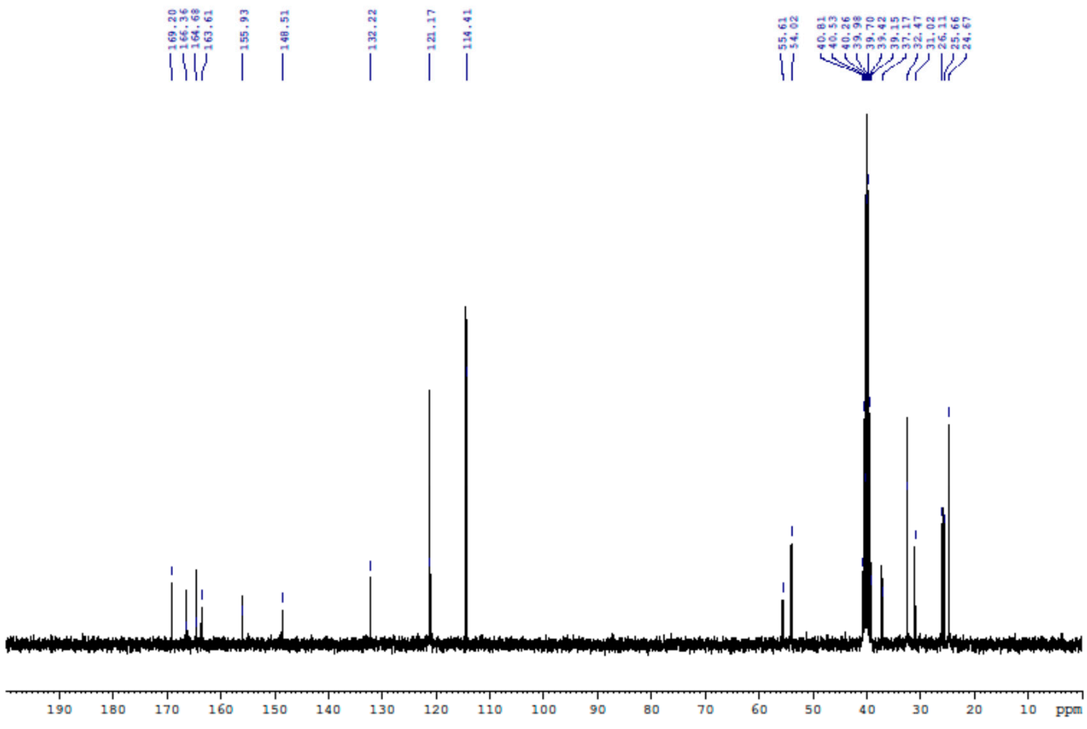

Figure 36. Compound 6i ^{13}C NMR spectrum

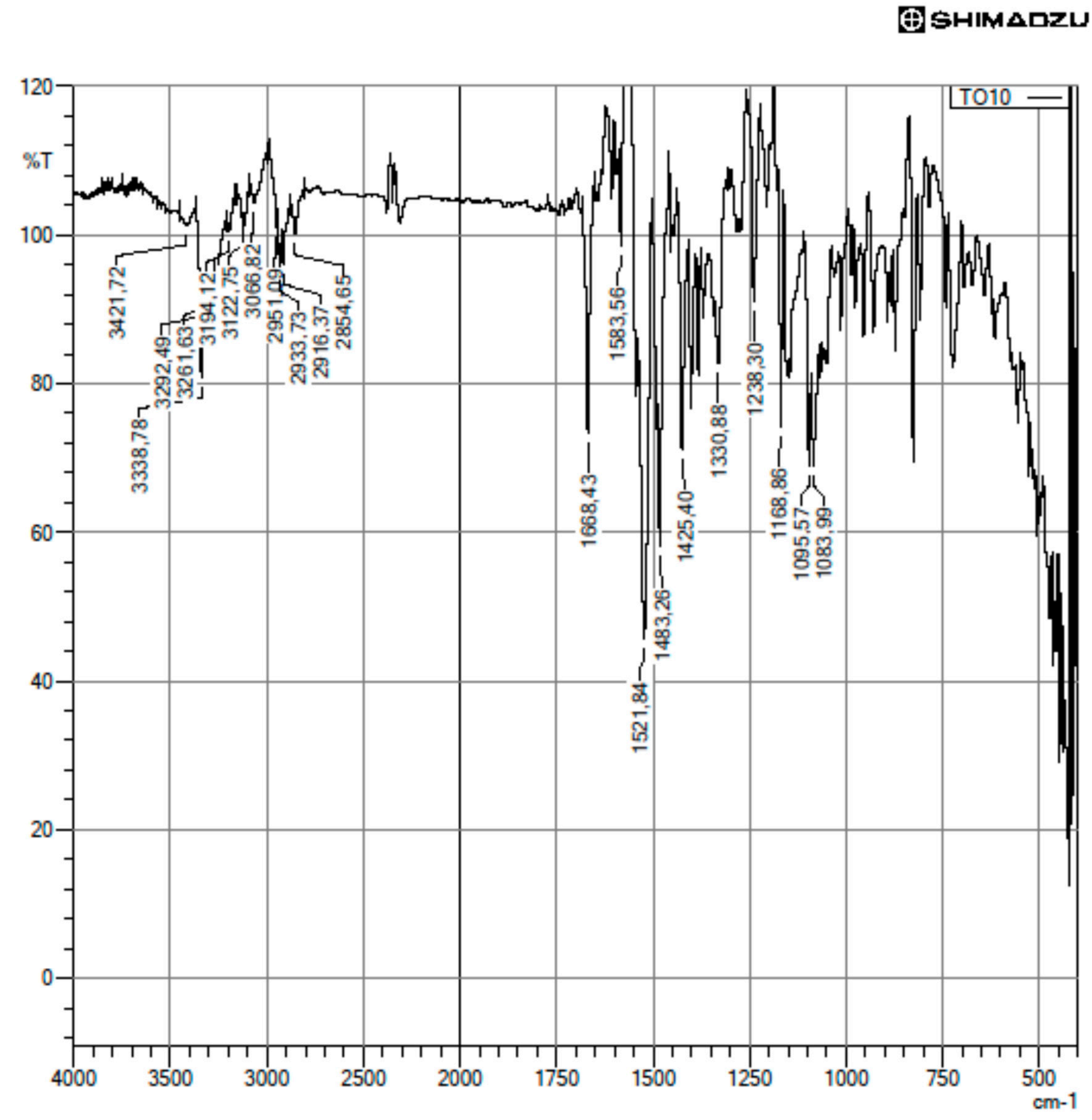

C:¥Users¥dopnalab¥Desktop¥BET7L¥TO¥TO10.ispd

	Item	Value
2	Sample name	TO-10
3	Sample ID	
4	Option	
5	Intensity Mode	%Transmittance
6	Apodization	Happ-Genzel
9	No. of Scans	10

Figure 37. Compound 6j IR spectrum

Data File: C:\LabSolutions\Data\Analiz\BKaya\TO10_12.lcd

Elmt	Val.	Min	Max	Elmt	Val.	Min	Max	Elmt	Val.	Min	Max	Use Adduct
H	1	10	40	O	2	2	5	Cl	1	1	1	H
C	4	10	26	F	1	0	1	Br	1	0	0	
N	3	5	6	S	2	3	3					

Error Margin (ppm):	5	DBE Range:	9.0 - 19.0	Electron Ions:	both
HC Ratio:	unlimited	Apply N Rule:	yes	Use MSn Info:	no
Max Isotopes:	3	Isotope RI (%):	1.00	Isotope Res:	10000
MSn Iso RI (%):	10.00	MSn Logic Mode:	AND	Max Results:	500

Event#: 1 MS(E+) Ret. Time : 7.787 -> 7.853 Scan# : 1169 -> 1179

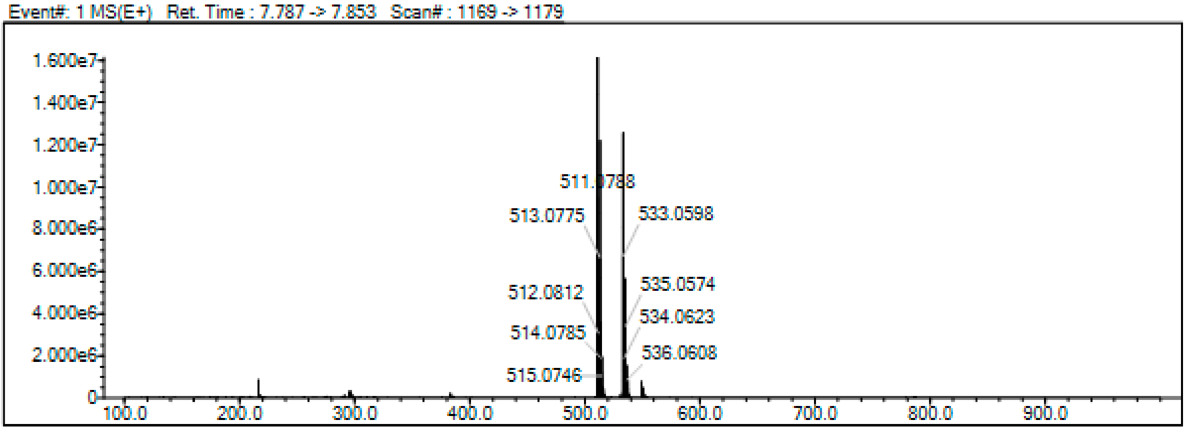

Measured region for 511.0788 m/z

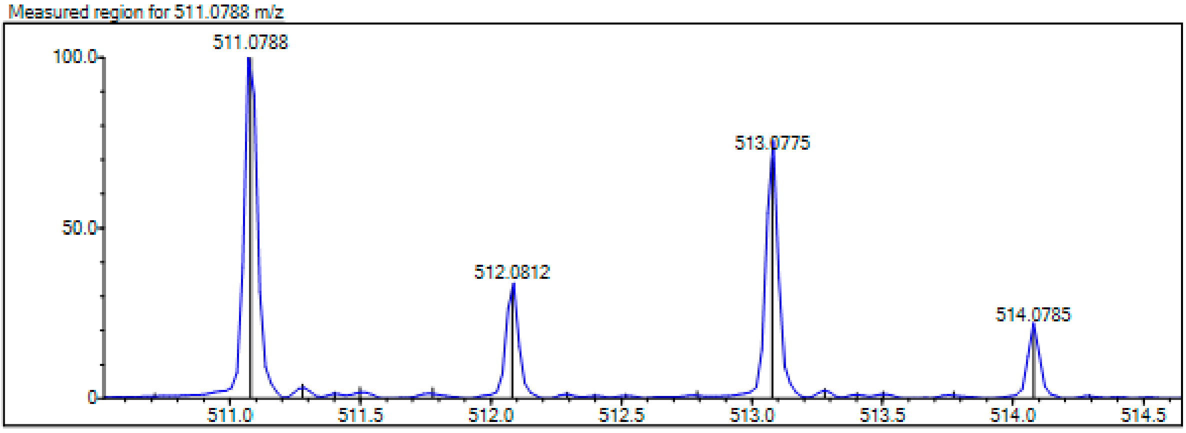

C20 H23 N6 O2 S3 Cl [M+H]+ : Predicted region for 511.0806 m/z

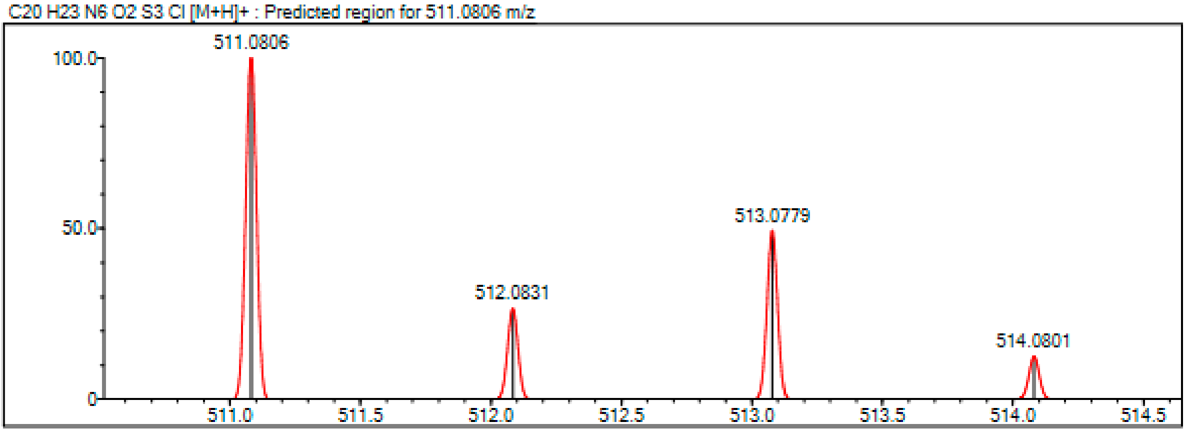

Rank	Score	Formula (M)	Ion	Meas. m/z	Pred. m/z	Df. (mDa)	Df. (ppm)	Iso	DBE
1	53.62	C20 H23 N6 O2 S3 Cl	[M+H]+	511.0788	511.0806	-1.8	-3.52	57.23	12.0

Figure 38. Compound 6j Mass spectrum

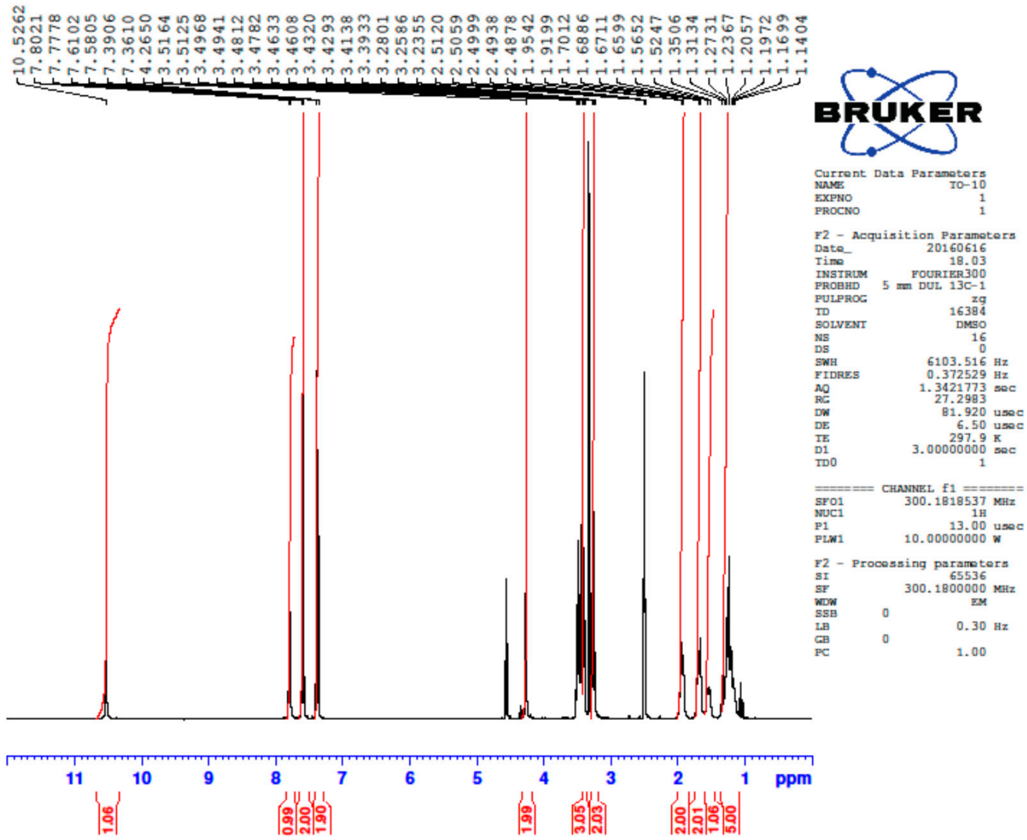

Figure 39. Compound 6j ^{1}H NMR spectrum

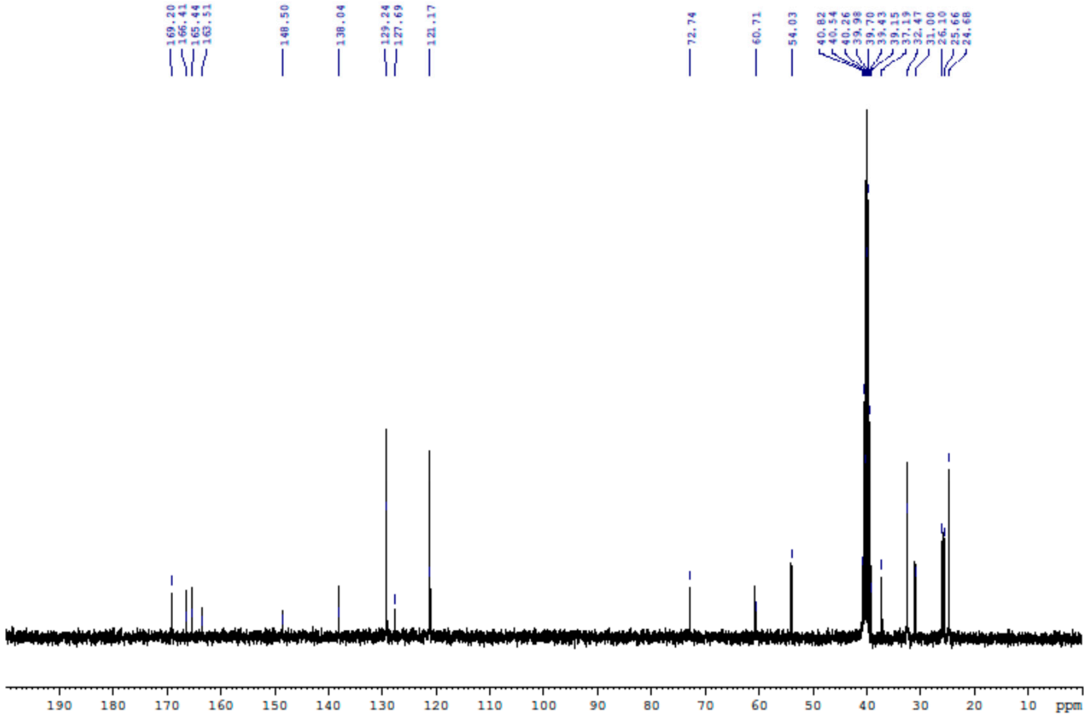

Figure 40. Compound 6j ^{13}C NMR spectrum

SHIMADZU

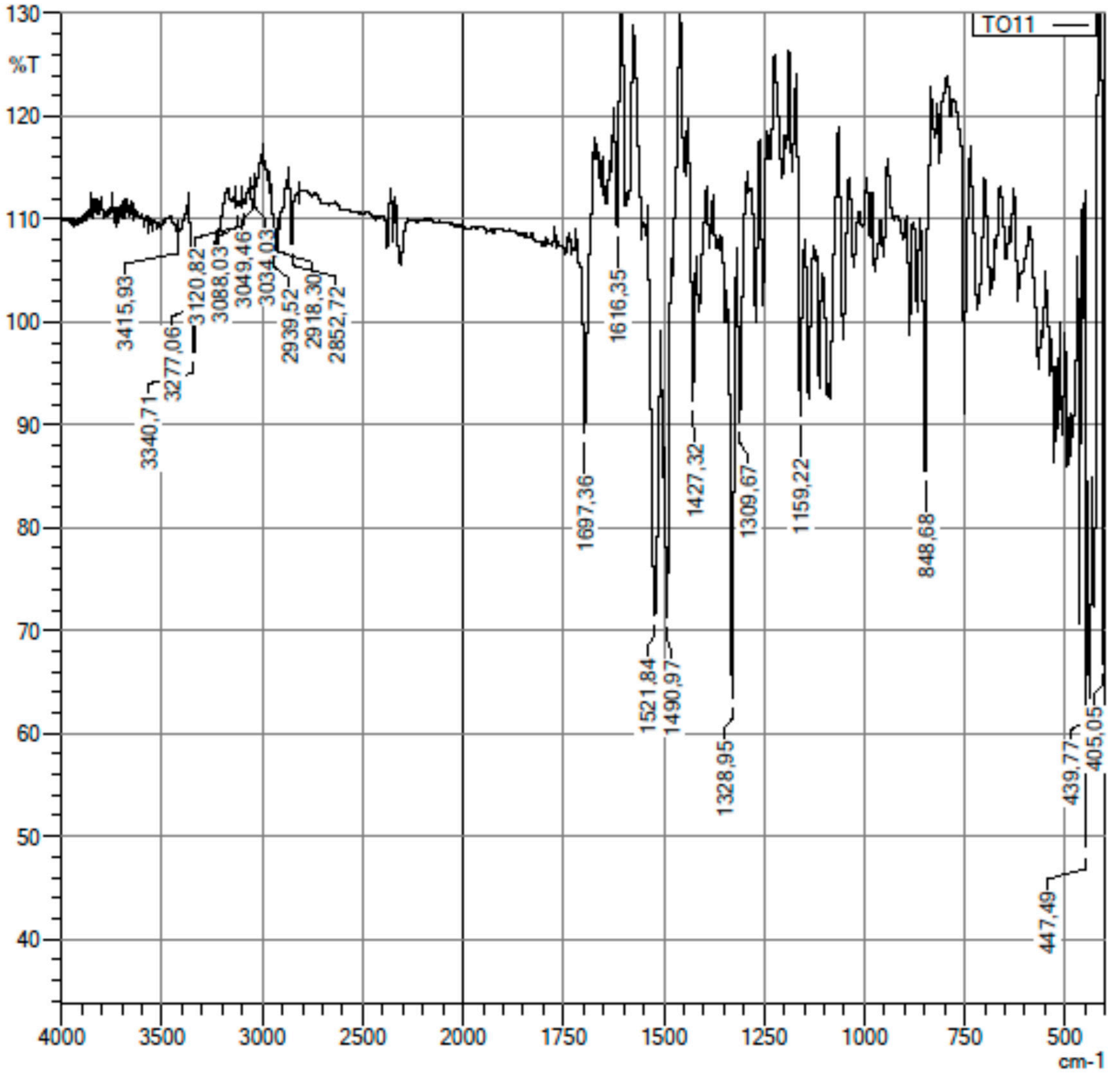

C:¥Users¥dopnalab¥Desktop¥BETアL¥TO¥TO11.ispd

	Item	Value
2	Sample name	TO-11
3	Sample ID	
4	Option	
5	Intensity Mode	%Transmittance
6	Apodization	Happ-Genzel
9	No. of Scans	10

Figure 41. Compound 6k IR spectrum

Data File: C:\LabSolutions\Data\Analiz\BKaya\TO11_13.lcd

Elmt	Val.	Min	Max	Elmt	Val.	Min	Max	Elmt	Val.	Min	Max	Use Adduct
H	1	10	40	O	2	2	5	Cl	1	0	0	H
C	4	10	26	F	1	0	0	Br	1	0	0	
N	3	7	7	S	2	3	3					

Error Margin (ppm): 5
HC Ratio: unlimited
Max Isotopes: 3
MSn Iso RI (%): 10.00

DBE Range: 9.0 - 19.0
Apply N Rule: yes
Isotope RI (%): 1.00
MSn Logic Mode: AND

Electron Ions: both
Use MSn Info: no
Isotope Res: 10000
Max Results: 500

Event#: 1 MS(E+) Ret. Time : 7.547 -> 7.573 Scan# : 1133 -> 1137

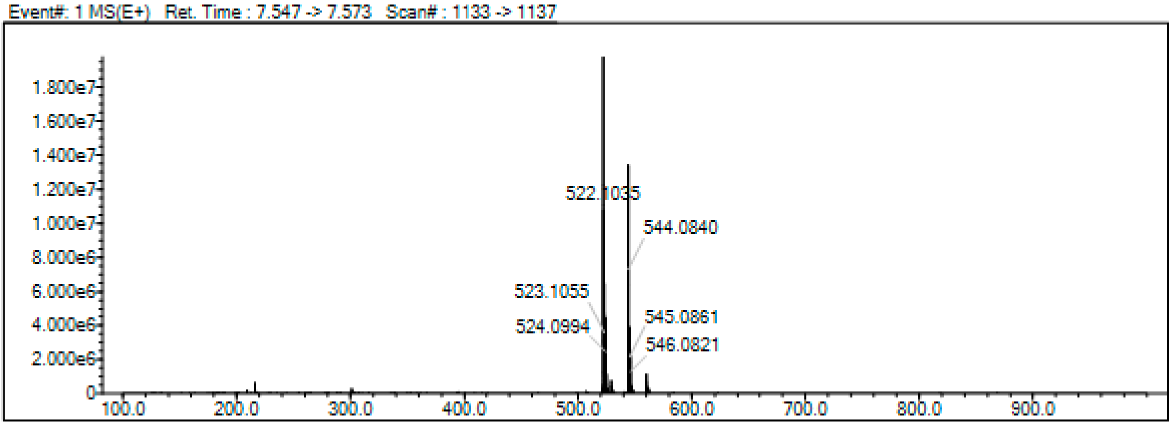

Measured region for 522.1035 m/z

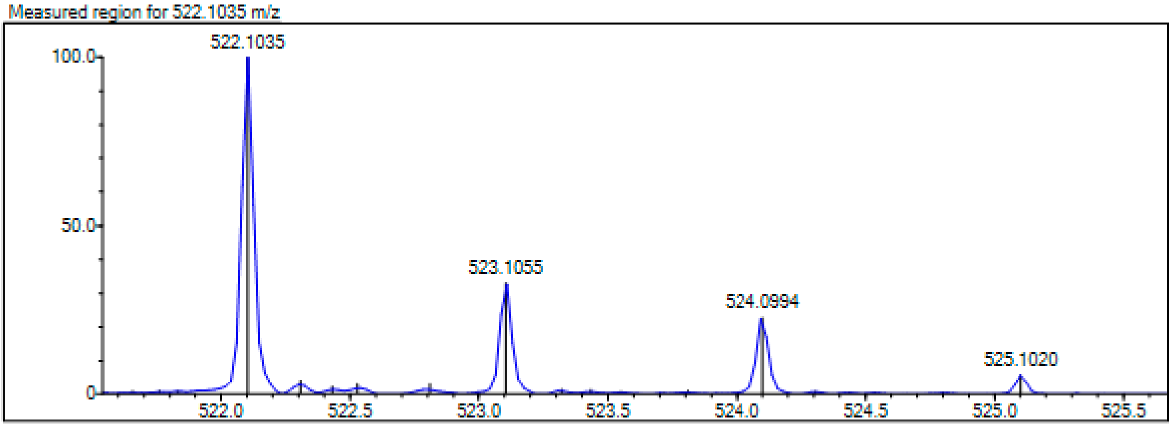

C20 H23 N7 O4 S3 [M+H]+ : Predicted region for 522.1046 m/z

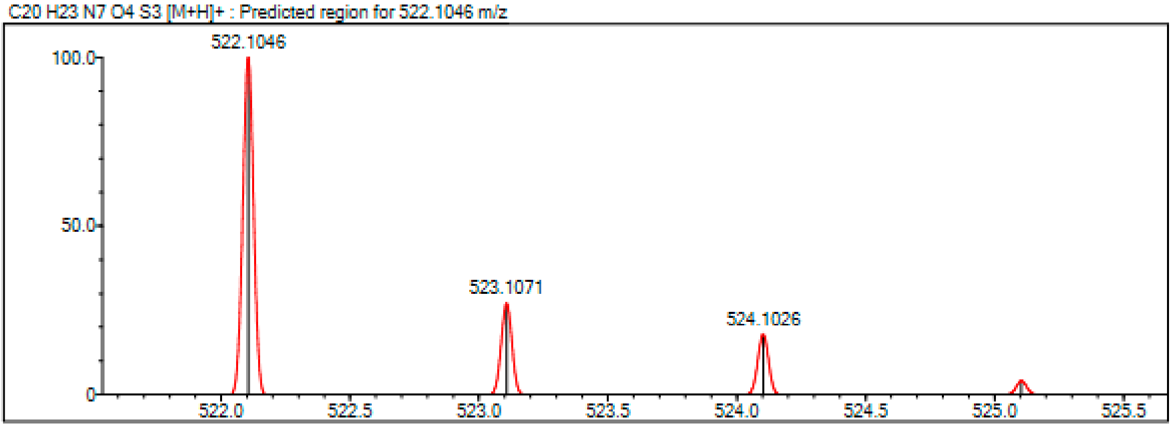

Rank	Score	Formula (M)	Ion	Meas. m/z	Pred. m/z	Df. (mDa)	Df. (ppm)	Iso	DBE
1	90.12	C20 H23 N7 O4 S3	[M+H]+	522.1035	522.1046	-1.1	-2.11	92.69	13.0

Figure 42. Compound 6k Mass spectrum

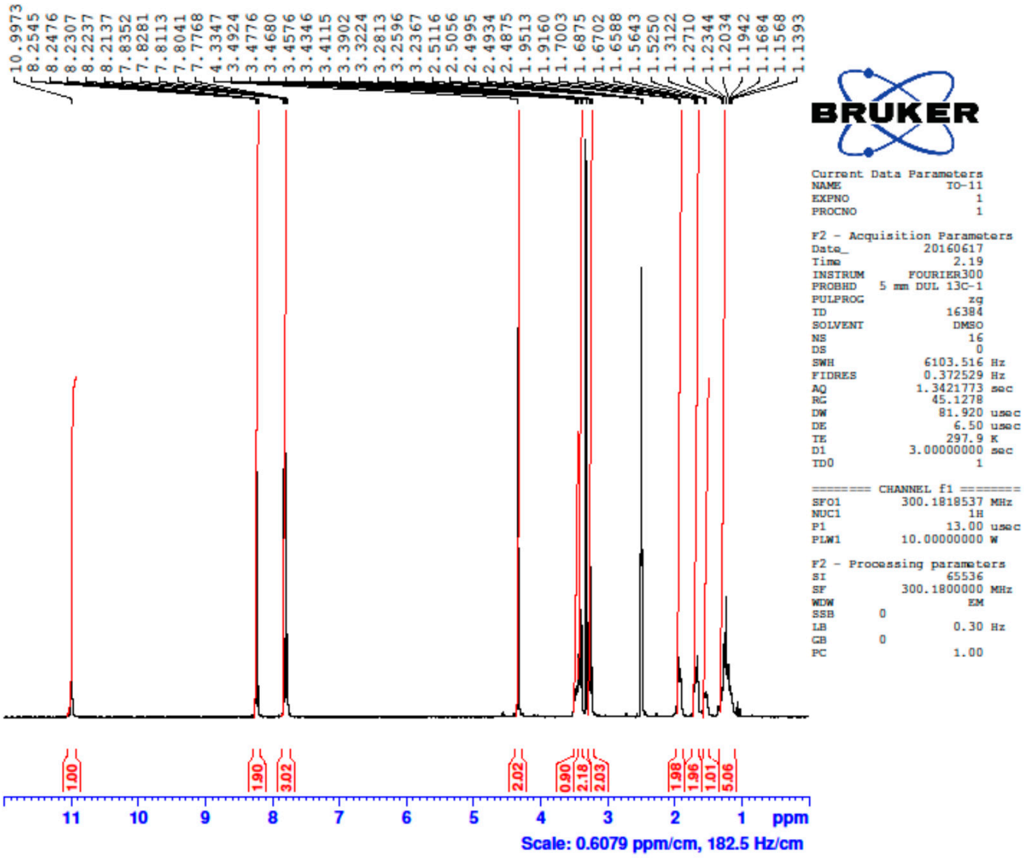

Figure 43. Compound 6k ^{1}H NMR spectrum

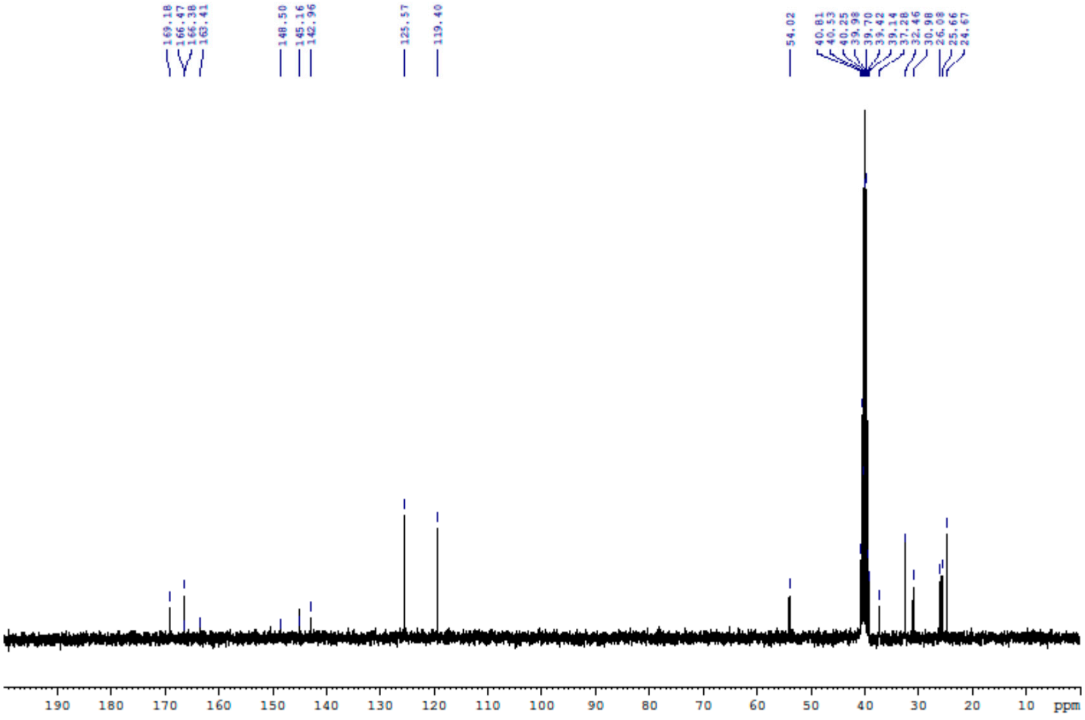

Figure 44. Compound 6k ^{13}C NMR spectrum

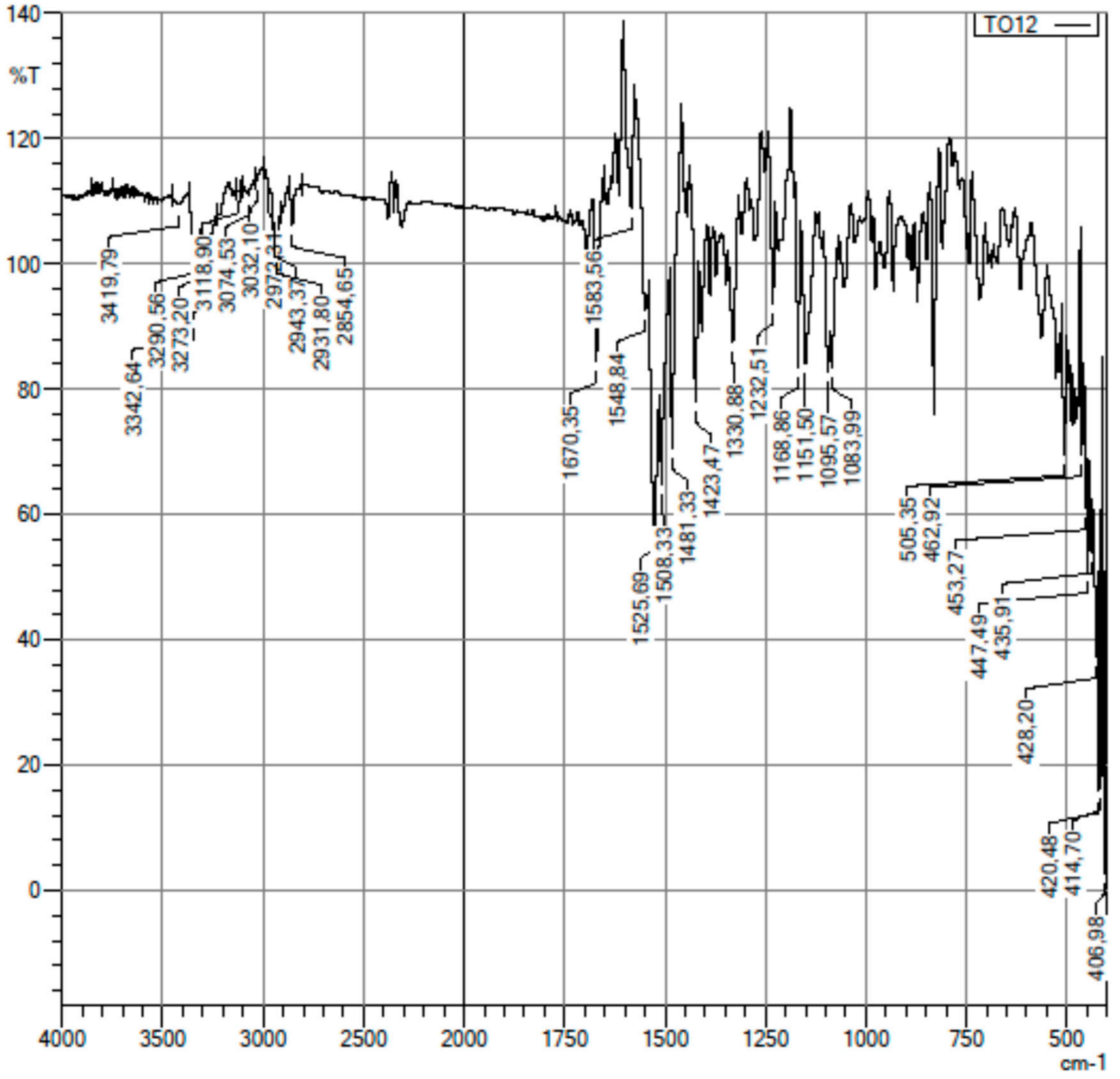

C:¥Users¥dopnalab¥Desktop¥BETワL¥TO¥TO12.ispd

	Item	Value
2	Sample name	TO-12
3	Sample ID	
4	Option	
5	Intensity Mode	%Transmittance
6	Apodization	Happ-Genzel
9	No. of Scans	10

Figure 45. Compound 6l IR spectrum

Data File: C:\LabSolutions\Data\Analiz\BKaya\TO12_14.lcd

Elmt	Val.	Min	Max	Elmt	Val.	Min	Max	Elmt	Val.	Min	Max	Use Adduct
H	1	10	40	O	2	2	5	Cl	1	0	0	H
C	4	10	26	F	1	1	1	Br	1	0	0	
N	3	6	7	S	2	3	3					

Error Margin (ppm): 5
HC Ratio: unlimited
Max Isotopes: 3
MSn Iso RI (%): 10.00

DBE Range: 9.0 - 19.0
Apply N Rule: yes
Isotope RI (%): 1.00
MSn Logic Mode: AND

Electron Ions: both
Use MSn Info: no
Isotope Res: 10000
Max Results: 500

Event#: 1 MS(E+) Ret. Time : 7.493 -> 7.520 Scan# : 1125 -> 1129

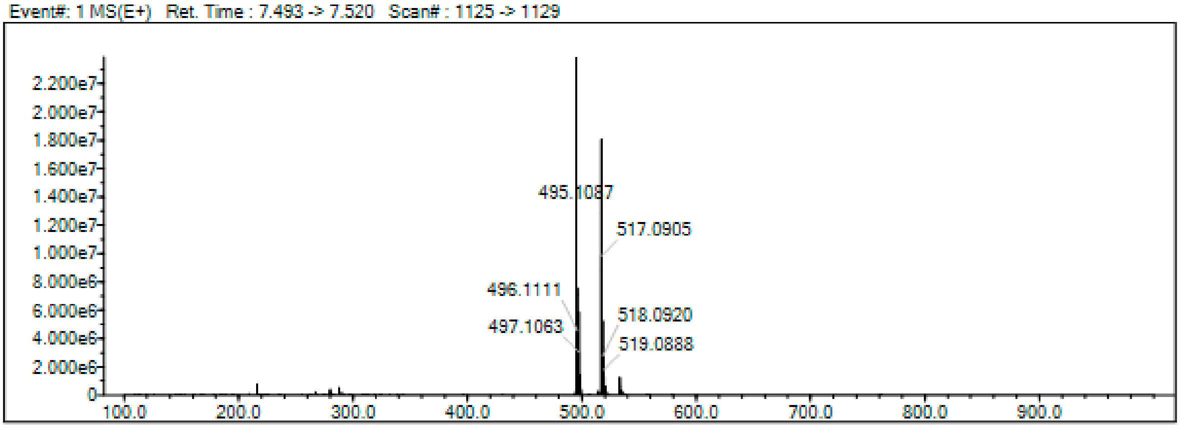

Measured region for 495.1087 m/z

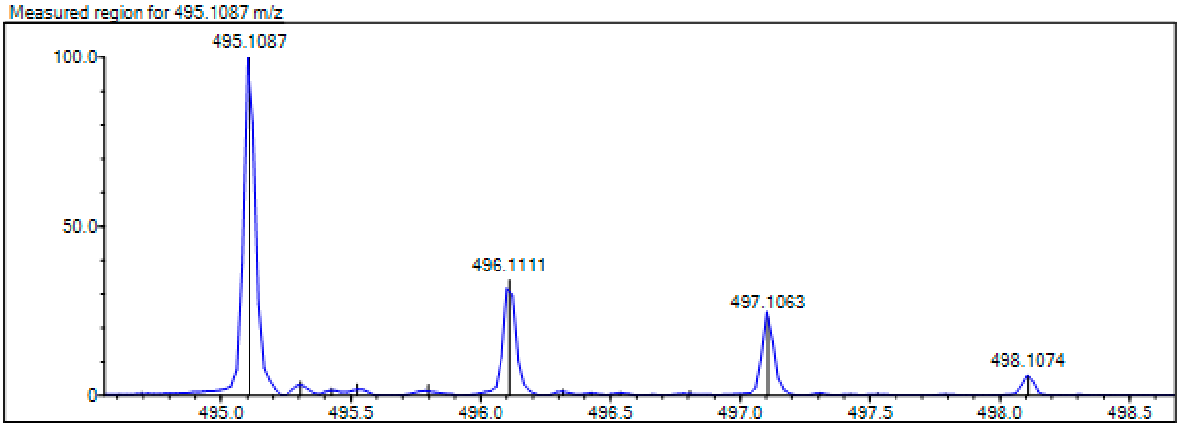

C20 H23 N6 O2 F S3 [M+H]+ : Predicted region for 495.1101 m/z

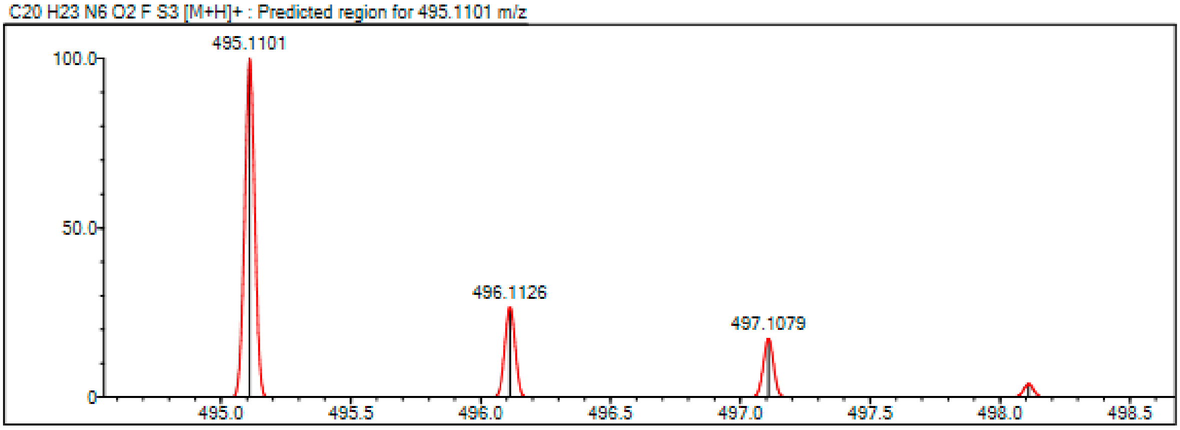

Rank	Score	Formula (M)	Ion	Meas. m/z	Pred. m/z	Df. (mDa)	Df. (ppm)	Iso	DBE
1	76.00	C20 H23 N6 O2 F S3	[M+H]+	495.1087	495.1101	-1.4	-2.83	79.64	12.0

Figure 46. Compound 6l Mass spectrum

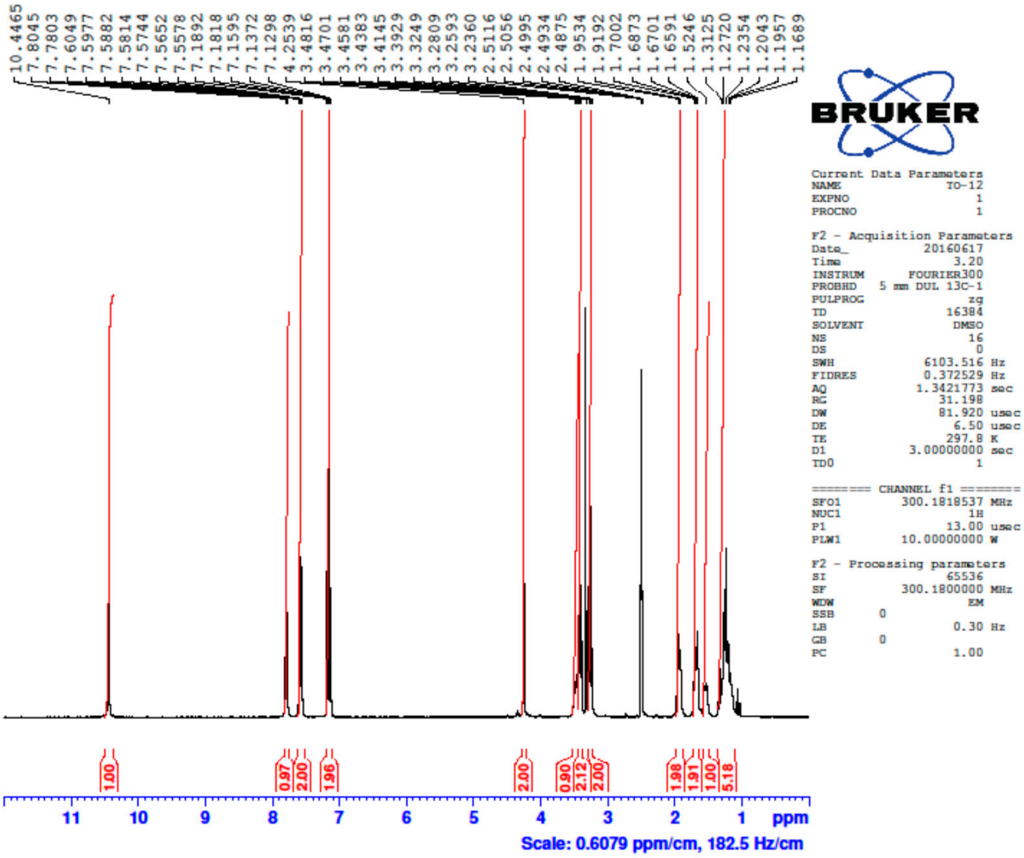

Figure 47. Compound 6l ^{1}H NMR spectrum

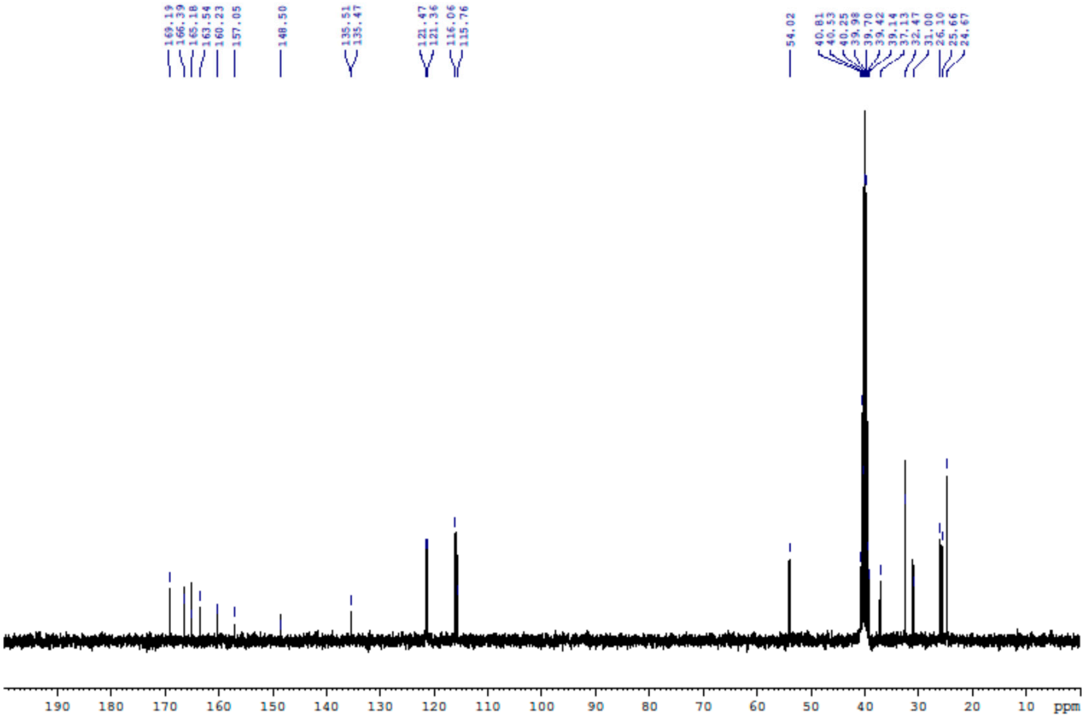

Figure 48. Compound 6l ^{13}C NMR spectrum

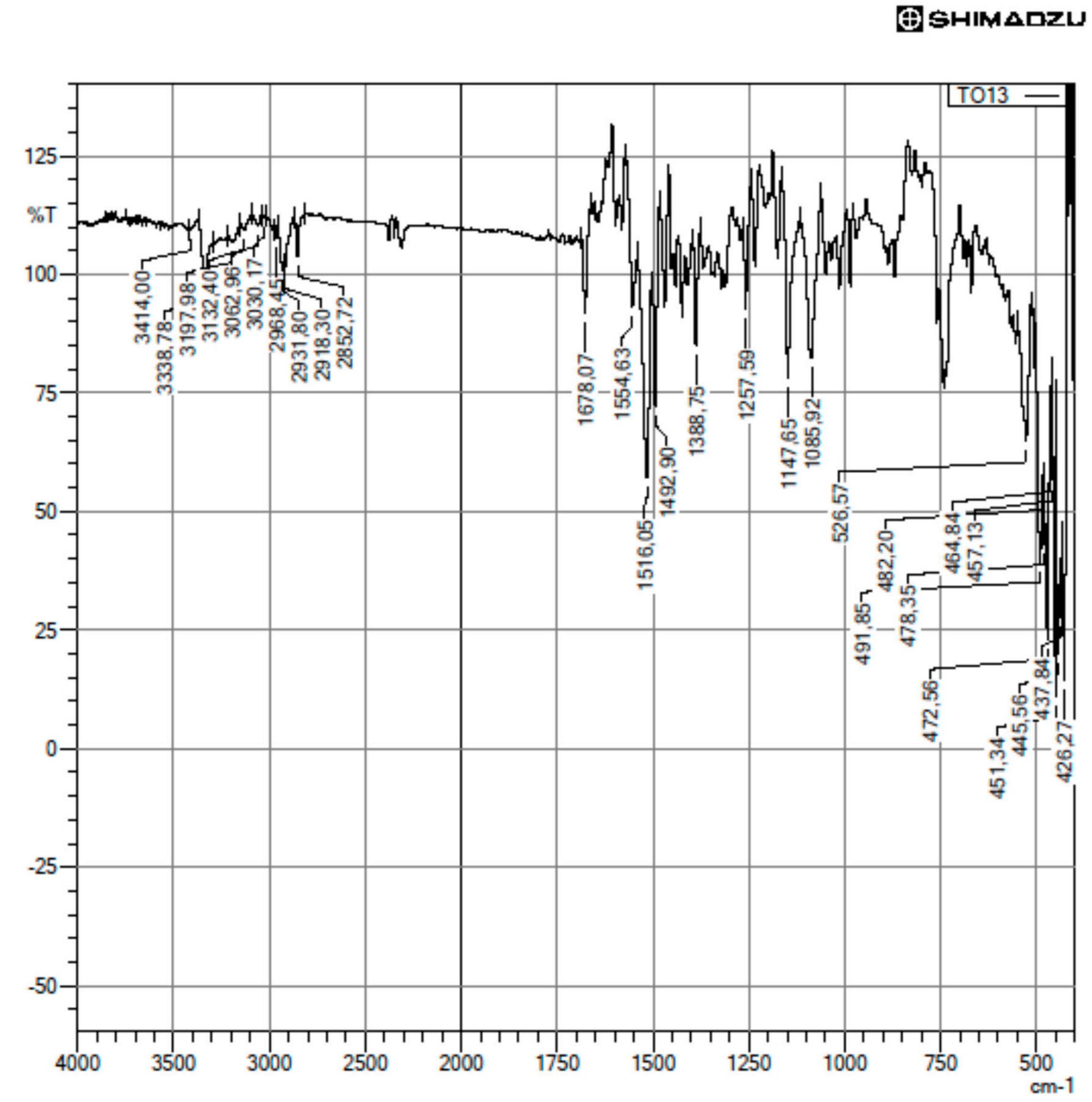

C:¥Users¥dopnalab¥Desktop¥BETワL¥TO¥TO13.ispd

	Item	Value
2	Sample name	TO-13
3	Sample ID	
4	Option	
5	Intensity Mode	%Transmittance
6	Apodization	Happ-Genzel
9	No. of Scans	10

Figure 49. Compound 6m IR spectrum

Formula Predictor Report - TO13_15.lcd Page 1 of 1

Data File: C:\LabSolutions\Data\Analiz\BKaya\TO13_15.lcd

Elmt	Val.	Min	Max	Elmt	Val.	Min	Max	Elmt	Val.	Min	Max	Use Adduct
H	1	10	40	O	2	2	5	Cl	1	0	0	H
C	4	10	26	F	1	0	0	Br	1	0	0	
N	3	7	7	S	2	4	4					

Error Margin (ppm): 5
HC Ratio: unlimited
Max Isotopes: 3
MSn Iso RI (%): 10.00

DBE Range: 9.0 - 19.0
Apply N Rule: yes
Isotope RI (%): 1.00
MSn Logic Mode: AND

Electron Ions: both
Use MSn Info: no
Isotope Res: 10000
Max Results: 500

Event#: 1 MS(E+) Ret. Time : 7.720 -> 7.907 Scan# : 1159 -> 1187

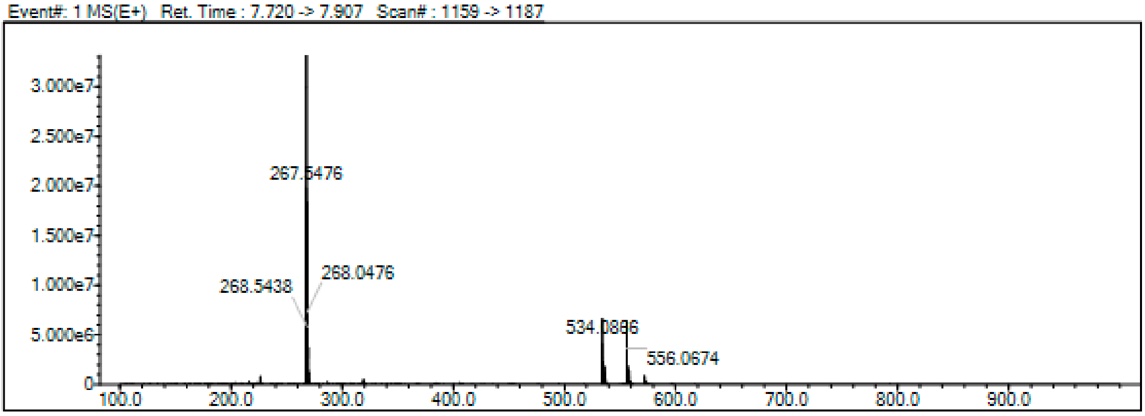

Measured region for 534.0866 m/z

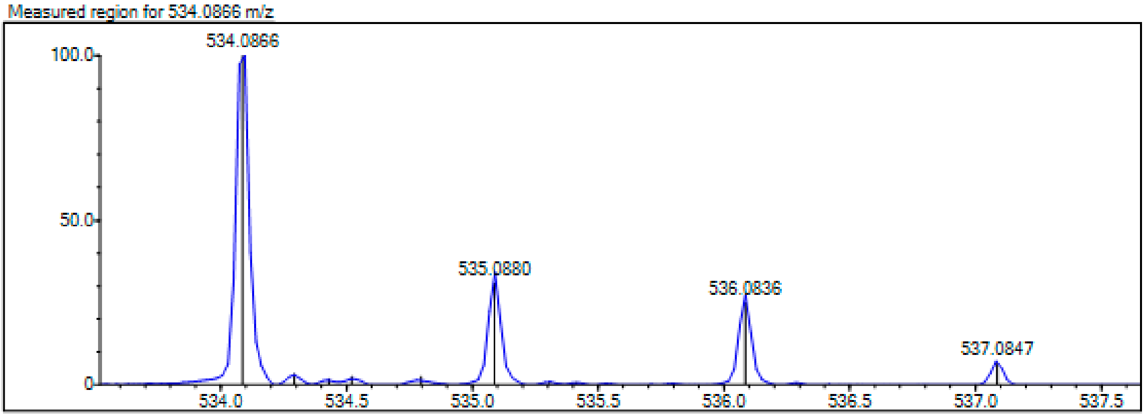

C21 H23 N7 O2 S4 [M+H]+ : Predicted region for 534.0869 m/z

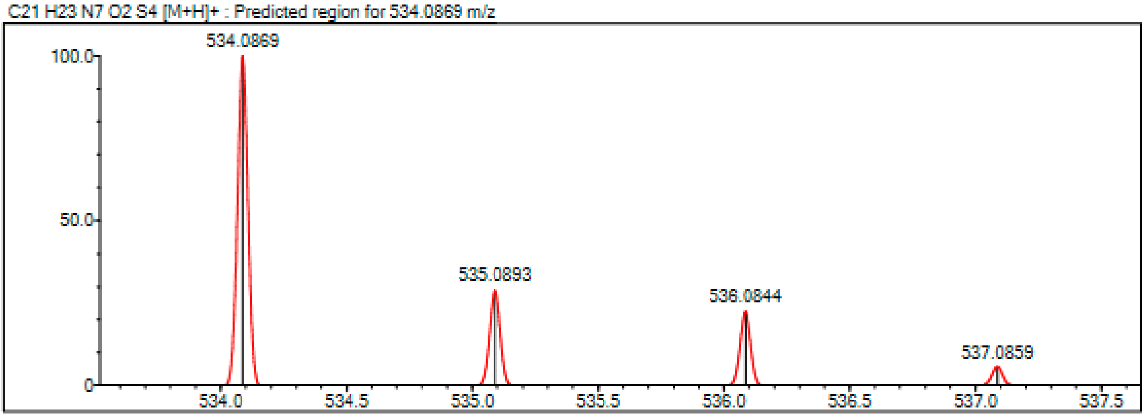

Rank	Score	Formula (M)	Ion	Meas. m/z	Pred. m/z	Df. (mDa)	Df. (ppm)	Iso	DBE
1	86.50	C21 H23 N7 O2 S4	[M+H]+	534.0866	534.0869	-0.3	-0.56	86.50	14.0

Figure 50. Compound 6m Mass spectrum

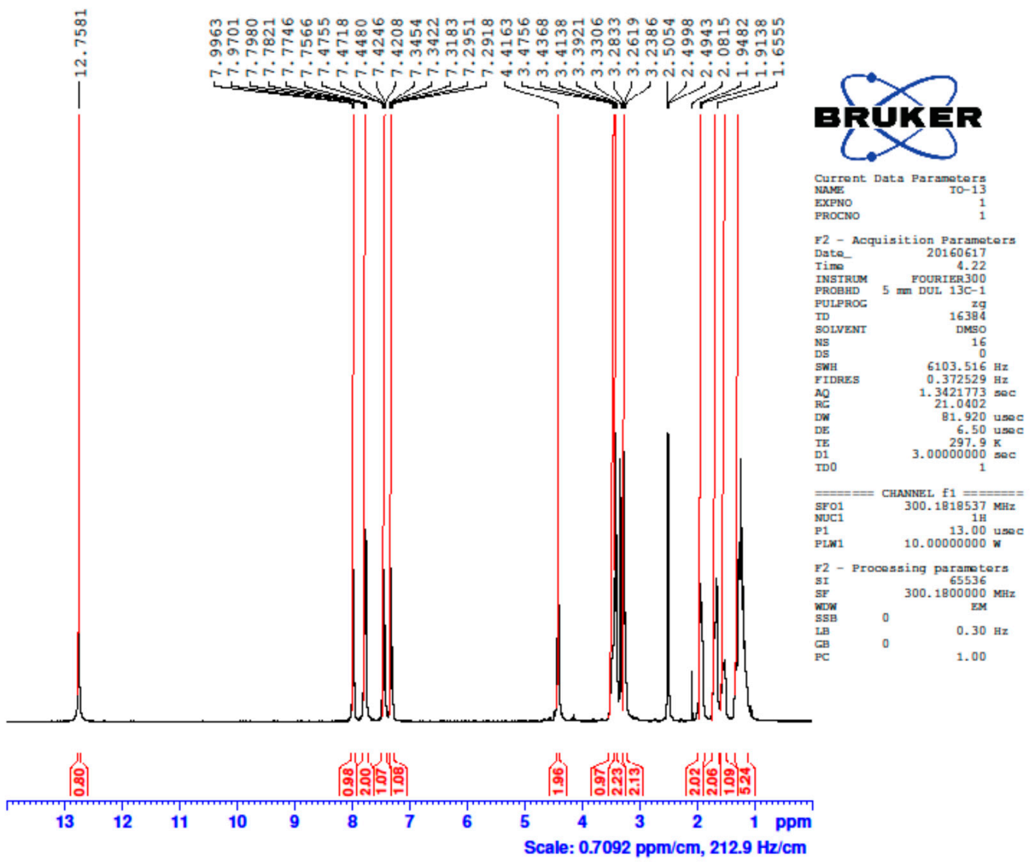

Figure 51. Compound 6m ^{1}H NMR spectrum

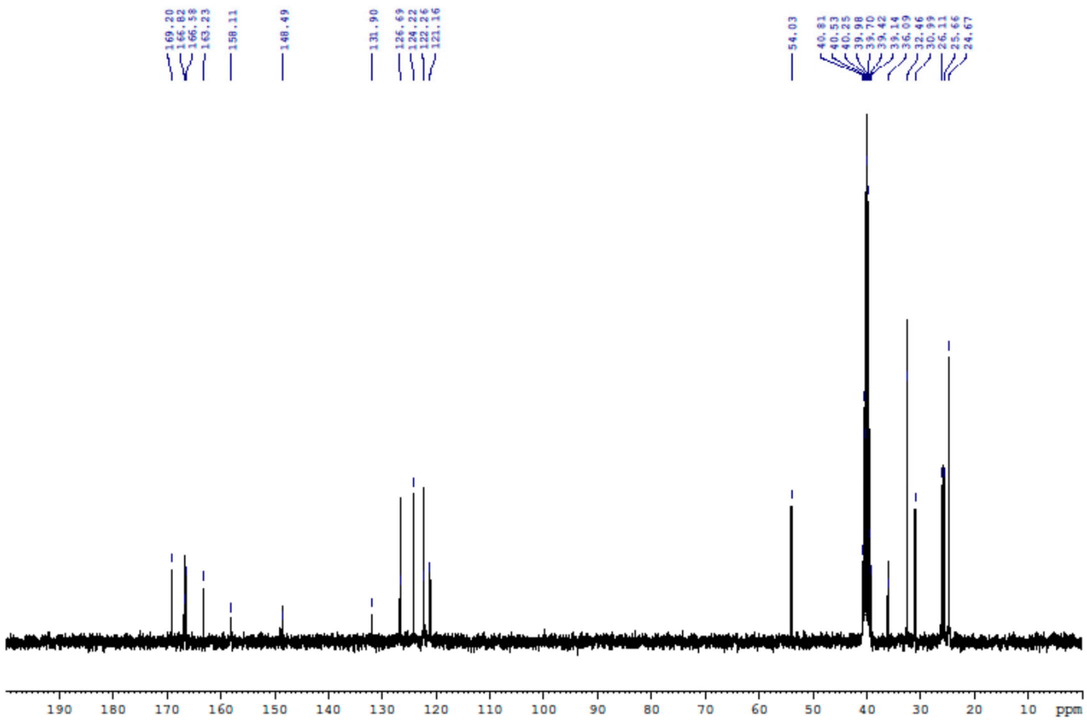

Figure 52. Compound 6m ^{13}C NMR spectrum

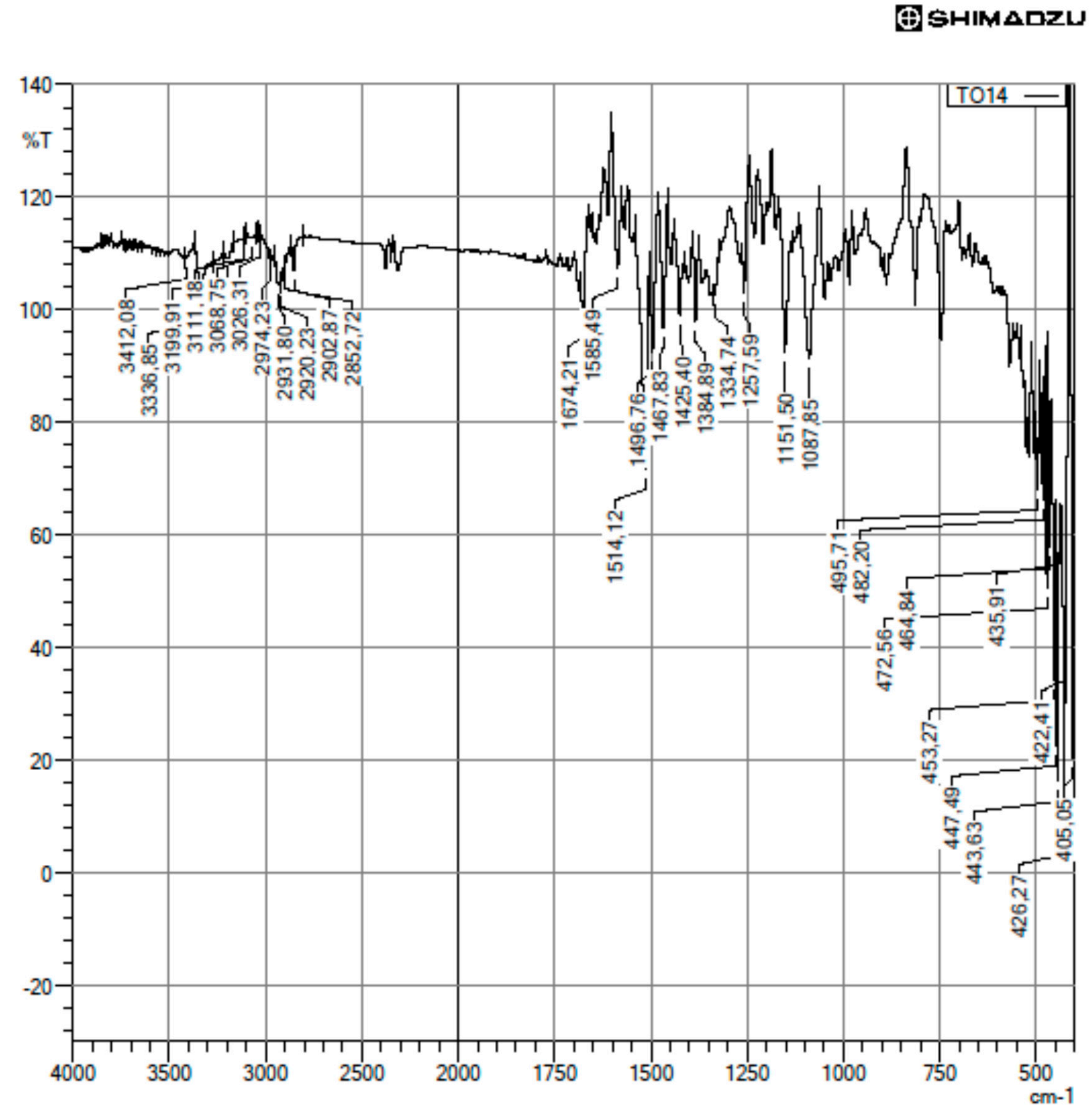

C:¥Users¥dopnalab¥Desktop¥BET7L¥TO¥TO14.ispd

	Item	Value
2	Sample name	TO-14
3	Sample ID	
4	Option	
5	Intensity Mode	%Transmittance
6	Apodization	Happ-Genzel
9	No. of Scans	10

1

Figure 53. Compound 6n IR spectrum

Data File: C:\LabSolutions\Data\Analiz\BKaya\TO14_16.lcd

Elmt	Val.	Min	Max	Elmt	Val.	Min	Max	Elmt	Val.	Min	Max	Use Adduct
H	1	10	40	O	2	2	5	Cl	1	0	0	H
C	4	10	26	F	1	0	0	Br	1	0	0	
N	3	7	7	S	2	4	4					

Error Margin (ppm):	5	DBE Range:	9.0 - 19.0	Electron Ions:	both
HC Ratio:	unlimited	Apply N Rule:	yes	Use MSn Info:	no
Max Isotopes:	3	Isotope RI (%):	1.00	Isotope Res:	10000
MSn Iso RI (%):	10.00	MSn Logic Mode:	AND	Max Results:	500

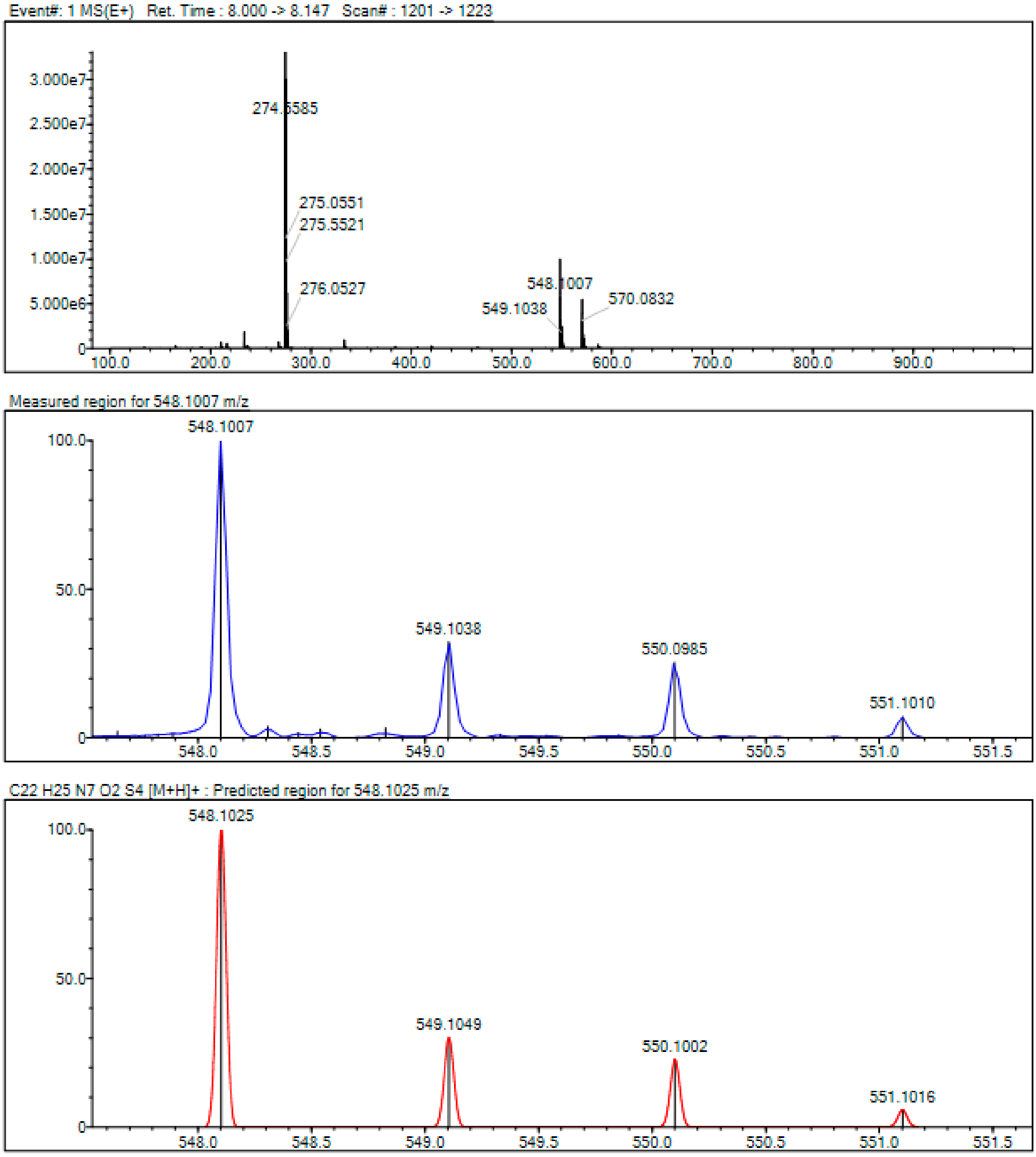

Rank	Score	Formula (M)	Ion	Meas. m/z	Pred. m/z	Df. (mDa)	Df. (ppm)	Iso	DBE
1	91.18	C22 H25 N7 O2 S4	[M+H]+	548.1007	548.1025	-1.8	-3.28	96.69	14.0

Figure 54. Compound 6n Mass spectrum

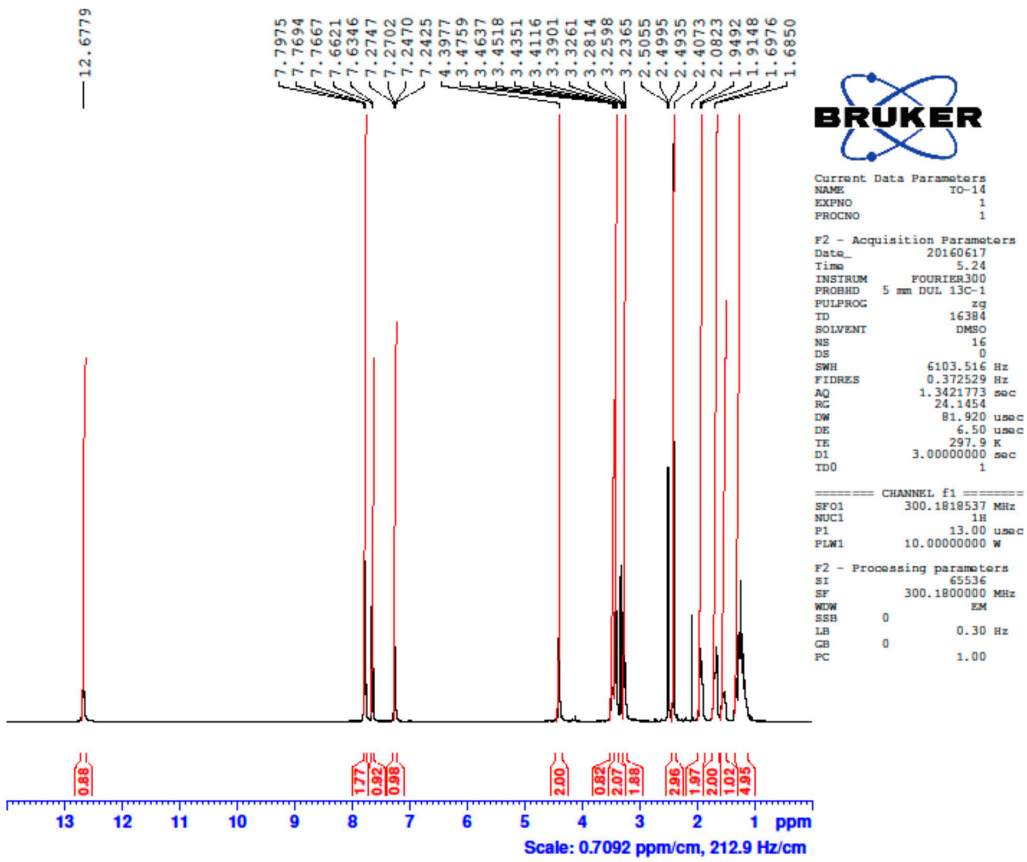

Figure 55. Compound 6n ^{1}H NMR spectrum

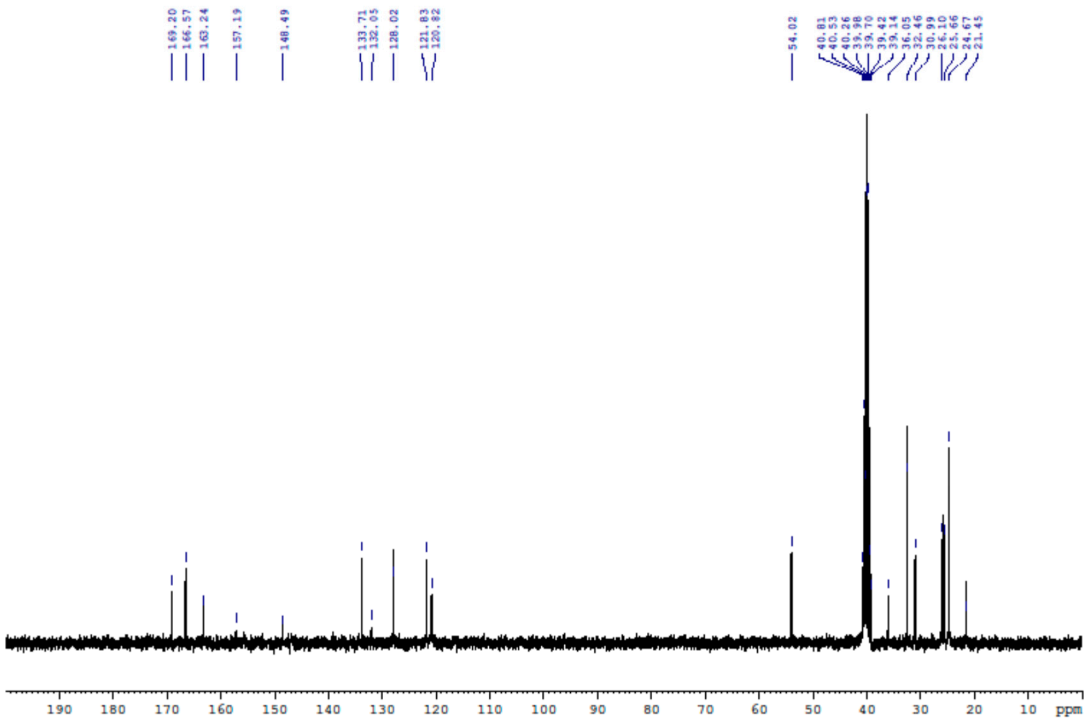

Figure 56. Compound 6n ^{13}C NMR spectrum

SHIMADZU

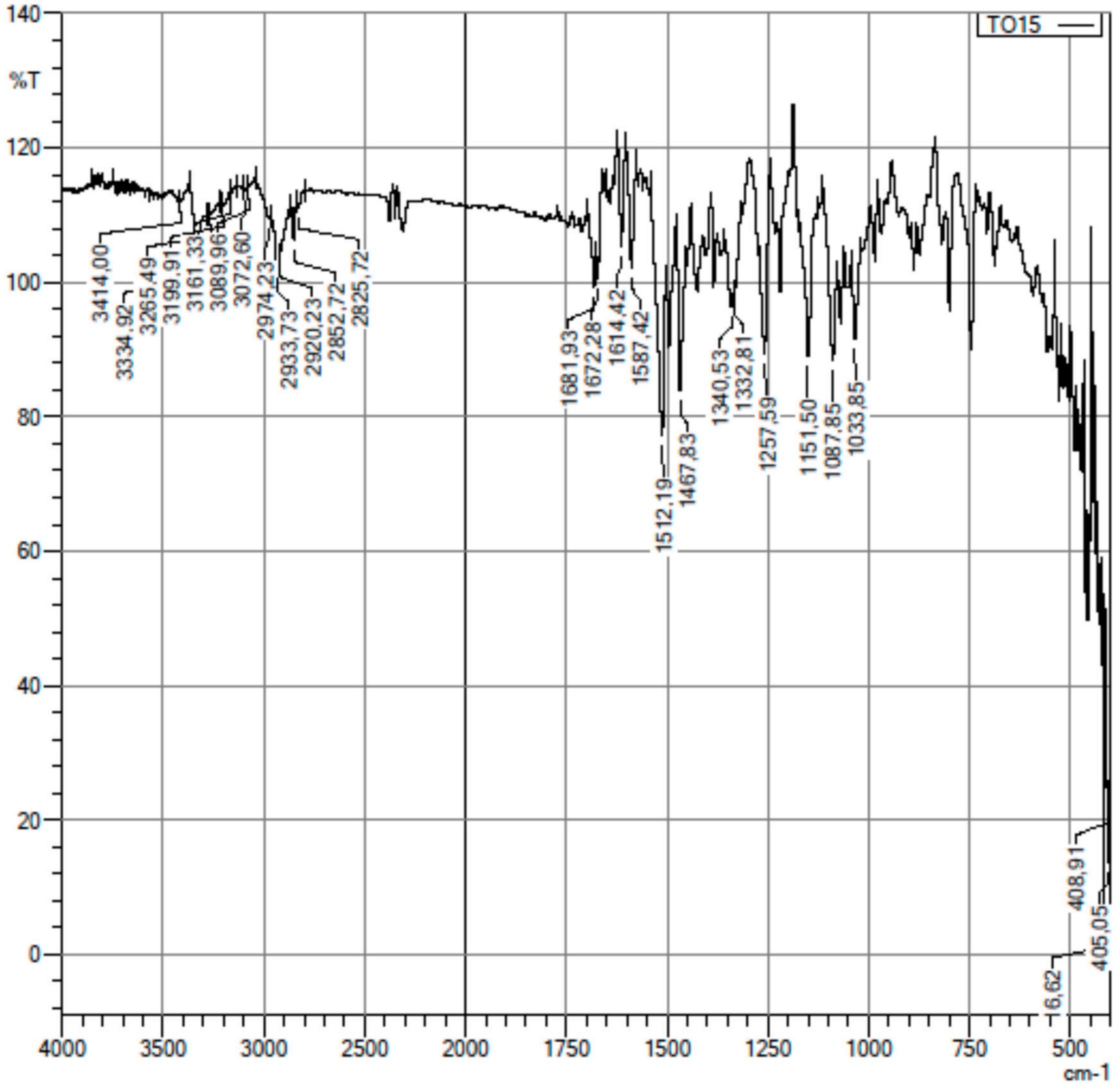

C:¥Users¥dopnalab¥Desktop¥BET7L¥TO¥TO15.ispd

	Item	Value
2	Sample name	TO-15
3	Sample ID	
4	Option	
5	Intensity Mode	%Transmittance
6	Apodization	Happ-Genzel
9	No. of Scans	10

1

Figure 57. Compound 6o IR spectrum

Data File: C:\LabSolutions\Data\Analiz\BKaya\TO15_17.lcd

Elmt	Val.	Min	Max	Elmt	Val.	Min	Max	Elmt	Val.	Min	Max	Use Adduct
H	1	10	40	O	2	2	5	Cl	1	0	0	H
C	4	10	26	F	1	0	0	Br	1	0	0	
N	3	7	7	S	2	4	4					

Error Margin (ppm):	5	DBE Range:	9.0 - 19.0	Electron Ions:	both
HC Ratio:	unlimited	Apply N Rule:	yes	Use MSn Info:	no
Max Isotopes:	3	Isotope RI (%):	1.00	Isotope Res:	10000
MSn Iso RI (%):	10.00	MSn Logic Mode:	AND	Max Results:	500

Event#: 1 MS(E+) Ret. Time : 7.653 -> 7.693 Scan# : 1149 -> 1155

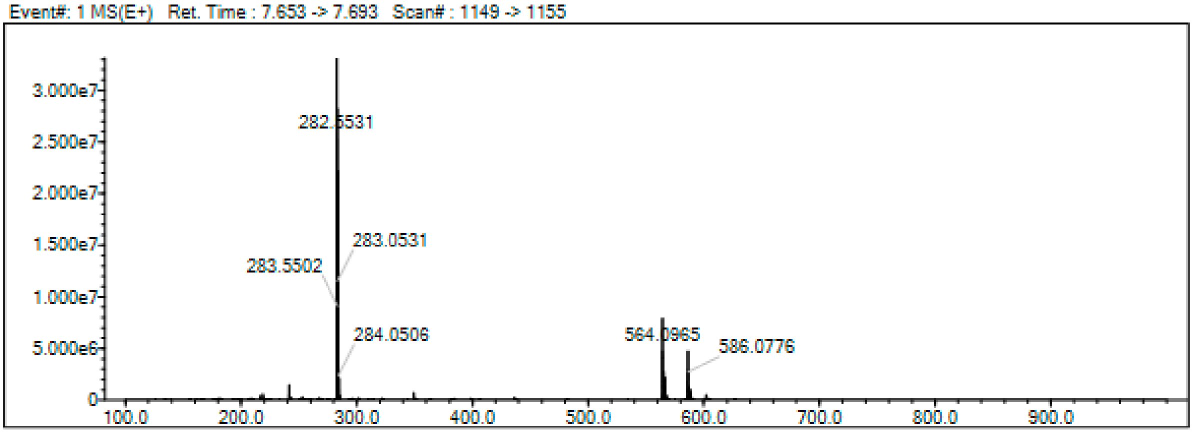

Measured region for 564.0965 m/z

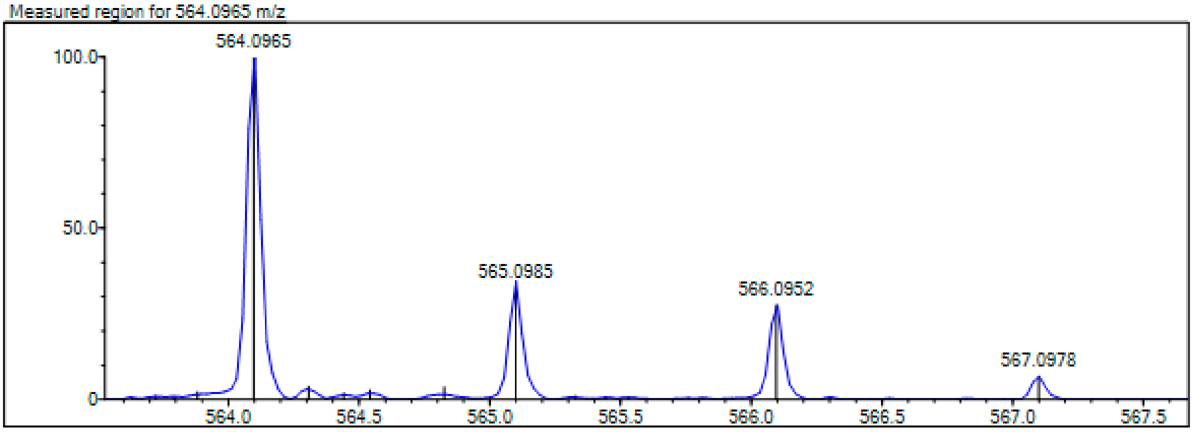

C22 H25 N7 O3 S4 [M+H]+ : Predicted region for 564.0974 m/z

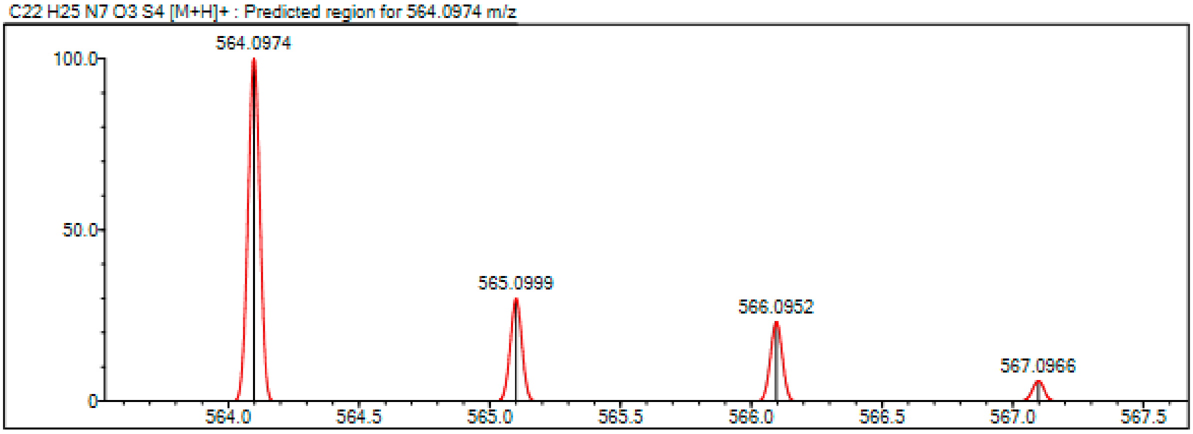

Rank	Score	Formula (M)	Ion	Meas. m/z	Pred. m/z	Df. (mDa)	Df. (ppm)	Iso	DBE
1	90.08	C22 H25 N7 O3 S4	[M+H]+	564.0965	564.0974	-0.9	-1.60	91.45	14.0

Figure 58. Compound 6o Mass spectrum

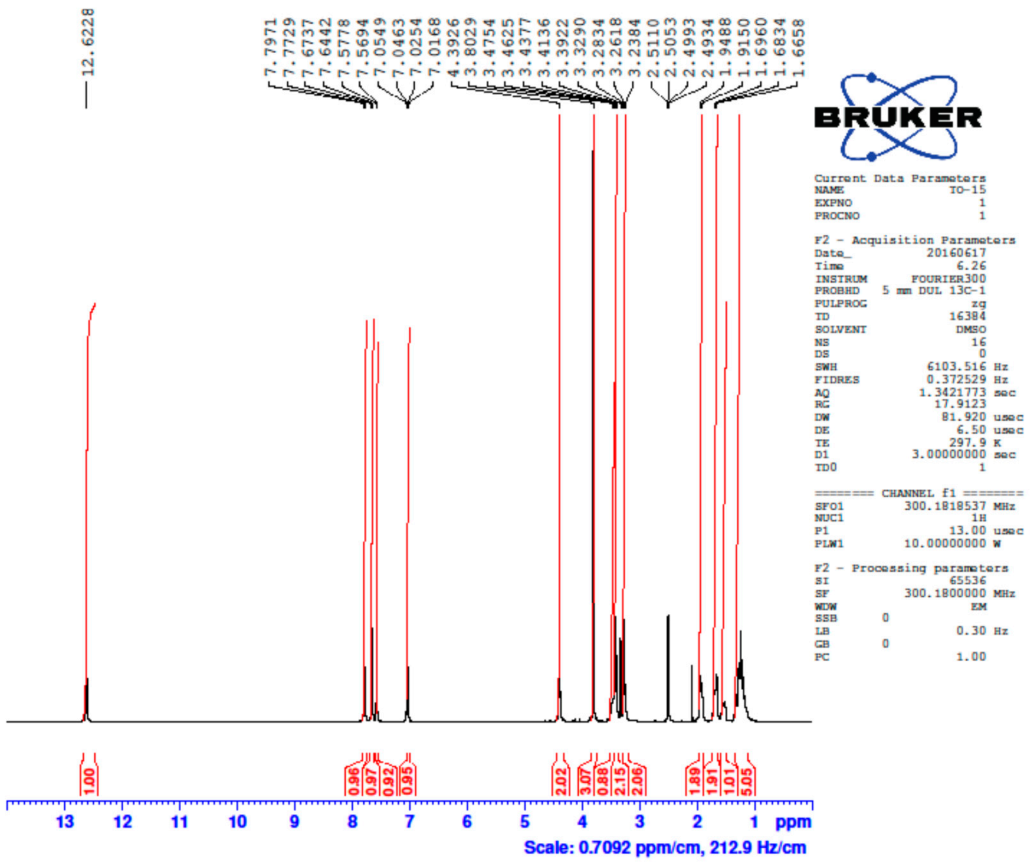

Figure 59. Compound 6o ^{1}H NMR spectrum

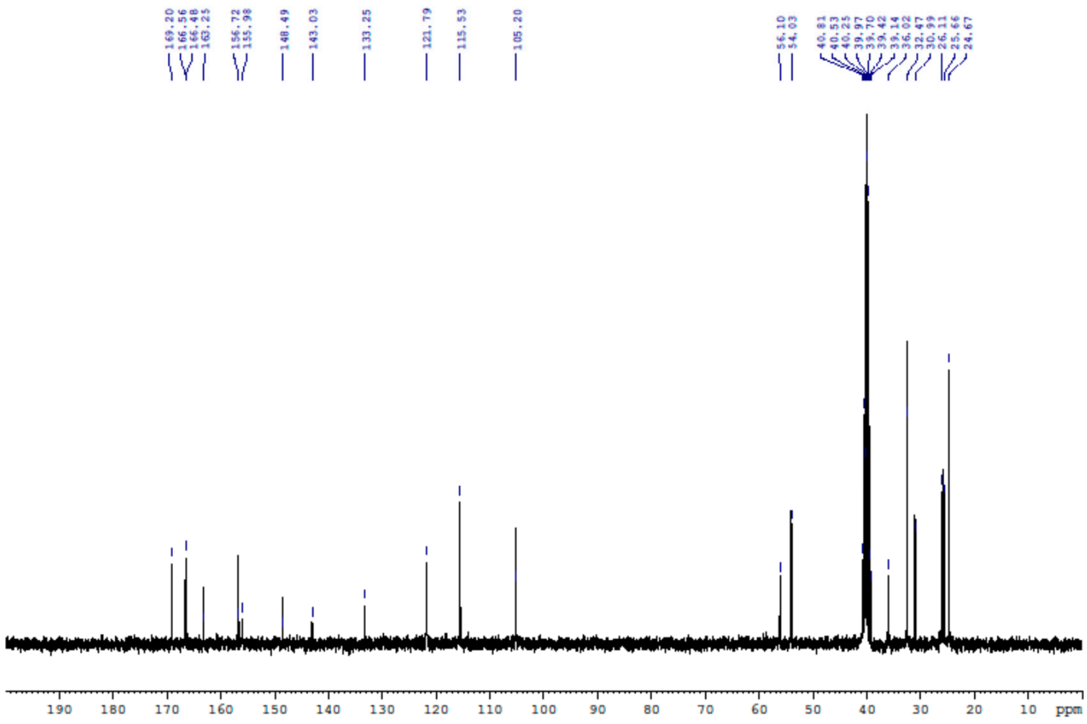

Figure 60. Compound 6o ^{13}C NMR spectrum

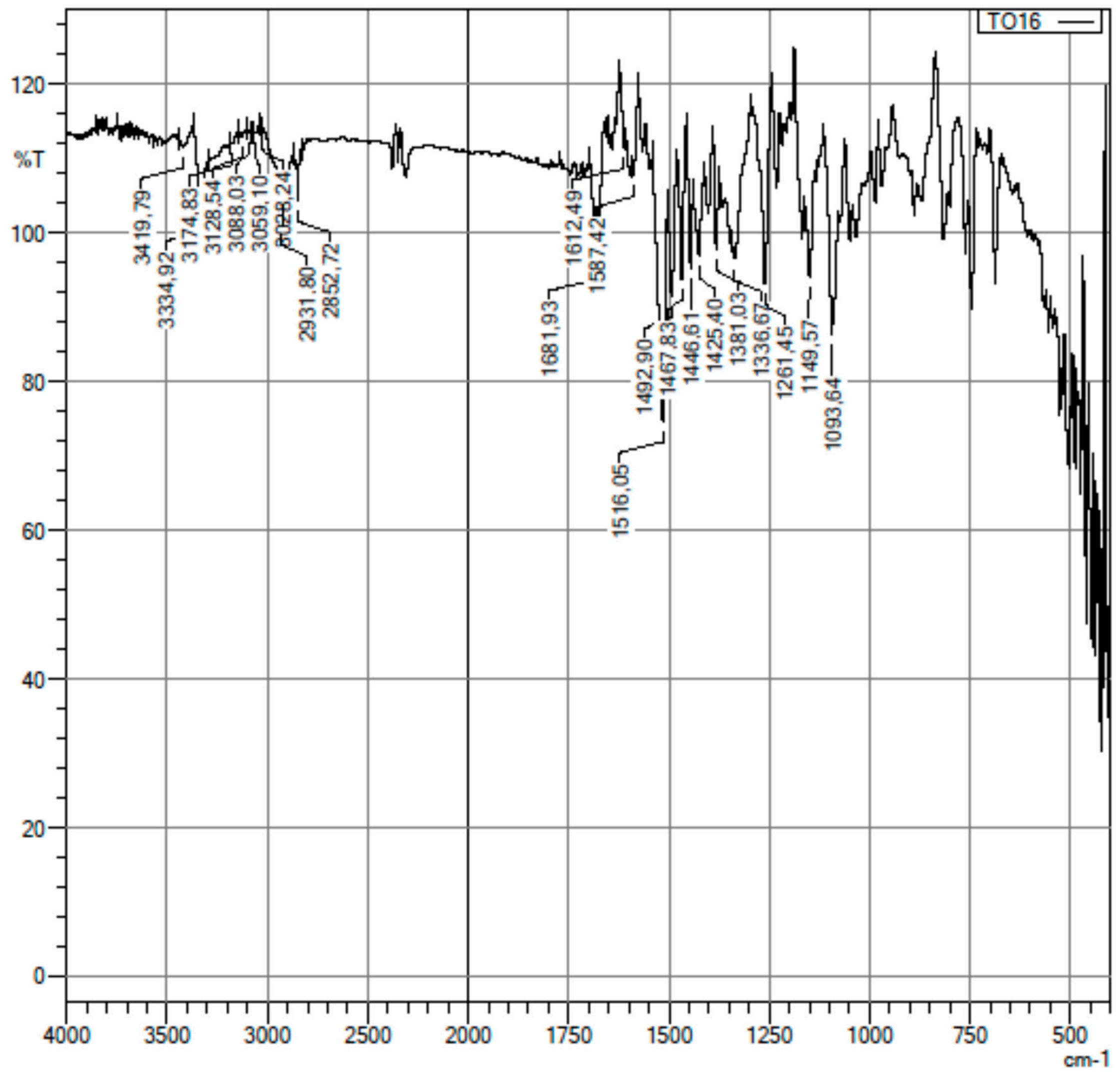

C:¥Users¥dopnalab¥Desktop¥BET7L¥TO¥TO16.ispd

	Item	Value
2	Sample name	TO-16
3	Sample ID	
4	Option	
5	Intensity Mode	%Transmittance
6	Apodization	Happ-Genzel
9	No. of Scans	10

1

Figure 61. Compound 6p IR spectrum

Data File: C:\LabSolutions\Data\Analiz\BKaya\TO16_18.lcd

Elmt	Val.	Min	Max	Elmt	Val.	Min	Max	Elmt	Val.	Min	Max	Use Adduct
H	1	10	40	O	2	2	5	Cl	1	1	1	H
C	4	10	26	F	1	0	0	Br	1	0	0	
N	3	7	7	S	2	4	4					

Error Margin (ppm):	5	DBE Range:	9.0 - 19.0	Electron Ions:	both
HC Ratio:	unlimited	Apply N Rule:	yes	Use MSn Info:	no
Max Isotopes:	3	Isotope RI (%):	1.00	Isotope Res:	10000
MSn Iso RI (%):	10.00	MSn Logic Mode:	AND	Max Results:	500

Event#: 1 MS(E+) Ret. Time : 8.853 -> 8.960 Scan# : 1329 -> 1345

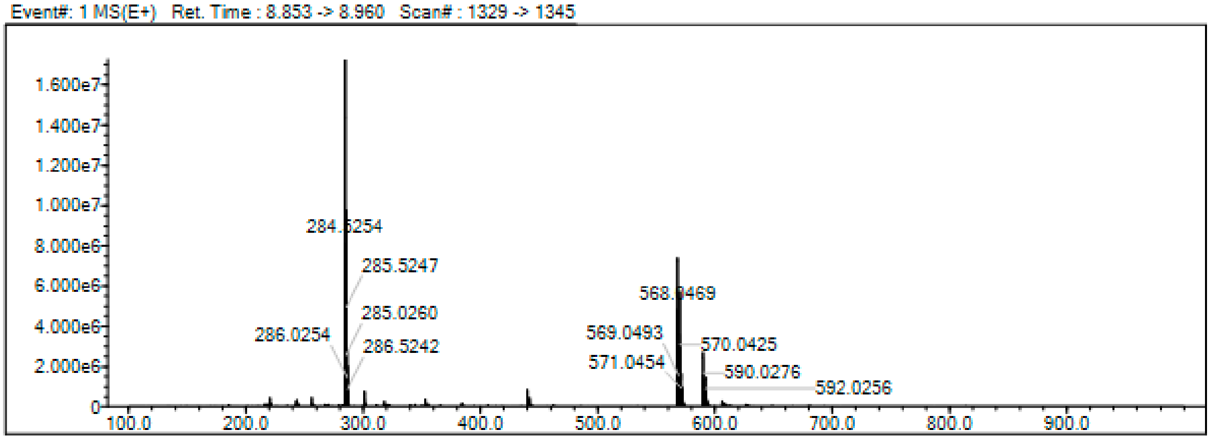

Measured region for 568.0469 m/z

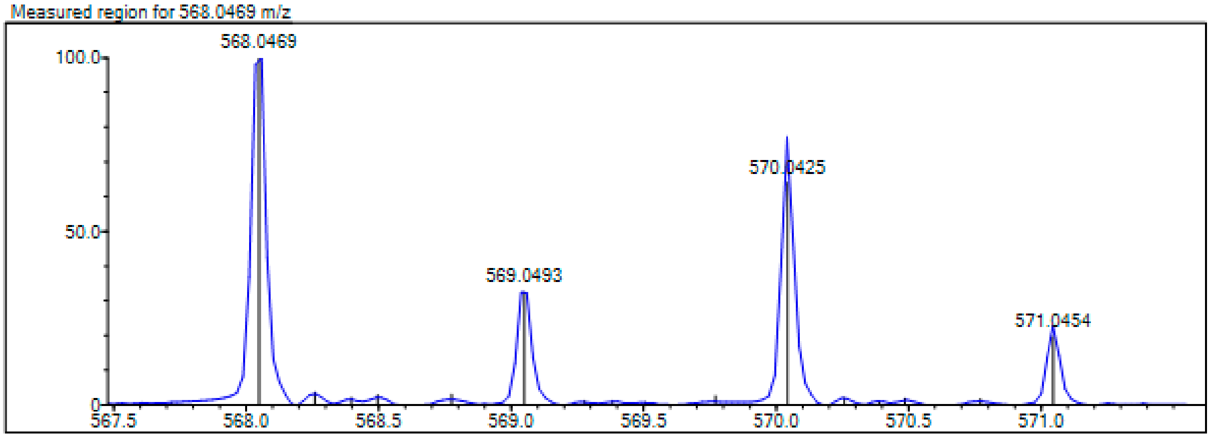

C21 H22 N7 O2 S4 Cl [M+H]+ : Predicted region for 568.0479 m/z

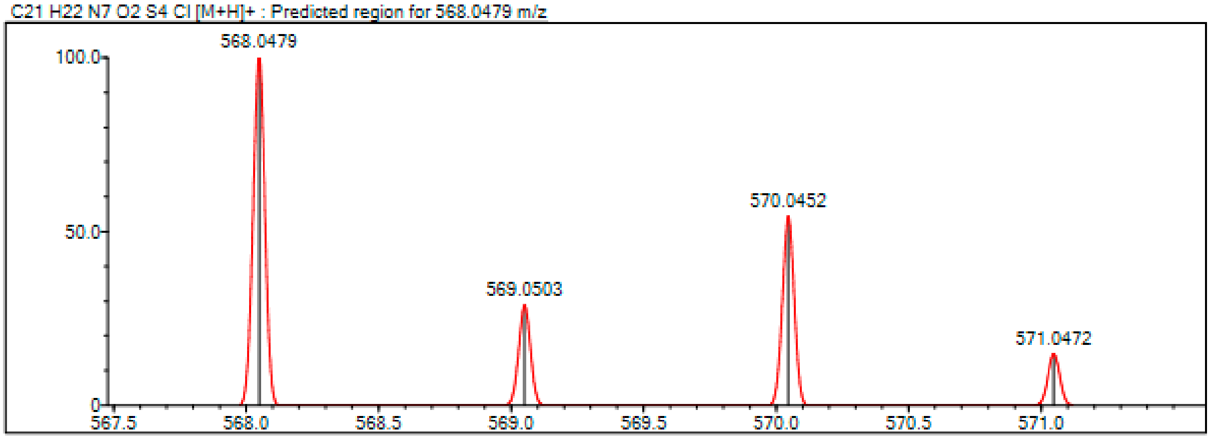

Rank	Score	Formula (M)	Ion	Meas. m/z	Pred. m/z	Df. (mDa)	Df. (ppm)	Iso	DBE
1	63.17	C21 H22 N7 O2 S4 Cl	[M+H]+	568.0469	568.0479	-1.0	-1.76	64.40	14.0

Figure 62. Compound 6p Mass spectrum

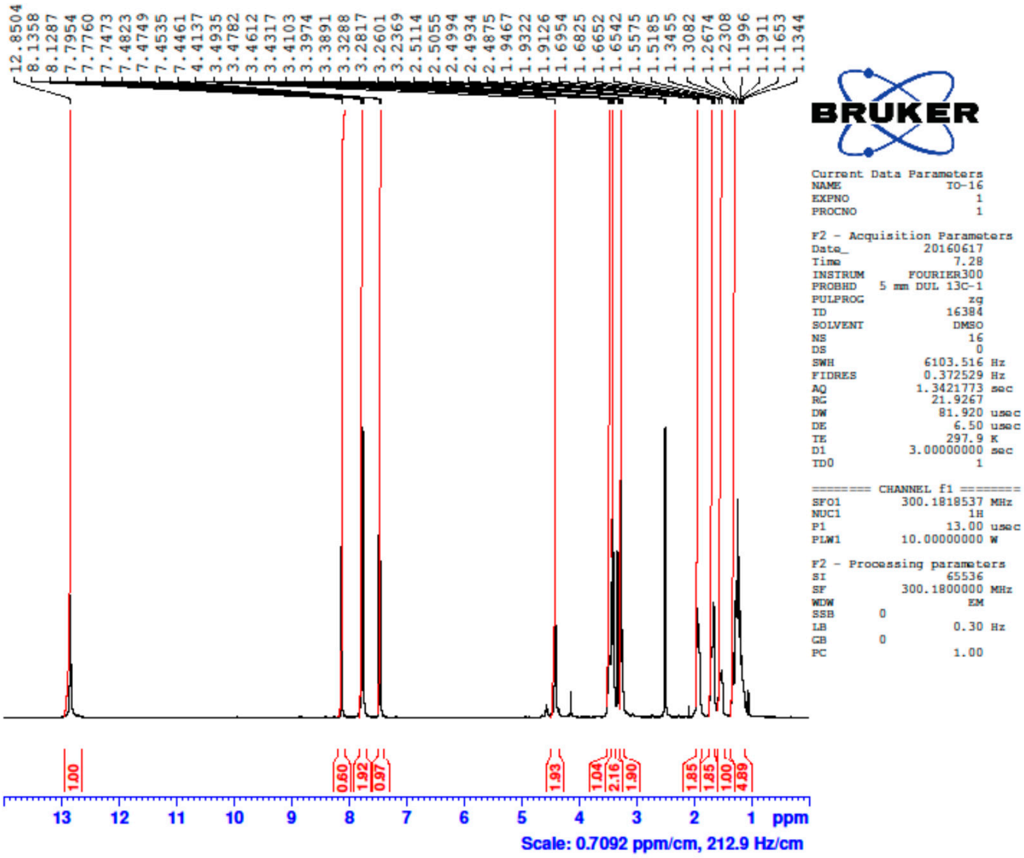

Figure 63. Compound 6p ^{1}H NMR spectrum

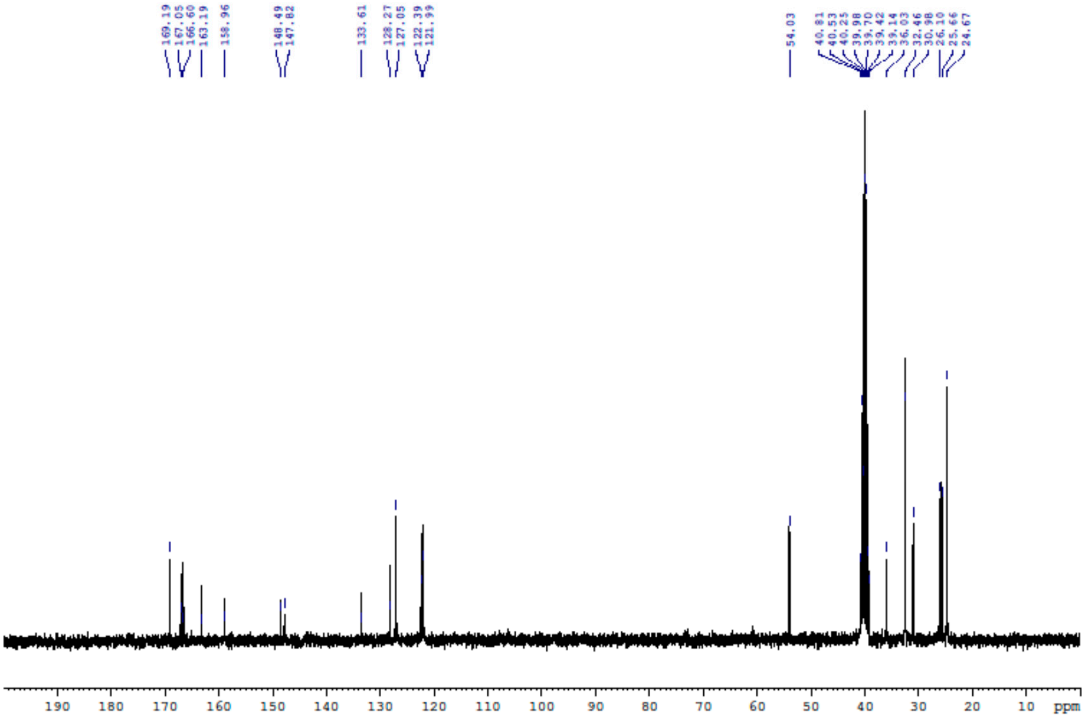

Figure 64. Compound 6p ^{13}C NMR spectrum

SHIMADZU

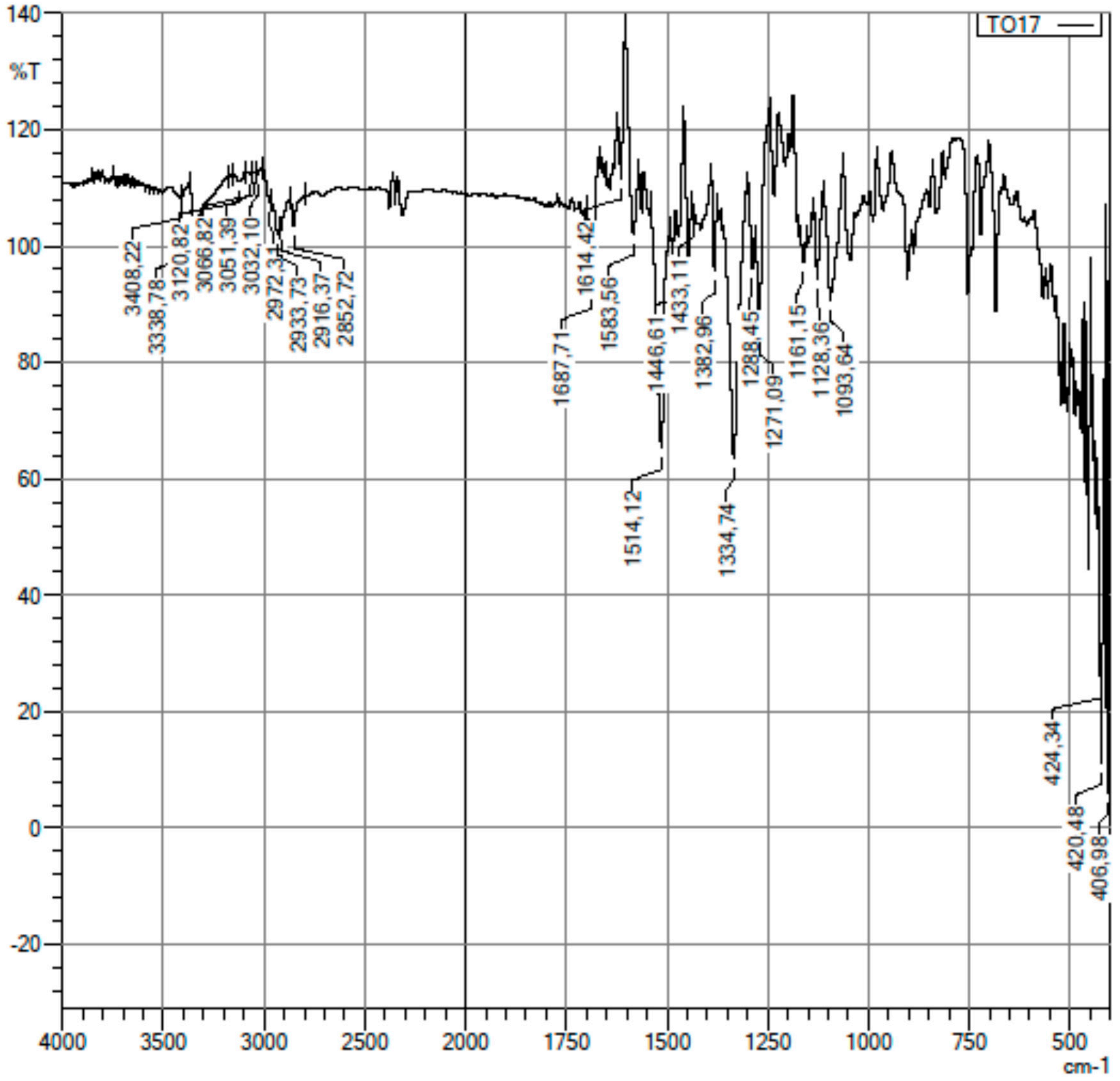

C:¥Users¥dopnalab¥Desktop¥BETﾌL¥TO¥TO17.ispd

	Item	Value
2	Sample name	TO-17
3	Sample ID	
4	Option	
5	Intensity Mode	%Transmittance
6	Apodization	Happ-Genzel
9	No. of Scans	10

1

Figure 65. Compound 6r IR spectrum

Data File: C:\LabSolutions\Data\Analiz\BKaya\TO17_19.lcd

Elmt	Val.	Min	Max	Elmt	Val.	Min	Max	Elmt	Val.	Min	Max	Use Adduct
H	1	10	40	O	2	4	5	Cl	1	0	1	H
C	4	10	26	F	1	0	0	Br	1	0	0	
N	3	8	8	S	2	4	4					

Error Margin (ppm):	5	DBE Range:	9.0 - 19.0	Electron Ions:	both
HC Ratio:	unlimited	Apply N Rule:	yes	Use MSn Info:	no
Max Isotopes:	3	Isotope RI (%):	1.00	Isotope Res:	10000
MSn Iso RI (%):	10.00	MSn Logic Mode:	AND	Max Results:	500

Event#: 1 MS(E+) Ret. Time : 8.920 -> 8.960 Scan# : 1339 -> 1345

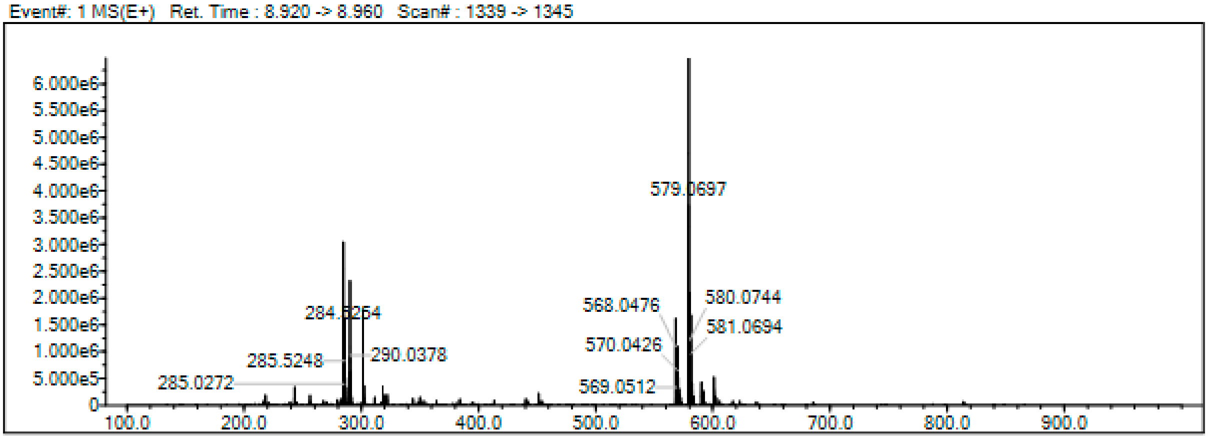

Measured region for 579.0697 m/z

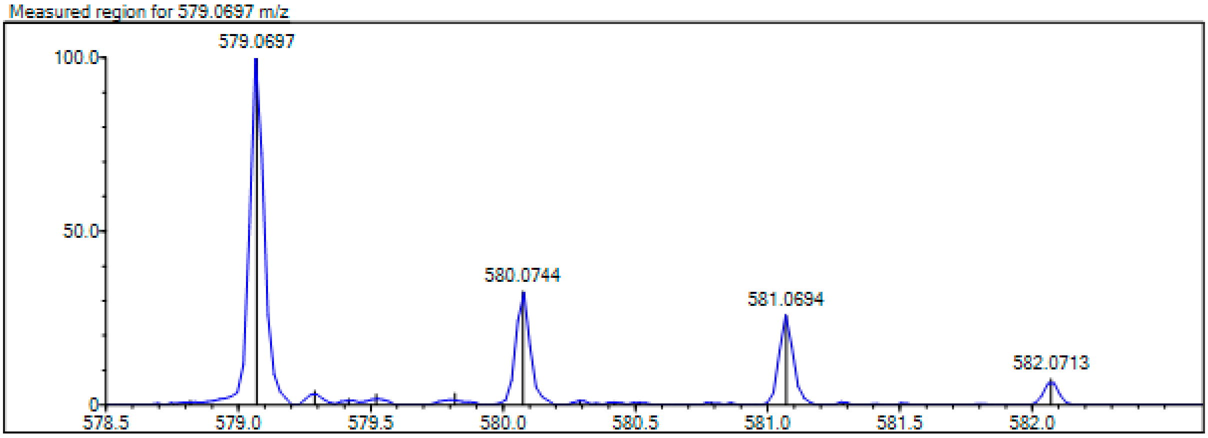

C21 H22 N8 O4 S4 [M+H]+ : Predicted region for 579.0720 m/z

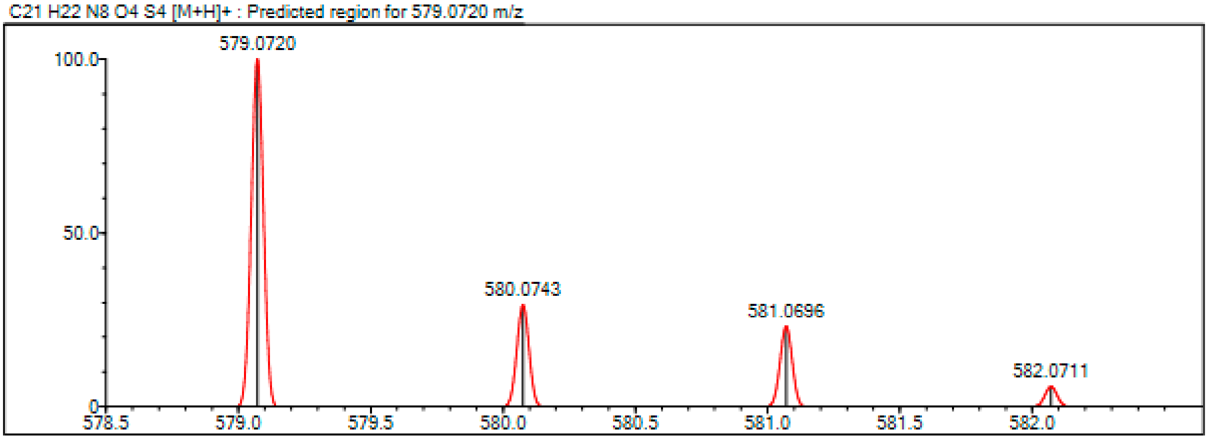

Rank	Score	Formula (M)	Ion	Meas. m/z	Pred. m/z	Df. (mDa)	Df. (ppm)	Iso	DBE
1	78.85	C21 H22 N8 O4 S4	[M+H]+	579.0697	579.0720	-2.3	-3.97	85.17	15.0

Figure 66. Compound 6r Mass spectrum

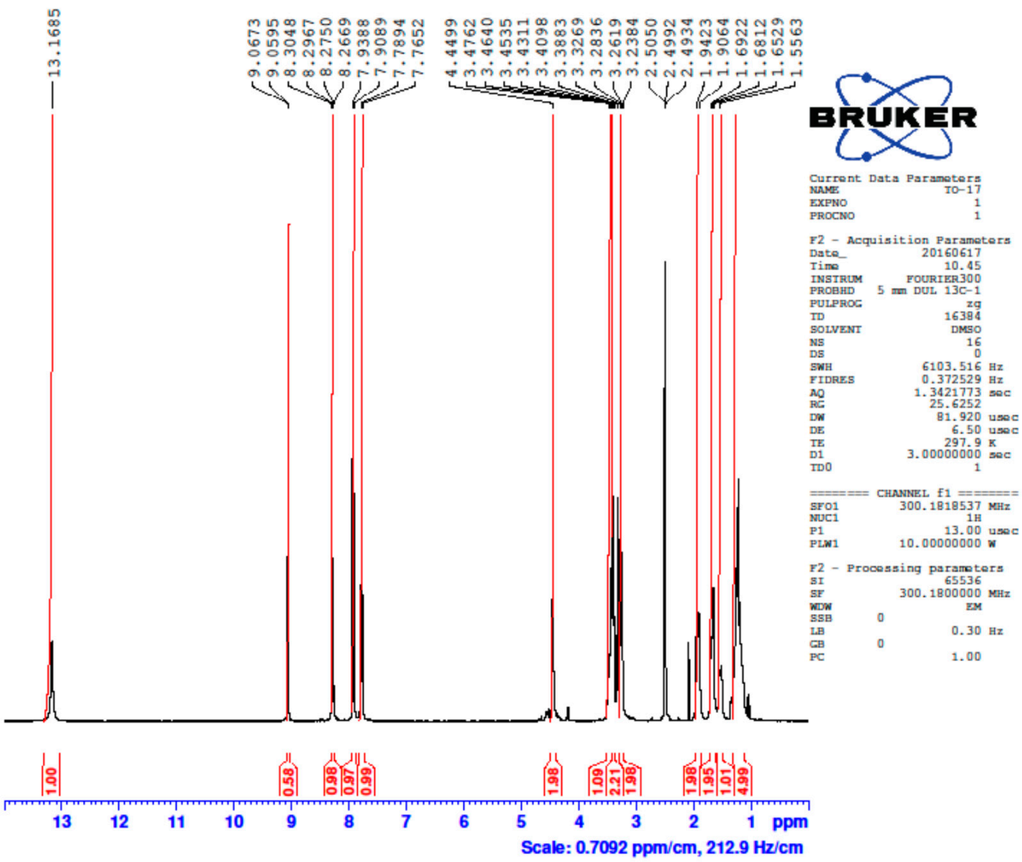

Figure 67. Compound 6r ^{1}H NMR spectrum

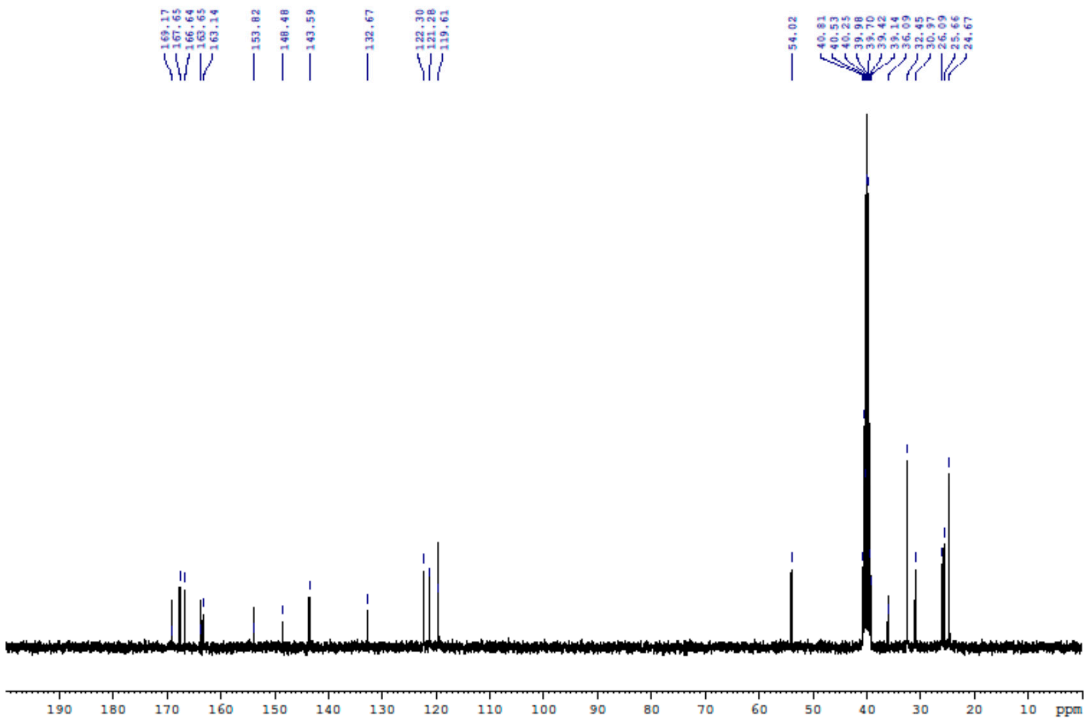

Figure 68. Compound 6r ^{13}C NMR spectrum

SHIMADZU

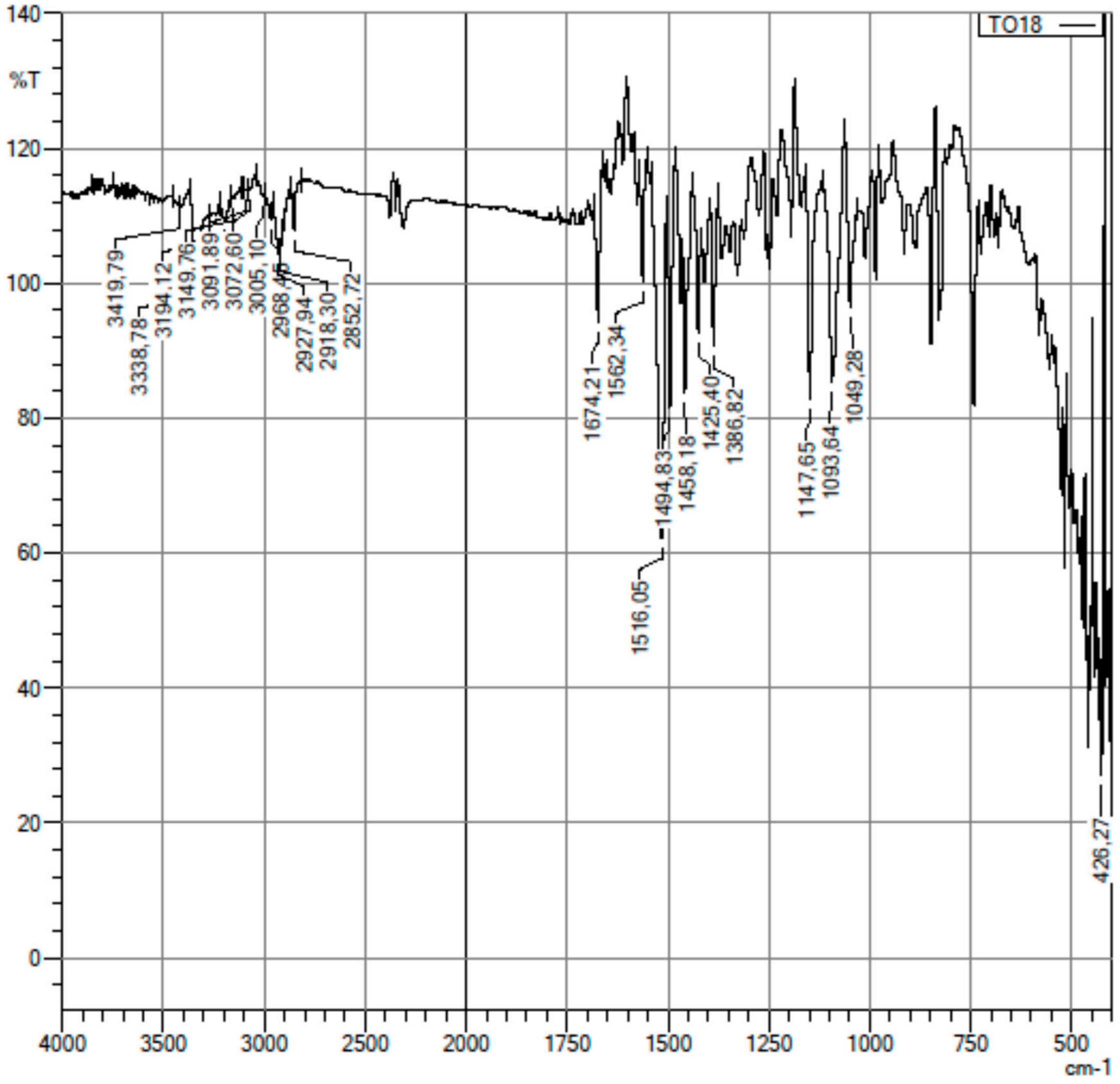

C:¥Users¥dopnalab¥Desktop¥BETワL¥TO¥TO18.ispd

	Item	Value
2	Sample name	TO-18
3	Sample ID	
4	Option	
5	Intensity Mode	%Transmittance
6	Apodization	Happ-Genzel
9	No. of Scans	10

Figure 69. Compound 6s IR spectrum

Formula Predictor Report - TO18_20.lcd

Data File: C:\LabSolutions\Data\Analiz\BKaya\TO18_20.lcd

Elmt	Val.	Min	Max	Elmt	Val.	Min	Max	Elmt	Val.	Min	Max	Use Adduct
H	1	10	40	O	2	2	5	Cl	1	0	1	H
C	4	10	26	F	1	1	1	Br	1	0	0	
N	3	7	8	S	2	4	4					

Error Margin (ppm):	5	DBE Range:	9.0 - 19.0	Electron Ions:	both
HC Ratio:	unlimited	Apply N Rule:	yes	Use MSn Info:	no
Max Isotopes:	3	Isotope RI (%):	1.00	Isotope Res:	10000
MSn Iso RI (%):	10.00	MSn Logic Mode:	AND	Max Results:	500

Event#: 1 MS(E+) Ret. Time : 7.867 -> 7.973 Scan# : 1181 -> 1197

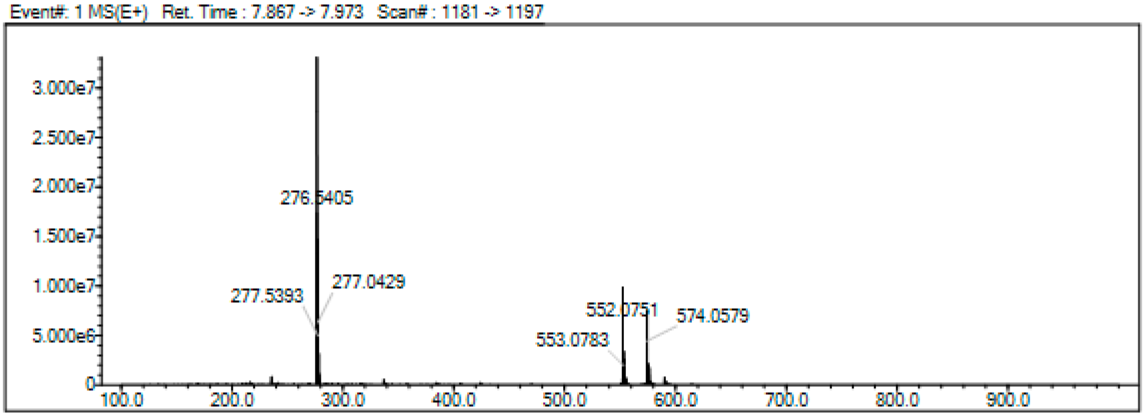

Measured region for 552.0751 m/z

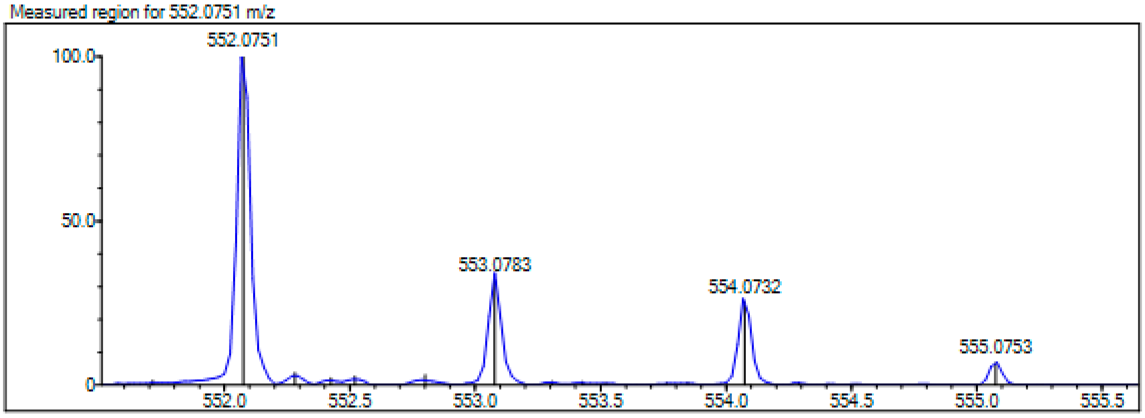

C21 H22 N7 O2 F S4 [M+H]+ : Predicted region for 552.0775 m/z

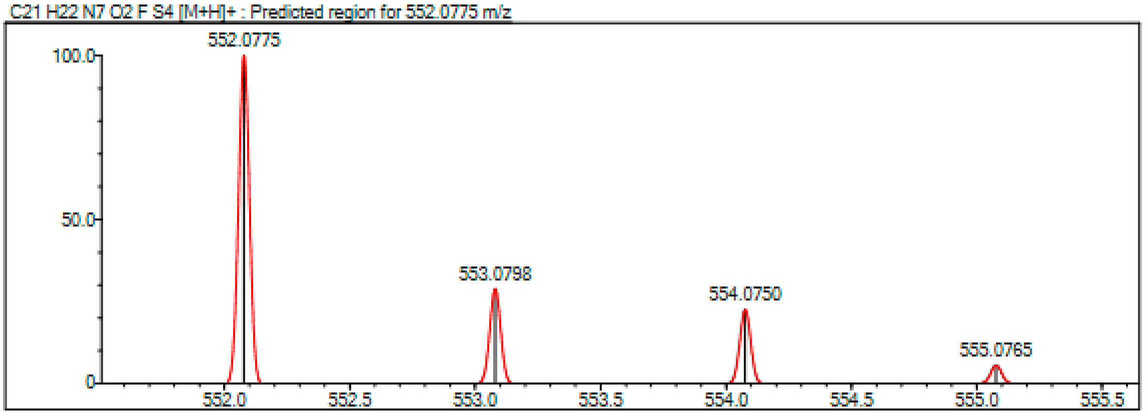

Rank	Score	Formula (M)	Ion	Meas. m/z	Pred. m/z	Df. (mDa)	Df. (ppm)	Iso	DBE
1	79.36	C21 H22 N7 O2 F S4	[M+H]+	552.0751	552.0775	-2.4	-4.35	86.62	14.0

Figure 70. Compound 6s Mass spectrum

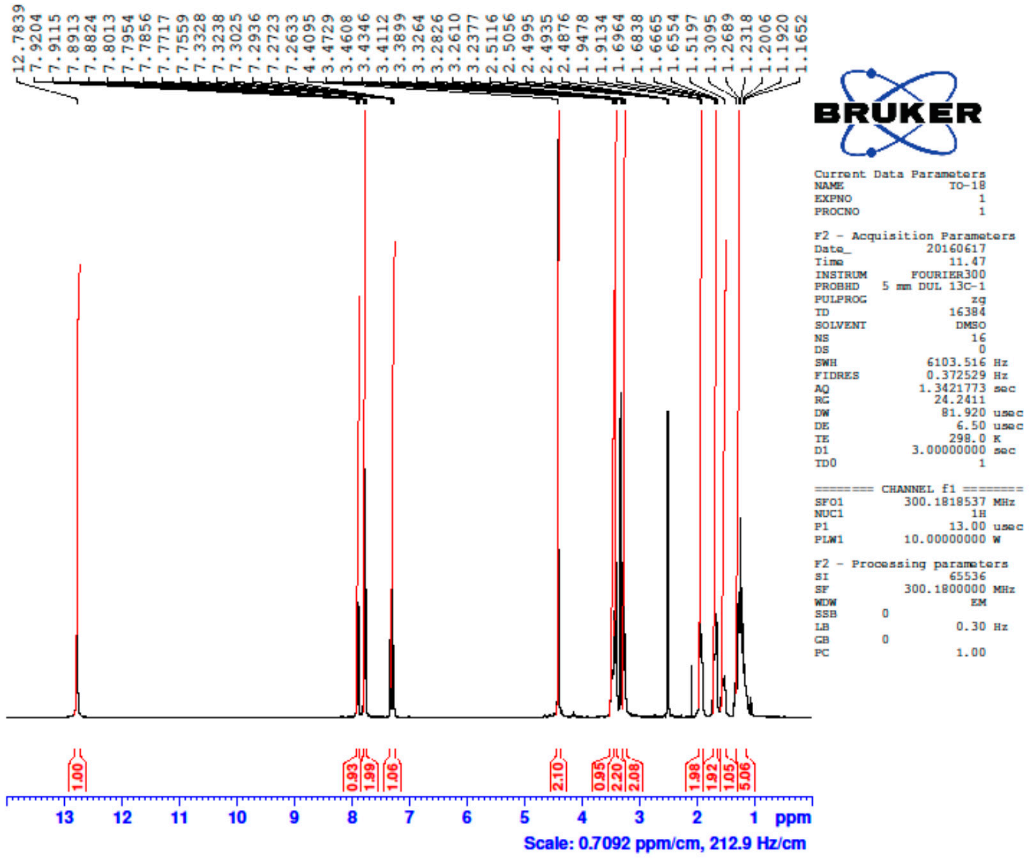

Figure 71. Compound 6s ^{1}H NMR spectrum

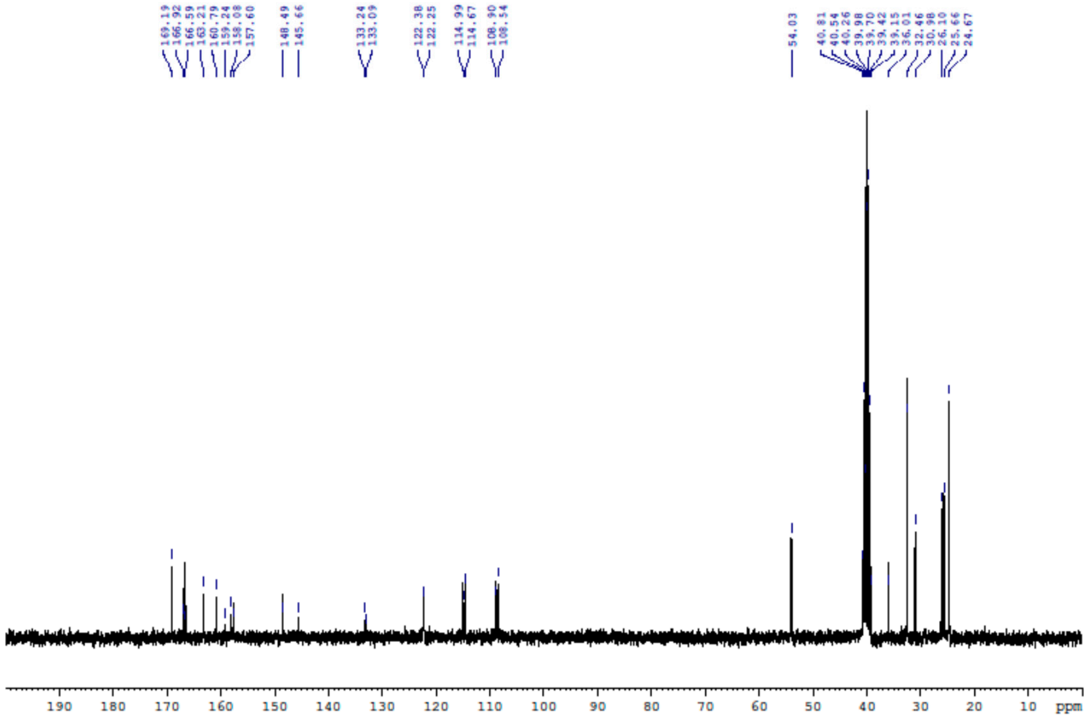

Figure 72. Compound 6s ^{13}C NMR spectrum
